# Women’s priorities towards ovarian cancer testing: a best–worst scaling study

**DOI:** 10.1136/bmjopen-2022-061625

**Published:** 2022-09-01

**Authors:** Rebekah Hall, Antonieta Medina-Lara, Willie Hamilton, Anne Spencer

**Affiliations:** 1 Health Economics Group, University of Exeter Medical School, Exeter, UK; 2 Primary Care Diagnostics, University of Exeter Medical School, Exeter, UK

**Keywords:** Gynaecological oncology, Health economics, Gynaecological oncology, Ultrasound

## Abstract

**Objective:**

To investigate the importance of key characteristics relating to diagnostic testing for ovarian cancer and to understand how previous test experience influences priorities.

**Design:**

Case 1 best–worst scaling embedded in an online survey.

**Setting:**

Primary care diagnostic testing in England and Wales.

**Participants:**

150 women with ovaries over 40 years old living in England and Wales.

**Methods:**

We used best–worst scaling, a preference-based survey method, to elicit the relative importance of 25 characteristics relating to ovarian cancer testing following a systematic review. Responses were modelled using conditional logit regression. Subgroup analysis investigated variations based on testing history.

**Main outcome measures:**

Relative importance scores.

**Results:**

‘Chance of dying from ovarian cancer’ (0.380, 95% CI 0.26 to 0.49) was the most important factor to respondents, closely followed by ‘test sensitivity’ (0.308, 95% CI 0.21 to 0.40). In contrast, ‘time away from usual activities’ (−0.244, 95% CI −0.33 to −0.15) and ‘gender of healthcare provider’ (−0.243, 95% CI −0.35 to −0.14) were least important to respondents overall. Women who had previously undergone testing placed higher importance on certain characteristics including ‘openness of healthcare providers’ and ‘chance of diagnosing another condition’ at the expense of reduced emphasis on characteristics such as ‘pain and discomfort’ and ‘time away from usual activities’.

**Conclusions:**

The results clearly demonstrated items at the extreme, which were most and least important to women considering ovarian cancer testing. Differences in priorities by testing history demonstrate an experience effect, whereby preferences adapt over time based on evidence and experience. Acknowledging these differences helps to identify underlying barriers and facilitators for women with no test experience as well as shortcomings of current service based on women with experience.

**STRENGTHS AND LIMITATIONS OF THIS STUDY**

- This study adds to a very limited evidence base of studies assessing priorities towards diagnostic testing for cancer and specifically ovarian cancer.
- Selection of included characteristics is based on a rigorous, published systematic review and used patient and public involvement to ensure the relevance to the target population.
- Case 1 best–worst scaling is ideal for establishing the relative importance of a large number of characteristics and has been proven to be easier to complete and more effective than alternative methods such as ranking or ratings tasks.
- A key limitation of the study relates to the representativeness of the sample. Due to the recruitment method, the sample is not fully representative of the population in key demographics including ethnicity and age distribution.
- The lack of discrimination between lower scoring attributes may be reflective of genuine priorities. However, it is also possible that choice task construction and sample size were contributing factors.

## Introduction

Ovarian cancer is the seventh most common cancer in women worldwide, with over 200 000 new cases and approximately 180 000 deaths annually.^1^ Five-year survival rates for the disease are highly dependent on a number of factors including the patient’s age, country of residence and tumour type.^2^ Late-stage diagnosis contributes heavily to the high mortality rates associated with ovarian cancer and is an ongoing challenge globally.^3^ For instance, in the UK, almost 60% of cases are diagnosed at stage III or IV where average 5-year survival is just 26.9% and 13.4%, respectively.^4^ Improving diagnostic outcomes is multifaceted problem, however, delays in help-seeking on symptom onset and access to timely testing have been identified as challenges to earlier diagnosis.^5^

National guidelines for the investigation of suspected ovarian cancer in symptomatic women vary substantially between countries.^6^ Existing tests include the CA125 blood test and imaging tests, most commonly a transabdominal/transvaginal ultrasound (TVUS) but also CT and MRI.^7^ Variations in guidance represents uncertainty around the accuracy of existing test strategies, especially for the early investigation of symptoms. Evidence evaluating the performance of diagnostic tests in a primary care setting is very limited.^8^ Furthermore, for recommendations to be effective, clinical guidelines must also consider the preferences of those offered testing, particularly in healthcare systems emphasising shared-decision making.^9^ Available tests differ substantially, not only in terms of performance but also service delivery and patient experience. Aspects of tests may be prioritised differently; for instance, patients may accept a lower levels of accuracy a less invasive test. Thus, it may be necessary to weight characteristics differently when considering the overall balance of benefits and harms.

To date, very little attention has been paid to the priorities of women facing ovarian cancer testing, particularly in a diagnostic setting. Existing studies usually focus on screening trials,^10^ single test modalities^11^ or single aspects of acceptability, such as pain.^12^ To address the current evidence gap, we aimed to elicit preferences for 25 key characteristics (‘items’) of ovarian cancer testing, using best–worst scaling (BWS). This is a stated preference technique that has been demonstrated to provide higher predictive values than ranking or rating methods while being less cognitively demanding.^13^ Understanding the importance of key characteristics allows aspects of greatest importance to be given increased salience in future decisions about testing, particularly in guideline revision.

Previous studies have demonstrated views may differ based on previous experience of the health event. We; therefore, examined the relationship between priorities around ovarian cancer testing and test experience.^14 15^

## Methods

### Patient and public involvement in the research

The survey was shared and discussed with the Cancer Research UK-funded CanTest PPI lead, Margaret Johnson. Amendments in survey wording were made as a result of discussions.

### Best–worst scaling

We used case 1 BWS method to identify women’s preferences for characteristics associated with diagnostic testing for ovarian cancer. This type of BWS aims to assess the relative importance of, or preference for, items based on the underlying principles of random utility theory.^16^ BWS was initially developed in marketing but has been increasingly used in healthcare research for explorations of patient and stakeholder preferences.^16–18^ In particular, case 1 BWS has been demonstrated to be an effective alternative to traditionally used ranking or ratings tasks when considering patient priorities.^19^

During BWS tasks, participants respond to a series of choice tasks presenting a subset of items and asked to select the ‘best’ or ‘most important’ and ‘worst’ or ‘least important’ item. Simultaneously examining items selected as ‘most’ and ‘least’ important provides greater information than examining the most important item alone. Analysing responses to choice tasks allows the underlying relative importance of items to be inferred and a ranking of included items to be established.^19^

### Identification of relevant items

Results demonstrate the importance of an item, relative to the other included item. It is therefore important to use a rigorous selection process to ensure the most salient characteristics are included in the experiment. We performed a systematic review of the preference-based literature to identify characteristics relating to cancer testing.^20^ Potential characteristics were then narrowed down by the authors based on relevance to ovarian cancer testing in symptomatic patients using an iterative Delphi method process where exclusion required full agreement of the research team. In total, 25 characteristics were selected for inclusion (table 1).

**Table 1:** Characteristics included in the BWS study (wording and descriptions are identical to those shown to respondents during the survey)

	Characteristic and definition
1	Test sensitivity Chance that the test will miss cancer in a patient who actually does have the disease (false-negative result)
2	Chance of dying from ovarian cancer How much having the test decreases the chance of dying from ovarian cancer
3	Choice of appointment time Whether a person can choose an appointment time or if the appointment time is assigned by the healthcare provider
4	Who explains the results Type of healthcare provider who explains the test results, for example, nurse, doctor, etc.
5	Pain and discomfort The level of pain and/or discomfort experienced during and after the test
6	Notification of negative test results Whether you are contacted if your results are normal
7	Chance of diagnosing another condition If symptoms are not caused by cancer, the chance the test can identify other reason for the symptoms
8	Pretest support Level of support received before having the test describing what will happen during the test and what the results might show
9	Test procedure What having the test will involve. For ovarian cancer this could be a blood test or an transvaginal ultrasound (internal ultrasound of the reproductive organs)
10	Staff attitude How the healthcare provider treats you while conducting the test
11	Post-test support Level of support received after getting the results of the test relating to the meaning of your results and what will happen next
12	Time away from usual activities The total amount of time spent having the test instead of doing your usual daily activities
13	Test specificity Chance of unnecessary further invasive testing (false-positive result)
14	Travel time The total amount of time spent travelling to and from the test
15	Time to notification of results The length of time it takes to hear the results after having the test
16	Openness of healthcare providers How open healthcare providers are with their thoughts about the cause of your symptoms and the tests they recommend
17	No of follow-up tests How many additional tests are needed to confirm a diagnosis
18	Chance of an inconclusive result The chance the results are unclear and the test would need to be repeated after a waiting period
19	Out of pocket costs How much it will personally cost a person to have the test, for example, travel costs, childcare costs, time off work, etc. The cost of the test is covered by the NHS
20	Gender of healthcare provider Gender of the staff member giving you the test
21	How test results are returned, for example, in person, phone, letter
22	Test location Where the test takes place
23	Test duration The length of time spent having the test
24	Information included with the invitation The level and type of information received about the test
25	Waiting time for the test How long a person has to wait to have the test after being referred by their GP

BWS, best–worst scaling; GP, general practitioner; NHS, National Health Service.

### BWS task construction

**Figure 1:**
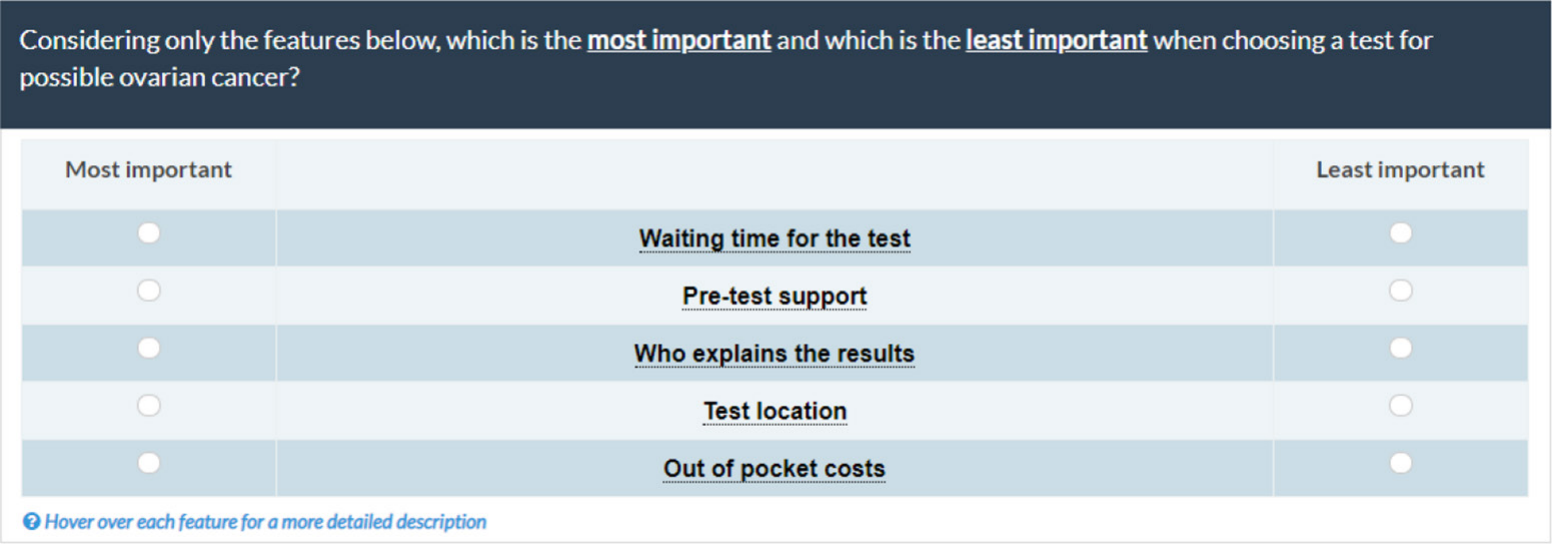
Example of a choice task.

A balanced incomplete block design (BIBD) generated using SAS V.9.4 was used to construct the BWS choice sets for the 25 items. BIBD designs ensure each item appears equally often, and pairwise comparisons between each of the items occurs equally across the design.^21^ A final design (d-efficiency of 83.3%) consisting of 30 choice tasks each with five items was selected based on a trade-off between survey complexity and design efficiency. Each item appeared six times across the choice tasks and coappeared with remaining attributes once throughout the experiment. The position of the item within choice tasks was optimised such that items were listed in 1st–5th position equally and the order of tasks was randomised across participants to control for any order effects. Each participant completed all 30 tasks. An example of a BWS task is shown in figure 1.

### Questionnaire

The questionnaire was developed and collected using LimeSurvey, an online survey platform. The survey consisted of four stages as follows: (1) Sociodemographic questions, for example, age, education, employment status; (2) BWS questions (including a warm-up task); (3) Task feedback questions, for example, task difficulty and (4) Background questions relating to health-related characteristics, for example, self-reported health, testing history, current and desired in medical decisions.

Question framing and answer categories for stages 1 and 3–4 were copied or adapted from established national surveys. Early piloting with five women suggested the survey would take 30 min to complete. Given the length of the survey and online administration method, three attention checks following the instructional manipulation format were embedded (eg, ‘Select ‘very important’ to indicate you are paying attention’).^22^ Respondents who failed all three attention checks were removed from the analysis. A full version of the questionnaire is available on request.

### Participants

A sample size of 150 women was estimated to be required. Participants were recruited via Prolific, an online platform for researchers conducting social science experiments. Results from this platform have been shown to be of comparable or better quality than university research lab registers and have been used widely within hundreds of published studies across disciplines.^23 24^ Our study focused on the priorities of women over 40 (no upper limit), the group most likely to be offered testing.^25^ Participation was also limited to those living in England and Wales: no other limitations were applied. Respondents completed an electronic consent form before completing any questions.

### Statistical analysis

Descriptive statistics relating to sociodemographic and health-related characteristics of the sample were summarised.

Best–worst responses were initially analysed using the counting approach; whereby the number of times an item was picked as ‘least important’ is subtracted from the number of times it was chosen as ‘most important’. Each item appeared six times across all tasks, meaning best–worst scores could range from −6 to +6 at the individual level. Individual scores were aggregated and standardised to calculate an overall mean score for the sample ranging from −1 to +1 (Scores were standardised using the following equation: population-level best–worst score/(number of times item appeared × total sample size)). A score close to +1 indicates an item is highly influential, whereas item with scores near to −1 demonstrate much less relevance.

Next, conditional logit regression using dummy-coded items was used to model responses. ‘Time away from usual activities’ was identified as the least important attribute during counting analysis and was omitted from the model and used as the reference item. As a result, all parameters were expected to be positive. To aid interpretation, we rescaled conditional logit coefficients using standardised ratio scores where all scores sum to 100.^26 27^ CIs for relative importance scores were estimated using the delta method. All analyses were performed using Stata V.17.^28^

In both the counting and conditional logit analysis, scores relate to the relative importance of attributes (ie, relative importance scores). In other words, differences between scores are meaningful whereas absolute values are not.

#### Subgroup analysis

Subgroup analysis was performed to explore the relationship between previous testing and preferences for future testing. Experience was captured by two questions that asked whether women had previously: (1) undergone testing for ovarian cancer or (2) received a transvaginal ultrasound for any reason. Subgroup analysis was based on conditional logit results and differences in relative importance scores between groups were tested using unpaired t-tests. Finally, heteroscedastic logit models were estimated to investigate whether differences between subgroup were attributable to scale differences (ie, differences in error variance between subgroups) or due to a genuine difference in preferences.

## Results

In total, 159 women responded to the survey. The average response time for the questionnaire was 29 min 51 s. Four submissions were incomplete, two were removed due to failing all attention checks, and a further three responses were removed due to incorrect completion of the best–worst section of the survey, resulting in 150 responses for the final analysis. Respondents varied substantially in how difficult they found the best–worst portion of the questionnaire with 42% (63/150) reporting it easy/very easy but 38% (57/150) finding it difficult/very difficult. There were no significant differences in BWS responses between those who found the task difficult versus those who did not.

### Demographics

The demographics of the sample are presented in table 2. The age ranged from 40 to 87 years old with a mean of 51.4 (SD=9.1). Most participants were white (120/150; 80%), married/in a relationship (97/150; 65%) and employed (78/150; 52%).

**Table 2:** Descriptive characteristics: sociodemographic

Characteristic	n (%)
Age	
Mean (SD)	51.4 (9)
Range	40–87
Ethnicity	
White	120 (80)
Asian	8 (5)
Black	3 (2)
Mixed	3 (2)
Other	9 (6)
Not reported	7 (4)
No of children, mean (SD)	1.3 (1)
Relationship status	
Married	75 (50)
In a relationship	22 (15)
Single	19 (13)
Divorced/separated	26 (17)
Widowed	6 (4)
Not reported	2 (1)
Level of education	
GCSE	37 (25)
A-level/college	25 (17)
Undergraduate	41 (27)
Postgraduate	35 (23)
No qualifications	1 (1)
Other	9 (6)
Not reported	2 (1)
Employment status	
Full-time	47 (31)
Part-time	32 (21)
Self-employed	23 (15)
Not employed	11 (7)
Retired	14 (9)
Other	18 (12)
Not reported	5 (3)

GCSE, General Certificate of Secondary Education.

Most women perceived their risk of cancer as low-average (128/150; 85%) and ovarian cancer-related anxiety was generally low-moderate among respondents (116/150; 77%). Overall, 50 women (33%) reported previously undergoing a TVUS for any reason. Forty (27%) women reported being previously tested for ovarian cancer, with CA125 blood test being the most common test.

Crucially, when asked, 127/150 women (89%) stated they wished for a great deal/a lot of involvement in decisions relation to their own care but only 34/150 (23%) currently felt this was achieved, with 17/150 (11%) respondents felt unable to be involved in medical decisions at all. Further details are found in online supplemental appendix 1.

10.1136/bmjopen-2022-061625.supp1 Supplementary data

### Best–worst results

#### Counting analysis

BWS results are presented in online supplemental appendix 2. Scores were bound between −1 and 1. Scores tending towards the extremes of the scale would imply homogeneity across respondents and consistency between responses across questions on an individual level. Importance scores ranged from −0.224 to 0.380, suggesting high levels of heterogeneity in preferences regarding test characteristics across respondents (see online supplemental appendix 3).

Overall, ‘chance of dying from ovarian cancer’ (0.380, 5% CI 0.26 to 0.49) was most important to women when considering ovarian cancer testing, followed by ‘test sensitivity’ (0.308, 95% CI 0.21 to 0.40). Conversely, ‘time away from usual activities’ (−0.244, 95% CI −0.33 to −0.15) and ‘gender of healthcare provider’ (−0.243, 95% CI −0.35 to −0.14) were considered least important to women when facing diagnostic testing and were statistically indistinguishable from each other.

#### Conditional logit analysis

The results for the conditional logit model are shown in online supplemental appendix 2. The order of importance remained consistent across the two analysis methods and the estimates were highly correlated (online supplemental appendix 4).

All items had a positive coefficient and most were statistically significant at the 95% level, confirming the relative importance of all attributes compared with ‘time away from usual activities’. Non-significant items were those with the lowest importance (eg, ‘gender of healthcare providers’ and ‘how test results are returned’), suggesting a clustering effect towards the bottom of the importance scale.

**Figure 2:**
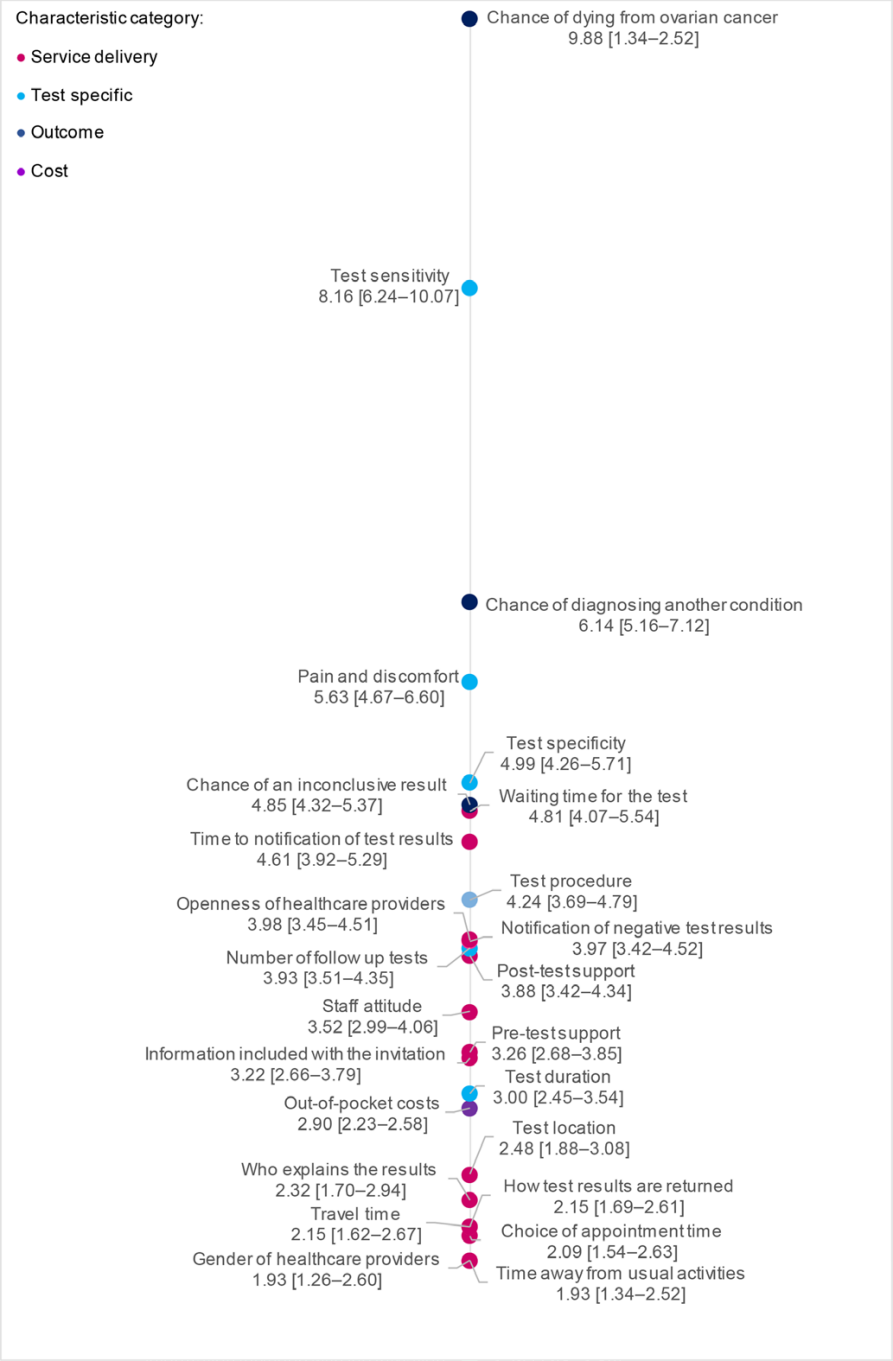
Best–worst scaling results. The distance between attributes is a spatial representation of the difference in relative importance between attributes on the latent importance scale.

Figure 2 shows the relative importance scores associated with the conditional logit estimates. The distance between each item is a spatial visualisation of differences in relative importance. Importance scores ranged from a maximum of 9.88 (95% CI 7.04 to 12.72) for ‘chance of dying of ovarian cancer’ to a minimum of 1.93 (95% CI 1.34 to 2.52) for ‘time away from usual activities’ and Gender of healthcare providers. Indicating ‘chance of dying from ovarian cancer’ was approximately five times more important to respondents. The most important characteristics to respondents were clear and distinct: however, spatial visualisation demonstrated grouping of attributes towards the centre and bottom of the scale. Groupings were distinct to other items but differences in importance between items within clusters is less distinguishable. In general, results demonstrate a clear prioritisation of outcome (dark blue dots) and test specific characteristics (light blue dots) whereas service delivery characteristics (pink dots) were consistently less important to respondents.

#### Subgroup analysis

**Figure 3:**
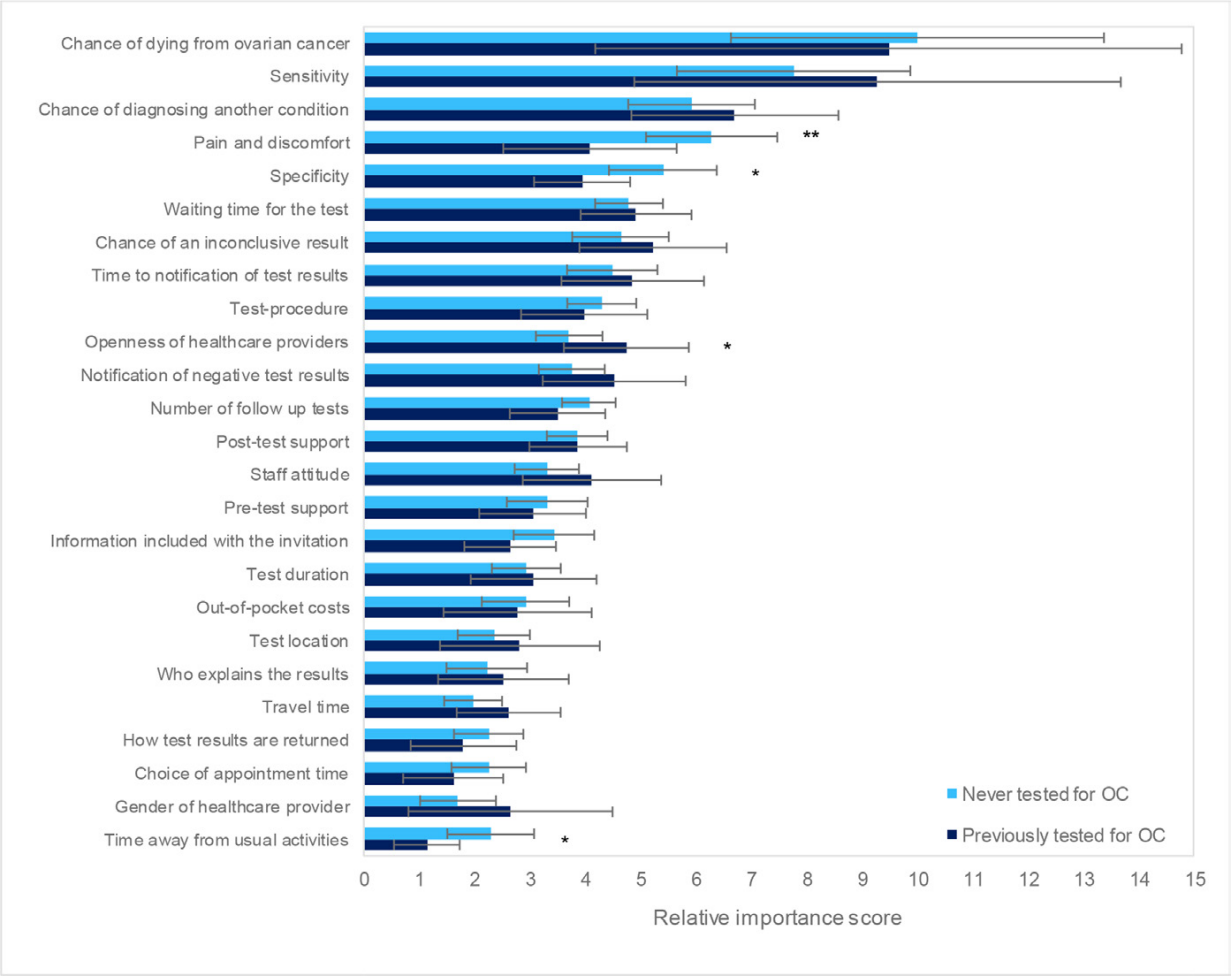
Subgroup analysis results comparing importance scores between those previously tested for ovarian cancer (OC) (n=40) and those who have never been tested (n=110). Error bars represent 95% CIs.Significance of differences between subgroups: *p<0.10, **p<0.05, ***p<0.01

Women previously tested for ovarian cancer placed significantly lower importance on ‘pain and discomfort’ in comparison to test-naïve individuals. There was also evidence to suggest ‘time away from usual activities’ and ‘test specificity’ were less prioritised by previously tested women while ‘openness of healthcare providers’ and ‘test specificity’ appeared marginally more important to those with test experience (figure 3).

**Figure 4:**
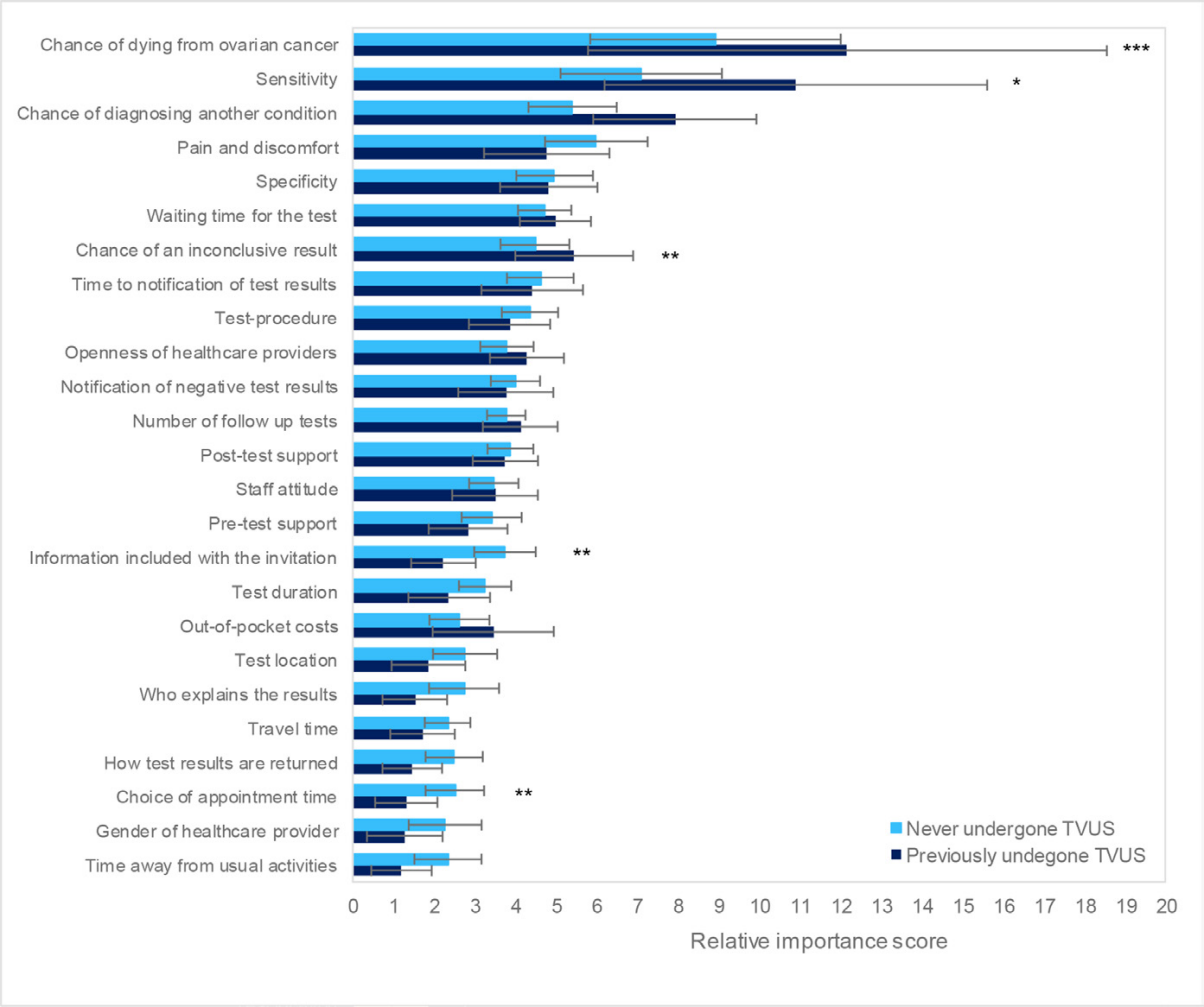
Subgroup analysis results comparing importance scores between those who have previously undergone a transvaginal ultrasound (TVUS) for any medical reason (n=50) and those who have not (n=100). Error bars represent 95% CIs.Significance of differences between subgroups: *p<0.10, **p<0.05, ***p<0.01

For women who had previously undergone a TVUS, ‘chance of diagnosing another condition’ and ‘chance of dying from ovarian cancer’ were significantly more important compared with those with no test experience (figure 4). Alternatively, women who had never been tested appeared to place higher value on ‘information included with the invitation’ and ‘choice of appointment time’, although the overall importance of such attributes remained relatively low.

In both instances, results from heteroscedastic logit models demonstrated no differences in scale between subgroups indicating observed differences were attributable to differences in priorities as opposed to differences in response variance between groups with different prior experience (online supplemental appendix 5).

## Discussion

### Summary of main findings

This is the first study to investigate the priorities of women relating to diagnostic testing for ovarian cancer. The results of this study highlight the importance yet current neglect of incorporating the preferences of patients into medical testing decisions. When asked, almost 90% (134/150) of respondents wanted heavy involvement in medical decisions: however, less than a quarter of women currently felt able to do so.

The results are particularly useful in identifying items at either extreme of the scale, clearly demonstrating the characteristics that are most and least important to women considering ovarian cancer. Low concurrence between items and high levels of heterogeneity meant discrimination between mid-range items was less clear. Overall, ‘chance of dying from ovarian cancer’, ‘test sensitivity’ and ‘chance of diagnosing another condition’ were the most important characteristics when considering testing for ovarian cancer. Oppositely, ‘time away from usual activities’ and ‘gender of healthcare provider’ were the least important factors. The priorities of previously tested women were generally similar to women who had never undergone testing, but there were a few key differences between these two groups suggesting experience is an important determinant of priorities.

### Results in the context of published literature

The prioritisation of outcome and test-specific characteristics and the relative lack of importance placed on service delivery characteristics is reflective of findings from the wider literature examining preferences towards cancer screening and diagnosis.^20 29–32^ The relative importance of ‘openness of healthcare providers’ is echoed by qualitative research exploring the diagnostic experiences of women with ovarian cancer where shortcomings in doctor–patient communication was a recurring theme. In particular, patients raised concerns about doctors’ willingness and ability to openly communicate and share information.^33 34^ Decreased importance of particular characteristics to tested women may suggest these aspects of testing are already being achieved/acceptable within the current system. For example, the decreased importance of ‘pain and discomfort’ for both women who have been tested for ovarian cancer and women who have previously received an ultrasound (NS) is expected given the finding that almost 80% of women who had previously undergone a transvaginal ultrasound experienced little to no pain.^12^

Existing studies examining differences in preferences towards cancer testing between previously tested and untested individuals have typically focused on colorectal cancer screening, but also found evidence of statistically different preferences between the two groups.^35 36^ Hol *et al*^35^ found previously screened individuals displayed stronger and more positive preferences towards different screening modalities and shorter screening intervals than unscreened counterparts. In a similar study, van Dam *et al*^36^ found limited differences between people with and without screening experience (mortality risk reduction only), however, preferences across previously screened individuals differed significantly based on the particular screening modality received. These results are evidence of an experience effect, via ‘status quo bias’, where individuals place a higher value on goods or services that are more familiar.^37^

### Strengths and limitations

BWS provides a straightforward method for capturing the priorities of women. The method is preferable to ranking/rating tasks due to its ability to measure the importance of large numbers of items while limiting complexity and cognitive burden.^38^

The survey length and high number of items represent a potential limitation. In total, 38% of respondents reported the task as difficult/very difficult. Despite this, drop-out rates were low (3% (4/150) of people began the survey but did not complete) and there were no differences between responses according to reported task difficulty.

Poor discrimination between lower scoring items may be reflective of genuine equality in priorities, however, it is also feasible that the choice task construction was a contributing factor. For example, the ability to detect differences will be affected by the number of pairwise comparisons between attributes. Selection of a BIBD with more than one pairwise comparison between each item may have increased the explanatory power of the study, of course this requires a trade-off with survey length and sample size requirements where blocking is required. Similarly, a larger sample size may have increased the explanatory power and also allowed for more complex statistical investigation of preference heterogeneity. To our knowledge, there is currently no accepted guidance on sample size requirements within object case BWS, although theories from the closely related methods, such as discrete choice experiments may be transferable.^39^

Finally, due to the recruitment method, the sample is not fully representative of the population in key demographics including ethnicity and age distribution. Further research is needed to understand whether results are generalisable to a wider population.

### Implications for practice and future research

The findings of this study offer useful insights into potential barriers and facilitators of undergoing testing in a timely manner. Interestingly, characteristics involving an element of risk or uncertainty dominated the top-ranking positions. However, how to best explain complex aspects of test performance such as sensitivity and specificity to patients is a clear challenge.^40^

Differences in importance between women with and without test experience suggest that priorities are continuously adapted based on evidence and experience gained over time. When considering policy decisions, it is therefore important to carefully consider whose views should be prioritised—a long-standing debate within the field of health technology assessment.^41^ Arguably, it is important to consider both perspectives; priorities of women with test experience help to identify unmet or inadequate aspects of current service provision, whereas preconceived views of testing-naïve women may reveal underlying barriers and facilitators of testing since the initial decision to undergo testing is based on these pre-existing judgements. In both instances, mismatches in priorities and practice may lead to delays in seeking help or testing for future symptoms.

High levels of heterogeneity between individuals highlight the importance of a personalised approach to patient interactions throughout the diagnostic process. Patients are likely to have different concerns and priorities during this time, however, the importance of patient-input and shared decision-making appears to be less prioritised in diagnostic settings compared with decisions regarding cancer screening and subsequent treatment where preferences have been studied more extensively.

## Conclusion

Preferences towards diagnostic testing have been underexplored to date. Understanding what matters most to patients may reduce anxiety around testing, facilitate earlier help-seeking behaviour and improve patient satisfaction. This study highlights that test sensitivity and mortality impacts are the most important factors to patients facing ovarian cancer testing. However, results varied significantly across individuals demonstrating the need for an individualised approach to consultations regarding diagnostic care. Our results can help inform policies and diagnostic guidelines designed to encourage earlier help-seeking behaviour as well as help to evaluate the patient-friendliness of emerging test strategies for suspected ovarian cancer.

## Supplementary Material

Reviewer comments

Author's manuscript

## Data Availability

Data are available on reasonable request. Data are available on reasonable request. Please contact the corresponding author with any enquiries.

## References

[1] Reid BM, Permuth JB, Sellers TA. Epidemiology of ovarian cancer: a review. Cancer Biol Med 2017;14:9. 10.20892/j.issn.2095-3941.2016.0084 28443200 PMC5365187

[2] Matz M, Coleman MP, Carreira H, et al. Worldwide comparison of ovarian cancer survival: histological group and stage at diagnosis (CONCORD-2). Gynecol Oncol 2017;144:396–404. 10.1016/j.ygyno.2016.11.019 27919574 PMC6195190

[3] Bhatla N, Jones A. The world ovarian cancer coalition atlas, 2018.

[4] Cancer Research UK. Ovarian cancer statistics secondary ovarian cancer statistics, 2019. Available: https://www.cancerresearchuk.org/health-professional/cancer-statistics/statistics-by-cancer-type/ovarian-cancer#heading-Two

[5] Pathfinder TOC. Transforming futures for women with ovarian cancer, 2016.

[6] Funston G, Van Melle M, Baun M-LL, et al. Variation in the initial assessment and investigation for ovarian cancer in symptomatic women: a systematic review of international guidelines. BMC Cancer 2019;19:1028. 10.1186/s12885-019-6211-2 31676000 PMC6823968

[7] NHS UK. Diagnosis: ovarian cancer. Secondary Diagnosis: Ovarian Cancer, 2020. https://www.nhs.uk/conditions/ovarian-cancer/diagnosis/

[8] Sundar S, Neal RD, Kehoe S. Diagnosis of ovarian cancer. BMJ 2015;351:h4443. 10.1136/bmj.h4443 26328593

[9] NICE. The guidelines manual: Process and methods [PMG6. Secondary The guidelines manual: Process and methods [PMG6], 2012. https://www.nice.org.uk/process/pmg6/resources/how-nice-clinical-guidelines-are-developed-an-overview-for-stakeholders-the-public-and-the-nhs-2549708893/chapter/nice-clinical-guidelines

[10] Drescher CW, Nelson J, Peacock S, et al. Compliance of average- and intermediate-risk women to semiannual ovarian cancer screening. Cancer Epidemiol Biomarkers Prev 2004;13:600–6. 10.1158/1055-9965.600.13.4 15066925

[11] Gentry-Maharaj A, Sharma A, Burnell M, et al. Acceptance of transvaginal sonography by postmenopausal women participating in the United Kingdom collaborative trial of ovarian cancer screening. Ultrasound Obstet Gynecol 2013;41:73–9. 10.1002/uog.12262 22791597

[12] Bennett CC, Richards DS. Patient acceptance of endovaginal ultrasound. Ultrasound Obstet Gynecol 2000;15:52–5. 10.1046/j.1469-0705.2000.00010.x 10776013

[13] Hollis G, Westbury C. When is best-worst best? A comparison of best-worst scaling, numeric estimation, and rating scales for collection of semantic norms. Behav Res Methods 2018;50:115–33. 10.3758/s13428-017-1009-0 29322399

[14] Czajkowski M, Hanley N, LaRiviere J. The effects of experience on preferences: theory and empirics for environmental public goods. Am J Agric Econ 2015;97:333–51. 10.1093/ajae/aau087

[15] Neuman T, Neuman E, Neuman S. Explorations of the effect of experience on preferences for a health-care service. J Socio Econ 2010;39:407–19. 10.1016/j.socec.2010.02.005

[16] Flynn TN, Louviere JJ, Peters TJ, et al. Best--worst scaling: what it can do for health care research and how to do it. J Health Econ 2007;26:171–89. 10.1016/j.jhealeco.2006.04.002 16707175

[17] Mühlbacher AC, Kaczynski A, Zweifel P, et al. Experimental measurement of preferences in health and healthcare using best-worst scaling: an overview. Health Econ Rev 2016;6:1–14. 10.1186/s13561-015-0079-x 26743636 PMC4705077

[18] Cheung KL, Wijnen BFM, Hollin IL, et al. Using best-worst scaling to investigate preferences in health care. Pharmacoeconomics 2016;34:1195–209. 10.1007/s40273-016-0429-5 27402349 PMC5110583

[19] Louviere JJ, Flynn TN, Marley AAJ. Best-worst scaling: theory methods and applications. Cambridge University Press, 2015.

[20] Hall R, Medina-Lara A, Hamilton W. Attributes used for cancer screening discrete choice experiments: a systematic review. In: The Patient-Patient-Centered outcomes research, 2021: 1–17. 10.1007/s40271-021-00559-3 34671946

[21] Louviere J, Lings I, Islam T, et al. An introduction to the application of (case 1) best–worst scaling in marketing research. Int J Res Mark 2013;30:292–303. 10.1016/j.ijresmar.2012.10.002

[22] Hauser DJ, Schwarz N. It’s a Trap! Instructional manipulation checks prompt systematic thinking on “Tricky” tasks. SAGE Open 2015;5:215824401558461. 10.1177/2158244015584617

[23] Peer E, Brandimarte L, Samat S, et al. Beyond the Turk: alternative platforms for crowdsourcing behavioral research. J Exp Soc Psychol 2017;70:153–63. 10.1016/j.jesp.2017.01.006

[24] Palan S, Schitter C, Prolific SC. Prolific.ac—A subject pool for online experiments. J Behav Exp Finance 2018;17:22–7. 10.1016/j.jbef.2017.12.004

[25] NICE. Ovarian cancer: recognition and initial management. secondary ovarian cancer: recognition and initial management, 2011. Available: https://www.nice.org.uk/guidance/CG122

[26] Richardson DR, Oakes AH, Crossnohere NL, et al. Prioritizing the worries of AML patients: quantifying patient experience using best–worst scaling. Psychooncology 2021;30:1104–11. 10.1002/pon.5652 33544421 PMC10246445

[27] Sawtooth Software, Inc. Maximum difference scaling: improved measures of importance and preference for segmentation. In: Sawtooth software conference proceedings. Fir St., Sequim, WA, 2003.

[28] StataCorp L. Stata statistical software: release 17. College Station, TX: StataCorp LP, 2021: 2021.

[29] Howard K, Salkeld G, Pignone M, et al. Preferences for CT colonography and colonoscopy as diagnostic tests for colorectal cancer: a discrete choice experiment. Value Health 2011;14:1146–52. 10.1016/j.jval.2011.07.012 22152186 PMC3466595

[30] de Bekker-Grob EW, Rose JM, Donkers B, et al. Men's preferences for prostate cancer screening: a discrete choice experiment. Br J Cancer 2013;108:533–41. 10.1038/bjc.2013.5 23361056 PMC3593568

[31] Mansfield C, Ekwueme DU, Tangka FKL. Colorectal cancer screening: preferences, past behavior, and future intentions. Patient 2018:1–13. 10.1007/s40271-018-0308-6 29740804 PMC6226356

[32] Miles A, Taylor SA, Evans REC, et al. Patient preferences for whole-body MRI or conventional staging pathways in lung and colorectal cancer: a discrete choice experiment. Eur Radiol 2019;29:3889–900. 10.1007/s00330-019-06153-4 30937589 PMC6554244

[33] Fitch M, Deane K, Howell D, et al. Women’s experiences with ovarian cancer: reflections on being diagnosed. Can Oncol Nurs J 2003;12:152–9. 10.5737/1181912x123152159 12271917

[34] Jelicic L, Brooker J, Shand L, et al. Experiences and health care preferences of women with ovarian cancer during the diagnosis phase. Psychooncology 2019;28:379–85. 10.1002/pon.4952 30485590

[35] Hol L, de Bekker-Grob EW, van Dam L, et al. Preferences for colorectal cancer screening strategies: a discrete choice experiment. Br J Cancer 2010;102:972–80. 10.1038/sj.bjc.6605566 20197766 PMC2844026

[36] van Dam L, Hol L, de Bekker-Grob EW, et al. What determines individuals' preferences for colorectal cancer screening programmes? a discrete choice experiment. Eur J Cancer 2010;46:150–9. 10.1016/j.ejca.2009.07.014 19683432

[37] Salkeld G, Ryan M, Short L. The veil of experience: do consumers prefer what they know best? Health Econ 2000;9:267–70. 10.1002/(SICI)1099-1050(200004)9:3&lt;267::AID-HEC511&gt;3.0.CO;2-H 10790707

[38] Finn A, Louviere JJ. Determining the appropriate response to evidence of public concern: the case of food safety. Journal of Public Policy & Marketing 1992;11:12–25. 10.1177/074391569201100202

[39] de Bekker-Grob EW, Donkers B, Jonker MF, et al. Sample size requirements for discrete-choice experiments in healthcare: a practical guide. Patient 2015;8:373–84. 10.1007/s40271-015-0118-z 25726010 PMC4575371

[40] Naik G, Ahmed H, Edwards AGK. Communicating risk to patients and the public. Br J Gen Pract 2012;62:213–6. 10.3399/bjgp12X636236 22520906 PMC3310025

[41] Drummond M, Torbica A, Tarricone R. Should health technology assessment be more patient centric? if so, how? Eur J Health Econ 2020;21:1117–20. 10.1007/s10198-020-01182-z 32301000

