# Supplementary material for: Women’s priorities towards ovarian cancer testing: a best–worst scaling study
Source: BMJ Open. 2022 Sep 1;12(9):e061625. doi: 10.1136/bmjopen-2022-061625 (PMC9438192; doi:10.1136/bmjopen-2022-061625)
Supplement: Supplementary data [file bmjopen-2022-061625supp001.pdf]

**Appendix 1: Health-related characteristics of population**

| Characteristic | n (%) |
|---|---|
| **Self-reported overall health** | |
| Very good | 18 (12) |
| Good | 61 (41) |
| Fair | 47 (31) |
| Poor | 14 (9) |
| Very poor | 4 (3) |
| Not reported | 6 (4) |
| **Perceived risk of ovarian cancer** | |
| Very High | 4 (3) |
| High | 12 (8) |
| Average | 83 (55) |
| Low | 33 (22) |
| Very low | 12 (8) |
| Not reported | 6 (4) |
| **Ovarian cancer-related worry** | |
| A great deal | 4 (3) |
| A lot | 25 (17) |
| A moderate amount | 43 (29) |
| A little | 47 (31) |
| Not at all | 26 (17) |
| Not reported | 5 (3) |
| **Personal history of cancer** | 17 (13) |
| **Knew someone who was diagnosed with ovarian cancer** | 25 (17) |
| **Previously tested for ovarian cancer** | 40 (27) |
| **Previously undergone a TVUS (any reason)** | 50 (33) |
| **Cervical screening** | |
| Always attends/attended | 75 (50) |
| Irregularly attends/attended | 37 (25) |
| Never attended/stopped attending | 37 (25) |
| Unknown | 1 (1) |
| **How much confidence and trust do you have in GPs** | |
| A great deal/a lot | 59 (39) |
| A moderate amount | 53 (35) |
| A little | 25 (17) |
| None at all | 2 (1) |
| Unknown | 11 (7) |
| **How much do you feel able to be involved in medical decisions** | |
| A great deal | 12 (8) |
| A lot | 22 (15) |
| A moderate amount | 58 (39) |
| A little | 34 (23) |
| Not at all | 17 (11) |
| Unknown | 7 (5) |
| **How much do you wish to be involved in medical decisions** | |
| A great deal | 60 (40) |
| A lot | 67 (45) |
| A moderate amount | 19 (13) |
| A little | 3 (2) |
| Not at all | 1 (1) |

**Appendix 2: Full summary of raw best-worst scores, counting analysis and conditional logit results**

| Items | Most | Least | Most-Least | Counting | | | Conditional logit | | Ranking |
|---|---|---|---|---|---|---|---|---|---|
| | | | | Standardised score | SD | 95% confidence interval | Coefficient | SE | |
| Sensitivity | 410 | 133 | 277 | 0.308 | 0.60 | 0.21 – 0.40 | 1.44*** | 0.096 | 2 |
| Chance of dying from ovarian cancer | 504 | 162 | 342 | 0.380 | 0.70 | 0.26 – 0.49 | 1.63*** | 0.103 | 1 |
| Choice of appointment time | 122 | 316 | -194 | -0.216 | 0.47 | -0.29 – (-0.15) | 0.08 | 0.085 | 23 |
| Who explains the results | 150 | 306 | -156 | -0.173 | 0.52 | -0.26 – (-0.09) | 0.18* | 0.087 | 20 |
| Pain and discomfort | 246 | 94 | 152 | 0.169 | 0.44 | 0.10 – 0.24 | 1.07*** | 0.084 | 4 |
| Notification of negative test results | 135 | 103 | 32 | 0.036 | 0.34 | -0.02 – 0.09 | 0.72*** | 0.078 | 11 |
| Chance of diagnosing another condition | 289 | 107 | 182 | 0.202 | 0.45 | 0.13 – 0.27 | 1.16*** | 0.088 | 3 |
| Pre-test support | 111 | 147 | -36 | -0.040 | 0.33 | -0.09 – 0.01 | 0.53*** | 0.078 | 15 |
| Test-procedure | 177 | 124 | 53 | 0.059 | 0.34 | 0.00 – 0.11 | 0.79*** | 0.081 | 9 |
| Staff attitude | 120 | 128 | -8 | -0.009 | 0.34 | -0.06 – 0.05 | 0.60*** | 0.077 | 14 |
| Post-test support | 106 | 84 | 22 | 0.024 | 0.28 | -0.02 – 0.07 | 0.70*** | 0.076 | 13 |
| Time away from usual activities | 138 | 358 | -220 | -0.244 | 0.56 | -0.33 – (-0.15) | Ref | Ref | 25 |
| Specificity | 239 | 125 | 114 | 0.127 | 0.42 | 0.06 – 0.19 | 0.95*** | 0.085 | 5 |
| Travel time | 107 | 292 | -185 | -0.206 | 0.44 | -0.28 – (-0.14) | 0.11 | 0.081 | 21 |
| Time to notification of test results | 194 | 112 | 82 | 0.091 | 0.37 | 0.03 – 0.15 | 0.87*** | 0.082 | 8 |
| Openness of healthcare providers | 134 | 101 | 33 | 0.037 | 0.32 | -0.01 – 0.09 | 0.72*** | 0.78 | 10 |
| Number of follow up tests | 129 | 99 | 30 | 0.033 | 0.25 | -0.00 – 0.07 | 0.71*** | 0.078 | 12 |
| Chance of an inconclusive result | 174 | 74 | 100 | 0.111 | 0.31 | 0.06 – 0.16 | 0.92*** | 0.077 | 6 |
| Out-of-pocket costs | 154 | 229 | -75 | -0.083 | 0.48 | -0.16 – (-0.01) | 0.41*** | 0.083 | 18 |
| Gender of healthcare provider | 182 | 401 | -219 | -0.243 | 0.67 | -0.35 – (-0.14) | 0.001 | 0.091 | 24 |
| How test results are returned | 104 | 290 | -186 | -0.207 | 0.40 | -0.27 – (-0.14) | 0.11 | 0.082 | 22 |
| Test location | 132 | 264 | -132 | -0.147 | 0.46 | -0.22 – (-0.07) | 0.25*** | 0.083 | 19 |
| Test duration | 95 | 161 | -66 | -0.073 | 0.32 | -0.12 – (-0.02) | 0.44*** | 0.077 | 17 |
| Information included with the invitation | 135 | 177 | -42 | -0.047 | 0.35 | -0.10 – 0.01 | 0.51*** | 0.081 | 16 |
| Waiting time for the test | 213 | 113 | 100 | 0.111 | 0.37 | 0.05 – 0.17 | 0.91*** | 0.084 | 6 |

Key: ■ Five best scoring attributes in each round, ■ Five worst scoring attributes in each round. For the "least" column best and worst are inversed so worst scoring = rated least important most frequently

**Appendix 3: Distribution of B-W scores across individuals. Demonstrating the degree of heterogeneity in preferences across individuals. Each attribute appeared within the survey six times meaning scores could range from 6 (always selected as most important) to -6 (always selected as least important).**

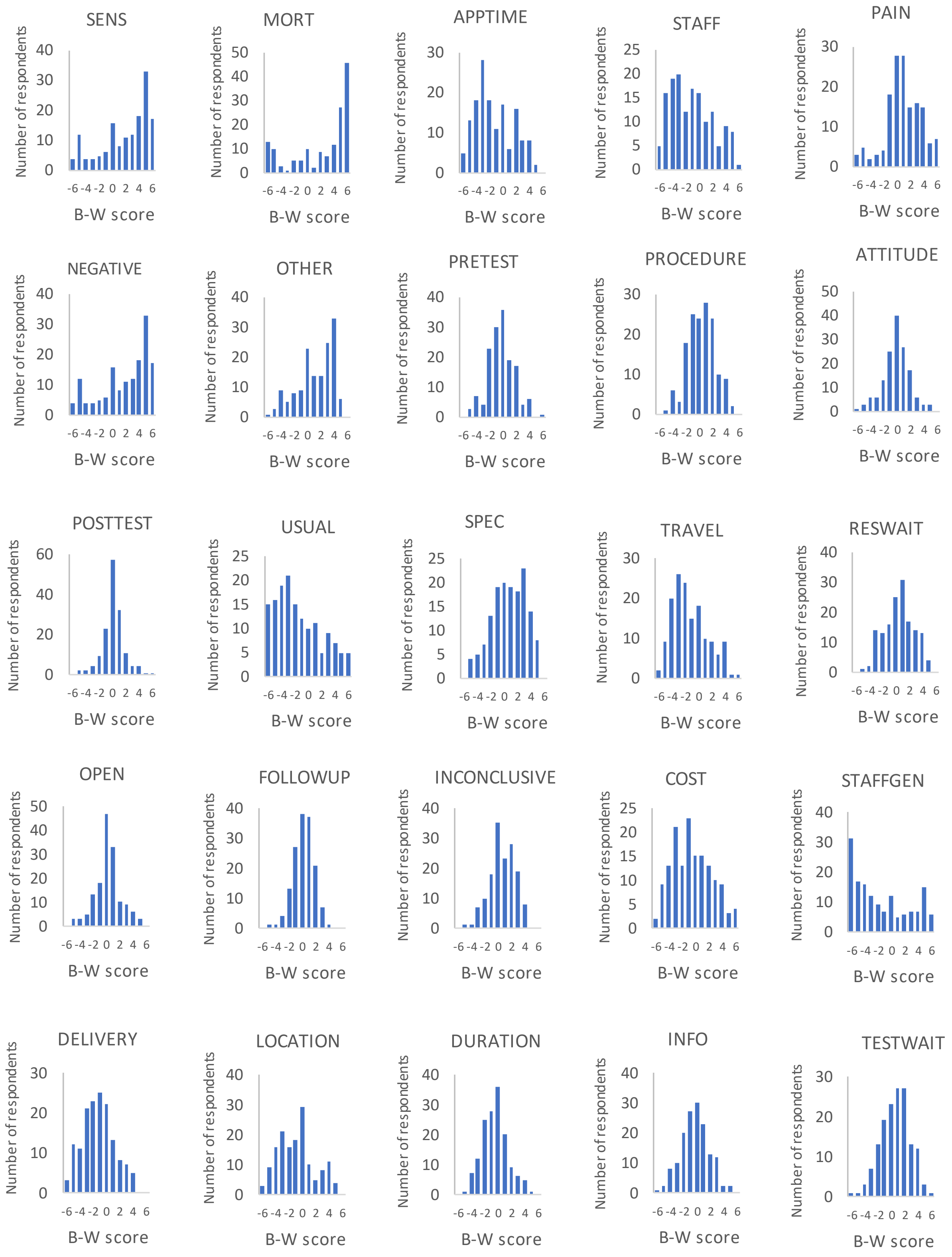

**Appendix 4: Comparison of raw counting estimates and conditional logit estimates. Results were highly correlated**

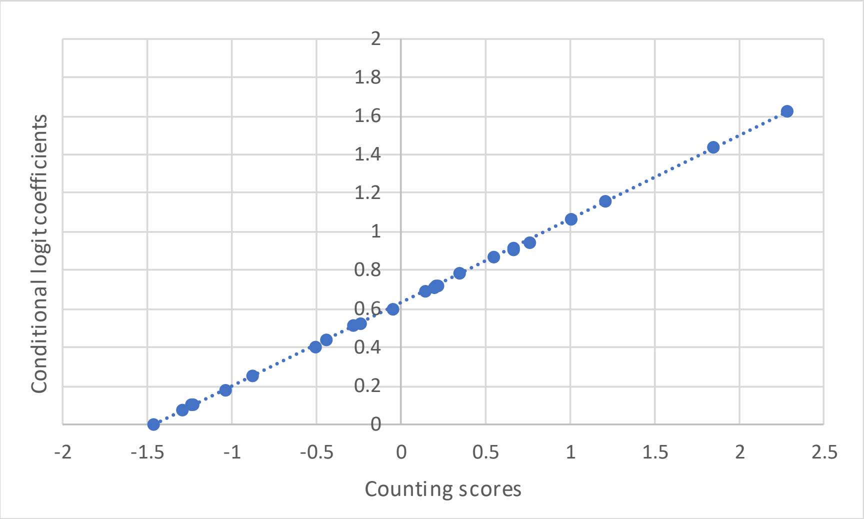

**Appendix 5: Heteroscedastic logit estimates used to investigate differences in scale (i.e. error variance) between subgroups. A dummy variable representing previous test experience (either TVUS or OC testing) was included as an explanatory factor of the scale parameter. In both instances the scale term was not significant indicating observed differences in priorities between subgroups were not attributable to differences in error variance.**

| | OC test experience | | TVUS experience | |
|---|---|---|---|---|
| | Coefficient | SE | Coefficient | SE |
| Items | | | | |
| Sensitivity | 1.63*** | 0.28 | 1.26*** | 0.35 |
| Chance of dying from ovarian cancer | 1.86*** | 0.30 | 1.47*** | 0.37 |
| Choice of appointment time | 0.13 | 0.17 | 0.07 | 0.14 |
| Who explains the results | 0.39* | 0.21 | 0.29* | 0.17 |
| Pain and discomfort | 1.13*** | 0.26 | 0.87*** | 0.27 |
| Notification of negative test results | 0.84*** | 0.19 | 0.68*** | 0.21 |
| Chance of diagnosing another condition | 1.30*** | 0.25 | 1.08*** | 0.26 |
| Pre-test support | 0.73*** | 0.18 | 0.58*** | 0.18 |
| Test-procedure | 1.06*** | 0.23 | 0.87*** | 0.24 |
| Staff attitude | 0.64*** | 0.20 | 0.56*** | 0.17 |
| Post-test support | 0.82*** | 0.19 | 0.68*** | 0.19 |
| Time away from usual activities | Ref | Ref | Ref | Ref |
| Specificity | 1.15*** | 0.24 | 0.22*** | 0.14 |
| Travel time | 0.23 | 0.19 | 0.86 | 0.23 |
| Time to notification of test results | 1.05*** | 0.22 | 0.64*** | 0.19 |
| Openness of healthcare providers | 0.74*** | 0.22 | 0.83*** | 0.22 |
| Number of follow up tests | 1.04*** | 0.21 | 0.85*** | 0.21 |
| Chance of an inconclusive result | 1.00*** | 0.21 | 0.41*** | 0.14 |
| Out-of-pocket costs | 0.44** | 0.17 | 0.17*** | 0.18 |
| Gender of healthcare provider | 0.27 | 0.23 | 0.19 | 0.14 |
| How test results are returned | 0.21 | 0.20 | 0.15 | 0.14 |
| Test location | 0.18 | 0.18 | 0.26 | 0.15 |
| Test duration | 0.34** | 0.16 | 0.44* | 0.18 |
| Information included with the invitation | 0.58*** | 0.20 | 0.86** | 0.22 |
| Waiting time for the test | 1.06*** | 0.21 | 0.22*** | 0.14 |
| Scale factor | | | | |
| Previous OC test (1=yes, 0=no) | -0.07 | 0.36 | | |
| Previous TVUS (1=yes, 0-no) | | | 0.54* | 0.32 |
| Model statistics | | | | |
| Log-likelihood | -3309.6 | | | |
| Observations | 31080 | | 31080 | |
| N | 150 | | 150 | |

Confidence levels: ***99%, ** 95%, *90%
