# Supplementary material for: Women’s priorities towards ovarian cancer testing: a best–worst scaling study
Source: BMJ Open. 2022 Sep 1;12(9):e061625. doi: 10.1136/bmjopen-2022-061625 (PMC9438192; doi:10.1136/bmjopen-2022-061625)
Supplement: Author's manuscript [file bmjopen-2022-061625.draft_revisions.pdf]

BMJ Open is committed to open peer review. As part of this commitment we make the peer review history of every article we publish publicly available.

When an article is published we post the peer reviewers' comments and the authors' responses online. We also post the versions of the paper that were used during peer review. These are the versions that the peer review comments apply to.

The versions of the paper that follow are the versions that were submitted during the peer review process. They are not the versions of record or the final published versions. They should not be cited or distributed as the published version of this manuscript.

BMJ Open is an open access journal and the full, final, typeset and author-corrected version of record of the manuscript is available on our site with no access controls, subscription charges or pay-per-view fees (http://bmjopen.bmj.com).

If you have any questions on BMJ Open's open peer review process please email info.bmjopen@bmj.com

BMJ Open

Women's priorities towards ovarian cancer testing: A best-worst scaling study

Journal:	*BMJ Open*
Manuscript ID	bmjopen-2022-061625
Article Type:	Original research
Date Submitted by the Author:	01-Feb-2022
Complete List of Authors:	Hall, Rebekah; University of Exeter, Health Economics Group<br>Medina-Lara, Antonieta; University of Exeter Medical School, Health Economics Group<br>Hamilton, Willie; University of Exeter, Primary Care Diagnostics<br>Spencer, Anne; University of Exeter Medical School, Health Economics
Keywords:	Gynaecological oncology < GYNAECOLOGY, Health economics < HEALTH SERVICES ADMINISTRATION & MANAGEMENT, Gynaecological oncology < ONCOLOGY, Ultrasound < RADIOLOGY & IMAGING

SCHOLARONE™
Manuscripts

I, the Submitting Author has the right to grant and does grant on behalf of all authors of the Work (as defined in the below author licence), an exclusive licence and/or a non-exclusive licence for contributions from authors who are: i) UK Crown employees; ii) where BMJ has agreed a CC-BY licence shall apply, and/or iii) in accordance with the terms applicable for US Federal Government officers or employees acting as part of their official duties; on a worldwide, perpetual, irrevocable, royalty-free basis to BMJ Publishing Group Ltd ("BMJ") its licensees and where the relevant Journal is co-owned by BMJ to the co-owners of the Journal, to publish the Work in this journal and any other BMJ products and to exploit all rights, as set out in our licence.

The Submitting Author accepts and understands that any supply made under these terms is made by BMJ to the Submitting Author unless you are acting as an employee on behalf of your employer or a postgraduate student of an affiliated institution which is paying any applicable article publishing charge ("APC") for Open Access articles. Where the Submitting Author wishes to make the Work available on an Open Access basis (and intends to pay the relevant APC), the terms of reuse of such Open Access shall be governed by a Creative Commons licence – details of these licences and which Creative Commons licence will apply to this Work are set out in our licence referred to above.

Other than as permitted in any relevant BMJ Author's Self Archiving Policies, I confirm this Work has not been accepted for publication elsewhere, is not being considered for publication elsewhere and does not duplicate material already published. I confirm all authors consent to publication of this Work and authorise the granting of this licence.

For peer review only

Title: Women's priorities towards ovarian cancer testing: A best-worst scaling study

Rebekah Hall[a], Antonieta Medina-Lara[a], Willie Hamilton[b], Anne E Spencer[a]

[a] Health Economics Group, College of Medicine and Health, University of Exeter, Exeter, UK

[b] Primary Care Diagnostics, College of Medicine and Health, University of Exeter, Exeter, UK

Corresponding Author:

Rebekah Hall

Address: University of Exeter, St Luke's Campus, Heavitree, Exeter, EX1 2LU, UK

Email: rh591@exeter.ac.uk

Phone: +44 (0)1392 725565

ORCID ID:

Rebekah Hall: 0000-0003-1804-993X

Antonieta Medina-Lara: 0000-0001-7325-8246

Willie Hamilton: 0000-0003-1611-1373

Anne Spencer: 0000-0002-8163-3103

Word counts: Abstract: 262 Main body: 3305

Abstract:

Objective: To investigate the importance of key characteristics relating to diagnostic testing for ovarian cancer and to understand how previous test experience influences priorities.

Design: Object-case best-worst scaling embedded in an online survey

Setting: Primary care diagnostic testing in England and Wales

For peer review only

For peer review only - http://bmjopen.bmj.com/site/about/guidelines.xhtml

Participants: 150 women with ovaries over 40 years old living in England and Wales.

Methods: We used best-worst scaling, a preference-based survey method, to elicit the relative importance of twenty-five characteristics relating to ovarian cancer testing following a systematic review. Analysis used the best-minus-worst score method. Sub-group analysis investigated variations based on testing history.

Main outcome measures: Relative importance scores

Results: “Chance of dying from ovarian cancer” (0.380, CI[0.26–0.49]) was the most important factor to respondents, closely followed by “test sensitivity” (0.308, CI[0.21–0.40]). In contrast, “time away from usual activities” (-0.244, CI[-0.33–(-0.15)]) and “gender of healthcare provider” (-0.243, CI[-0.35–(-0.14)]) were least important to respondents overall. Women who had previously undergone testing placed higher importance on certain characteristics including “openness of healthcare providers” and “chance of diagnosing another condition” at the expense of reduced emphasis on characteristics such as “pain and discomfort” and “time away from usual activities”.

Conclusions: The results clearly demonstrated attributes at the extreme, that were most and least important to women considering ovarian cancer testing. Differences in preferences by testing history demonstrate an experience effect, whereby preferences adapt over time based on evidence and experience. Acknowledging these differences helps to identify underlying barriers and facilitators for women with no test experience as well as shortcomings of current service based on women with experience.

Strengths and limitations:

- This study adds to a very limited evidence base of studies assessing preferences towards diagnostic testing for cancer and specifically ovarian cancer.
- Attribute selection within this study is based on a rigorous, published systematic review and utilised patient and public involvement to ensure the relevance to the target population

For peer review only - http://bmjopen.bmj.com/site/about/guidelines.xhtml

- Object-case best worst scaling is ideal for establishing the relative importance of a large number of characteristics and has been proven to be easier to complete and more effective than alternative methods such as ranking or ratings tasks
- A key limitation of the study relates to the representativeness of the sample. Due to the recruitment method, the sample is not fully representative of the population in key demographics including ethnicity and age distribution
- The hypothetical nature of the screnarios is also a potential limitation. Task choices and preferences of individuals when/if actually offered testing may differ, particularly for those who have not undergone testing previously

1. Introduction

Ovarian cancer is the seventh most common cancer in women worldwide, with over 200,000 new cases annually.[1] Earlier diagnosis is an ongoing challenge globally.[2] For instance, in the UK, almost 60% of cases are diagnosed at a late stage where five-year survival is just 13.4% for those diagnosed at stage IV.[3]

National guidelines for the investigation of suspected ovarian cancer in symptomatic women vary substantially between countries.[4] Existing tests include the CA125 blood test and imaging tests, most commonly a transabdominal/transvaginal ultrasound (TVUS) but also Computed Tomography (CT) and Magnetic Resonance Imaging (MRI).[5] Variations in guidance represents uncertainty around the accuracy of existing test strategies, especially for the early investigation of symptoms. Evidence evaluating the performance of diagnostic tests in a primary care setting is very limited.[6] Furthermore, for recommendations to be effective, clinical guidelines must also consider the preferences of those offered testing, particularly in healthcare systems emphasising shared-decision making.[7] Available tests differ substantially, not only in terms of performance but also service delivery and patient experience. Aspects of tests may be prioritised differently; for instance, patients may accept a lower

For peer review only - http://bmjopen.bmj.com/site/about/guidelines.xhtml

levels of accuracy a less invasive test. Thus, it may be necessary to weight characteristics differently when considering the overall balance of benefits and harms.

To date, very little attention has been paid to the preferences of women facing ovarian cancer testing, particularly in a diagnostic setting. Existing studies usually focus on screening trials[8], single test modalities[9] or single aspects of acceptability, such as pain.[10] To address the current evidence gap, we aimed to elicit preferences for twenty-five key characteristics ('attributes') of ovarian cancer testing, using best-worst scaling (BWS). This is a stated preference technique that has been demonstrated to provide higher predictive values than ranking or rating methods whilst being less cognitively demanding.[11] Understanding the importance of key attributes allows such characteristics to be given greater salience in future decisions about testing, particularly in guideline revision.

Previous studies have demonstrated preferences may differ based on previous experience of the health event. We therefore examined the relationship between preferences around ovarian cancer testing and test experience.[12 13]

2. Methods

2.1 Best worst scaling

We used object case best-worst scaling (BWS) method to identify women's preferences for attributes associated with diagnostic testing for ovarian cancer. This type of BWS aims to assess the relative importance of, or preference for, attributes based on the underlying principles of random utility theory[14]. During BWS tasks, participants respond to choice tasks presenting a subset of attributes and asked to select the "best" or "most important" and "worst" or "least important" attribute. Simultaneously examining attributes selected as "most" and "least" important provides greater information than examining the most important attribute alone, allowing the underlying preference scale to be inferred.

2.2 Experimental design

For peer review only - http://bmjopen.bmj.com/site/about/guidelines.xhtml

Results demonstrate the importance of an attribute, relative to the other included attributes. It is therefore important to use a rigorous attribute selection process to ensure the most salient attributes are included in the experiment. We performed a systematic review of the preference-based literature to identify attributes relating to cancer testing.[15] Potential attributes were then narrowed down by the authors based on relevance to ovarian cancer testing in symptomatic patients using an iterative Delphi method process. In total, twenty-five attributes were selected for inclusion (Table 1).

Table 1: Attributes included in the BWS study

	Attribute and definition
1	**Test sensitivity**<br>*Chance that the test will miss cancer in a patient who actually does have the disease (false-negative result)*
2	**Chance of dying from ovarian cancer**<br>*How much having the test decreases the chance of dying from ovarian cancer*
3	**Choice of appointment time**<br>*Whether a person can choose an appointment time or if the appointment time is assigned by the health care provider*
4	**Who explains the results**<br>*Type of health care provider who explains the test results e.g. Nurse, doctor etc.*
5	**Pain and discomfort**<br>*The level of pain and/or discomfort experienced during and after the test*
6	**Notification of negative test results**<br>*Whether you are contacted if your results are normal*
7	**Chance of diagnosing another condition**<br>*If symptoms are not caused by cancer, the chance the test can identify other reason for the symptoms*
8	**Pre-test support**<br>*Level of support received before having the test describing what will happen during the test and what the results might show*
9	**Test procedure**<br>*What having the test will involve. For ovarian cancer this could be a blood test or an transvaginal ultrasound (internal ultrasound of the reproductive organs)*
10	**Staff attitude**<br>*How the health care provider treats you while conducting the test*
11	**Post-test support**<br>*Level of support received after getting the results of the test relating to the meaning of your results and what will happen next*
12	**Time away from usual activities**<br>*The total amount of time spent having the test instead of doing your usual daily activities*
13	**Test specificity**<br>*Chance of unnecessary further invasive testing (false-positive result)*
14	**Travel time**<br>*The total amount of time spent travelling to and from the test*
15	**Time to notification of results**

For peer review only - http://bmjopen.bmj.com/site/about/guidelines.xhtml

For peer review only

	The length of time it takes to hear the results after having the test
16	**Openness of health care providers**<br>*How open health care providers are with their thoughts about the cause of your symptoms and the tests they recommend*
17	**Number of follow up tests**<br>*How many additional tests are needed to confirm a diagnosis*
18	**Chance of an inconclusive result**<br>*The chance the results are unclear and the test would need to be repeated after a waiting period*
19	**Out of pocket costs**<br>*How much it will personally cost a person to have the test e.g. Travel costs, childcare costs, time off work etc. The cost of the test is covered by the NHS*
20	**Gender of healthcare provider**<br>*Gender of the staff member giving you the test*
21	**How test results are returned**<br>*e.g. in person, phone, letter*
22	**Test location**<br>*Where the test takes place*
23	**Test duration**<br>*The length of time spent having the test*
24	**Information included with the invitation**<br>*The level and type of information received about the test*
25	**Waiting time for the test**<br>*How long a person has to wait to have the test after being referred by their GP*

A balanced incomplete block design for the 25 attributes was constructed using SAS 9.4. A final design (d-efficiency of 83.3%) consisting of 30 choice tasks each with 5 attributes was selected based on a trade-off between survey complexity and design efficiency. Each attribute appeared six times across the choice tasks. The position of the attributes within choice tasks was optimised such that attributes were listed in 1st-5th position equally. The order of choice tasks was randomised across participants. Each participant completed all thirty choice tasks. An example of a choice task is shown in Figure 1.

2.3 Questionnaire

The questionnaire was developed and collected using LimeSurvey, an online survey platform. The survey consisted of four stages:

i. Socio-demographic questions e.g. age, education, employment status
ii. Best-worst scaling questions (including a warm-up task)
iii. Task feedback questions e.g. task difficulty

For peer review only

iv. Background questions relating to health-related characteristics e.g. self-reported health, testing history, current and desired in medical decisions

Question framing and answer categories for stages i and iii-iv were copied or adapted from established national surveys. Early piloting with five women suggested the survey would take 30 minutes to complete. Given the length of the survey and online administration method, three attention checks following the instructional manipulation format were embedded (e.g. "Select 'very important' to indicate you are paying attention").[16] Respondents who failed all three attention checks were removed from the analysis. A full version of the questionnaire is available upon request.

Patient and public involvement: The survey was shared and discussed with the Cancer Research UK funded CanTest PPI lead, Margaret Johnson.

2.4 Participants

A sample size of 150 women was estimated to be required. Participants were recruited via Prolific, an online platform for researchers conducting social science experiments. Our study focused on the priorities of women over 40 (no upper limit), the group most likely to be offered testing.[17] Participation was also limited to those living in England and Wales: no other limitations were applied. The study was approved by the University of Exeter UEBS-REC (eUEBS003725).

2.5 Analysis

Analysis was performed based on the MaxDiff approach,[18] which assumes individuals select the pair of attributes with the greatest difference in terms of importance according to their personal preferences by implicitly comparing all possible pairs within a given choice set.

Importance scores for each attribute were calculated using the counting approach; whereby the number of times an attribute was picked as 'least important' is subtracted from the number of times it was chosen as 'most important'. Each attribute appeared 6 times across all choice tasks, meaning

For peer review only - http://bmjopen.bmj.com/site/about/guidelines.xhtml

best-worst scores could range from -6 to +6 at the individual level. Individual scores were aggregated and standardised to calculate an overall mean score for the sample ranging from -1 to +1[1].

Importance scores represent the relative importance of each attribute. A score close to +1 indicates an attribute is highly influential, whereas attributes with scores near to -1 demonstrate much less relevance. This approach provides an easily calculable and interpretable results: scores have been demonstrated to provide similar results to coefficients from logit models.[14 18 19]

2.5.1 Subgroup analysis

Subgroup analysis was performed to explore the relationship between previous testing and preferences for future testing. Experience was captured by two questions that asked whether women had previously: 1) undergone testing for ovarian cancer or 2) received a transvaginal ultrasound for any reason. Differences in importance score between groups were tested using Mann-Whitney U tests.

3 Results

In total 159 women responded to the survey. The average response time for the questionnaire was 29 mins 51 seconds. Four submissions were incomplete, two were removed due to failing all attention checks, and a further three responses were removed due to incorrect completion of the best-worst section of the survey, resulting in 150 responses for the final analysis. Respondents varied substantially in how difficult they found the best-worst portion of the questionnaire with 42% (63/150) reporting it easy/very easy but 38% (57/150) finding it difficult/very difficult. There were no significant differences in BWS responses between those who found the task difficult versus those who did not.

3.1 Demographics

[1]Scores were standardised using the following equation: population-level best-worst score/(number of times attribute appeared x total sample size).

For peer review only - http://bmjopen.bmj.com/site/about/guidelines.xhtml

The demographics of the sample are presented in Table 2. The age ranged from 40 to 87 years old with a mean of 51.4 (SD=9.1). Most participants were white (120/150; 80%), married/in a relationship (97/150; 65%) and employed (78/150; 52%).

Most women perceived their risk of cancer as low-average (128/150; 85%) and ovarian cancer-related anxiety was generally low-moderate amongst respondents (116/150; 77%). Overall, fifty women (33%) reported previously undergoing a TVUS for any reason. Forty (27%) women reported being previously tested for ovarian cancer, with CA125 blood test being the most common test.

Crucially, when asked, 127/150 women (89%) stated they wished for a great deal/a lot of involvement in decisions relation to their own care but only 34/150 (23%) currently felt this was achieved, with 17/150 (11%) respondents felt unable to be involved in medical decisions at all. Further details are found in Appendix 1.

Table 2: Descriptive characteristics: sociodemographic

Characteristic	n (%)
**Age**	
Mean (SD)	51.4 (9)
Range	40-87
**Ethnicity**	
White	120 (80)
Asian	8 (5)
Black	3 (2)
Mixed	3 (2)
Other	9 (6)
Not reported	7 (4)
**Number of children, mean (SD)**	1.3 (1)
**Relationship status**	
Married	75 (50)
In a relationship	22 (15)

For peer review only - http://bmjopen.bmj.com/site/about/guidelines.xhtml

Single	19 (13)
Divorced/separated	26 (17)
Widowed	6 (4)
Not reported	2 (1)
**Level of education**	
GCSE	37 (25)
A-Level/ College	25 (17)
Undergraduate	41 (27)
Post-graduate	35 (23)
No qualifications	1 (1)
Other	9 (6)
Not reported	2 (1)
**Employment status**	
Full-time	47 (31)
Part-time	32 (21)
Self-employed	23 (15)
Not employed	11 (7)
Retired	14 (9)
Other	18 (12)
Not reported	5 (3)

For peer review only

3.2 Best-worst results

BWS results are presented in Figure 2. Scores were bound between -1 and 1. Scores tending toward the extremes of the scale would imply homogeneity across respondents and consistency between responses across questions on an individual level. Importance scores ranged from -0.224 to 0.380, suggesting high levels of heterogeneity in preferences regarding test characteristics across respondents (see Appendix 2).

For peer review only - http://bmjopen.bmj.com/site/about/guidelines.xhtml

Overall, "*Chance of dying from ovarian cancer*" (0.380, CI[0.26–0.49]) was most important to women when considering ovarian cancer testing, followed by "*Test sensitivity*" (0.308, CI[0.21–0.40]). Conversely, "*Time away from usual activities*" (-0.244, CI[-0.33–(-0.15)]) and "*Gender of healthcare provider*" (-0.243, CI[-0.35–(-0.14)]) were considered least important to women when facing diagnostic testing and were statistically indistinguishable from each other. See Appendix 3 for further details.

The most important attributes to respondents were clear and distinct: however, spatial visualisation of results (Figure 2) demonstrated clustering towards the centre and bottom of the scale. Clusters are distinct to other attributes but preferences between attributes within clusters is less distinguishable. Cluster 1 consisted of attributes that were considered important (indicated by positive importance scores) but not most important, whereas cluster 2 consisted of attributes that respondents appeared to be indifferent towards as indicated by low levels of selection in both "most" and "least" important domains. Finally, cluster 3 includes attributes that were considered similarly less important to respondents.

3.2.1 Subgroup analysis

Women previously tested for ovarian cancer valued "*openness of healthcare provider*" significantly higher than test-naïve individuals whereas "*time away from usual activities*" became even less important to decision-making. There was some evidence that "*pain and discomfort*" and "*test specificity*" were less important to previously tested women whereas "*how test results are returned"* had increased importance but remained amongst the lowest ranked attributes overall (Figure 3).

For women who had previously undergone a TVUS, "choice of appointment time" and "*information included with the invitation*" were significantly less important whereas "*chance of diagnosing another condition*" and "*out-of-pocket costs*" were significantly more important (Figure 4).

4 Discussion

For peer review only - http://bmjopen.bmj.com/site/about/guidelines.xhtml

4.1 Summary of main findings

This is the first study to investigate the priorities of women relating to diagnostic testing for ovarian cancer. The results of this study highlight the importance yet current neglect of incorporating the preferences of patients into medical testing decisions. When asked, almost 90% (134/150) of respondents wanted heavy involvement in medical decisions: however, less than a quarter of women currently felt able to do so.

The results are particularly useful in identifying attributes at either extreme of the scale, clearly demonstrating the attributes that are most and least important to women considering ovarian cancer. Low concurrence between attributes and high levels of heterogeneity meant discrimination between mid-range attributes was less clear. Overall, "*chance of dying from ovarian cancer*", "*test sensitivity*" and "*chance of diagnosing another condition*" were the most important attributes when considering testing for ovarian cancer. Oppositely, "*time away from usual activities*" and "*gender of healthcare provider*" were the least important factors. The priorities of previously tested women were generally similar to women who had never undergone testing, but there were a few key differences between these two groups suggesting experience is an important determinant of preferences.

4.2 Results in the Context of Published Literature

The prioritisation of outcome and test-specific attributes and the relative lack of importance placed on service delivery attributes is reflective of findings from the wider literature examining preferences towards cancer screening and diagnosis.[15 20-23]

The relative importance of "*openness of healthcare providers"* is echoed by qualitative research exploring the diagnostic experiences of women with ovarian cancer where shortcomings in doctor-patient communication was a recurring theme. In particular, patients raised concerns about doctors' willingness and ability to openly communicate and share information.[24 25] Decreased importance of particular attributes to tested women may suggest these attributes of testing are already being

For peer review only - http://bmjopen.bmj.com/site/about/guidelines.xhtml

achieved/acceptable within the current system. For example, the decreased importance of "*pain and discomfort*" for both women who have been tested for ovarian cancer and women who have previously received an ultrasound (NS) is expected given the finding that almost 80% of women who had previously undergone a transvaginal ultrasound experienced little to no pain.[10]

Existing studies examining differences in preferences towards cancer testing between previously tested and untested individuals have typically focused on colorectal cancer screening, but also found evidence of statistically different preference between the two groups.[26 27] Hol, et al. [26] found previously screened individuals displayed stronger and more positive preferences towards different screening modalities and shorter screening intervals than unscreened counterparts. In a similar study, van Dam, et al. [27] found limited differences between people with and without screening experience (mortality risk reduction only), however, preferences across previously screened individuals differed significantly based on the particular screening modality received. These results are evidence of an experience effect, via "status quo bias", where individuals place a higher value on goods or services that are more familiar.[28]

4.3 Strengths and Limitations

Best-worst scaling provides a straightforward method for capturing the priorities of women. The method is preferable to ranking/rating tasks due to its ability to measure the importance of large numbers of attributes whilst limiting complexity and cognitive burden.[19]

The survey length and high number of attributes represent a potential limitation. In total, 38% of respondents reported the task as difficult/very difficult. Despite this, drop-out rates were low (3% (4/150) of people began the survey but did not complete) and there were no differences between responses according to reported task difficulty.

For peer review only - http://bmjopen.bmj.com/site/about/guidelines.xhtml

As with all choice-based studies measuring stated preferences, establishing external validity is a challenge. Choices made within this study were hypothetical and may differ from the choices and preferences of individuals actually offered testing.

4.4 Implications for Practice and Future Research

The findings of this study offer useful insights into potential barriers and facilitators of undergoing testing in a timely manner. Interestingly, attributes involving an element of risk or uncertainty dominated the top-ranking positions. However, how to best explain complex aspects of test performance such as sensitivity and specificity to patients is a clear challenge.[29]

Differences between the preferences of women with and without test experience suggest that preferences are continuously adapted based on evidence and experience gained over time. When considering policy decisions, it is therefore important to carefully consider whose preferences should be prioritised - a longstanding debate within the field of health technology assessment.[30] Arguably, it is important to consider both perspectives; preferences of women with test experience help to identify unmet or inadequate aspects of current service provision, whereas preconceived preferences of testing-naïve women may reveal underlying barriers and facilitators of testing since the initial decision to undergo testing is based on these pre-existing preferences. In both instances, mismatches in preferences and practice may lead to delays in seeking help or testing for future symptoms.

High levels of heterogeneity between individuals highlight the importance of a personalised approach to patient interactions throughout the diagnostic process. Patients are likely to have different concerns and priorities during this time, however, the importance of patient-input and shared decision-making appears to be less prioritised in diagnostic settings compared to decisions regarding cancer screening and subsequent treatment where preferences have been studied more extensively.

5. Conclusion

For peer review only - http://bmjopen.bmj.com/site/about/guidelines.xhtml

Preferences towards diagnostic testing have been underexplored to date. Understanding what matters most to patients may improve reduce anxiety around testing, facilitate earlier help-seeking behaviour and improve patient satisfaction. This study highlights that test sensitivity and mortality impacts are the most important factors to patients facing ovarian cancer testing. However, results varied significantly across individuals demonstrating the need for an individualised approach to consultations regarding diagnostic care. Our results can help inform policies and diagnostic guidelines designed to encourage earlier help-seeking behaviour as well as help to evaluate the patient-friendliness of emerging test strategies for suspected ovarian cancer.

Conflicts of interest: No conflicts of interest

Funding statement: This research arises from the CanTest Collaborative, which is funded by Cancer Research UK (reference number: C8640/A23385), of which Rebekah Hall is a funded PhD student, Willie Hamilton is director and Anne E Spencer is a senior faculty member.

Ethical approval: This study was approved by the University of Exeter UEBS-REC in July 2020. Reference number: eUEBS003725.

Author contributions: All authors contributed to the study conception and design. RH and AM-L conducted data analysis. RH prepared the first draft of the manuscript and AS, AM-L and WH edited and approved the final version.

Acknowledgements: The authors thank Margaret Johnson, CanTest PPI Panel lead, for providing feedback during the development of this study.

Data sharing statement: Anonymised survey data and analysis code are available from the corresponding over upon reasonable request.

References

For peer review only - http://bmjopen.bmj.com/site/about/guidelines.xhtml

1. Reid BM, Permuth JB, Sellers TA. Epidemiology of ovarian cancer: a review. *Cancer biology & medicine* 2017;14(1):9.
2. Bhatla N, Jones A. THE WORLD OVARIAN CANCER COALITION ATLAS. 2018
3. Cancer Research UK. Ovarian Cancer Statistics 2019 [Available from: https://www.cancerresearchuk.org/health-professional/cancer-statistics/statistics-by-cancer-type/ovarian-cancer#heading-Two.
4. Funston G, Van Melle M, Baun M-LL, et al. Variation in the initial assessment and investigation for ovarian cancer in symptomatic women: a systematic review of international guidelines. *BMC cancer* 2019;19(1):1028.
5. NHS UK. Diagnosis: Ovarian Cancer 2020 [Available from: https://www.nhs.uk/conditions/ovarian-cancer/diagnosis/.
6. Sundar S, Neal RD, Kehoe S. Diagnosis of ovarian cancer. *Bmj* 2015;351:h4443.
7. NICE. The guidelines manual: Process and methods [PMG6] 2012 [Available from: https://www.nice.org.uk/process/pmg6/resources/how-nice-clinical-guidelines-are-developed-an-overview-for-stakeholders-the-public-and-the-nhs-2549708893/chapter/nice-clinical-guidelines.
8. Drescher CW, Nelson J, Peacock S, et al. Compliance of average-and intermediate-risk women to semiannual ovarian cancer screening. *Cancer Epidemiology and Prevention Biomarkers* 2004;13(4):600-06.
9. Gentry-Maharaj A, Sharma A, Burnell M, et al. Acceptance of transvaginal sonography by postmenopausal women participating in the United Kingdom Collaborative Trial of Ovarian Cancer Screening. *Ultrasound in obstetrics & gynecology* 2013;41(1):73-79.
10. Cowan Bennett C, Richards D. Patient acceptance of endovaginal ultrasound. *Ultrasound in Obstetrics and Gynecology* 2000;15(1):52-55.
11. Hollis G, Westbury C. When is best-worst best? A comparison of best-worst scaling, numeric estimation, and rating scales for collection of semantic norms. *Behavior research methods* 2018;50(1):115-33.
12. Czajkowski M, Hanley N, LaRiviere J. The effects of experience on preferences: theory and empirics for environmental public goods. *American Journal of Agricultural Economics* 2015;97(1):333-51.
13. Neuman T, Neuman E, Neuman S. Explorations of the effect of experience on preferences for a health-care service. *The Journal of Socio-Economics* 2010;39(3):407-19.
14. Flynn TN, Louviere JJ, Peters TJ, et al. Best–worst scaling: what it can do for health care research and how to do it. *Journal of health economics* 2007;26(1):171-89.
15. Hall R, Medina-Lara A, Hamilton W, et al. Attributes Used for Cancer Screening Discrete Choice Experiments: A Systematic Review. *The Patient-Patient-Centered Outcomes Research* 2021:1-17.
16. Hauser DJ, Schwarz N. It'sa trap! Instructional manipulation checks prompt systematic thinking on "tricky" tasks. *Sage Open* 2015;5(2):2158244015584617.
17. NICE. Ovarian cancer: recognition and initial management 2011 [Available from: https://www.nice.org.uk/guidance/CG122.
18. Marley A, Flynn TN, Australia V. Best worst scaling: theory and practice. *Handbook of Choice Modelling Edward Elgar Publishing, Leeds (UK)* 2012
19. Finn A, Louviere JJ. Determining the appropriate response to evidence of public concern: the case of food safety. *Journal of Public Policy & Marketing* 1992;11(2):12-25.
20. Howard K, Salkeld G, Pignone M, et al. Preferences for CT colonography and colonoscopy as diagnostic tests for colorectal cancer: A discrete choice experiment. *Value in Health* 2011;14(8):1146-52. doi: http://dx.doi.org/10.1016/j.jval.2011.07.012
21. De Bekker-Grob EW, Rose JM, Donkers B, et al. Men's preferences for prostate cancer screening: A discrete choice experiment. *British Journal of Cancer* 2013;108(3):533-41. doi: http://0-dx.doi.org.lib.exeter.ac.uk/10.1038/bjc.2013.5

For peer review only - http://bmjopen.bmj.com/site/about/guidelines.xhtml

22. Mansfield C, Ekwueme DU, Tangka FKL, et al. Colorectal Cancer Screening: Preferences, Past Behavior, and Future Intentions. *Patient* 2018:1-13. doi: http://0-dx.doi.org.lib.exeter.ac.uk/10.1007/s40271-018-0308-6
23. Miles A, Taylor SA, Evans RE, et al. Patient preferences for whole-body MRI or conventional staging pathways in lung and colorectal cancer: a discrete choice experiment. *European radiology* 2019;29(7):3889-900.
24. Fitch MI, Deane K, Howell D, et al. Women's experiences with ovarian cancer: reflections on being diagnosed. *Canadian Oncology Nursing Journal/Revue canadienne de soins infirmiers en oncologie* 2002;12(3):152-59.
25. Jelicic L, Brooker J, Shand L, et al. Experiences and health care preferences of women with ovarian cancer during the diagnosis phase. *Psycho-oncology* 2019;28(2):379-85.
26. Hol L, De Bekker-Grob EW, Van Dam L, et al. Preferences for colorectal cancer screening strategies: A discrete choice experiment. *British Journal of Cancer* 2010;102(6):972-80. doi: http://0-dx.doi.org.lib.exeter.ac.uk/10.1038/sj.bjc.6605566
27. van Dam L, Hol L, Bekker-Grob EWd, et al. What determines individuals' preferences for colorectal cancer screening programmes? A discrete choice experiment. *European Journal of Cancer* 2010;46(1):150-59. doi: http://0-dx.doi.org.lib.exeter.ac.uk/10.1016/j.ejca.2009.07.014
28. Salkeld G, Ryan M, Short L. The Veil of Experience: Do Consumers Prefer What They Know Best? *Health Economics* 2000;9(3):267-70. doi: http://onlinelibrary.wiley.com/journal/10.1002/%28ISSN%291099-1050/issues
29. Naik G, Ahmed H, Edwards AG. Communicating risk to patients and the public. *British Journal of General Practice* 2012;62(597):213-16.
30. Drummond M, Torbica A, Tarricone R. Should health technology assessment be more patient centric? If so, how?: Springer, 2020.

For peer review only - http://bmjopen.bmj.com/site/about/guidelines.xhtml

Figure 1: Example of a choice task

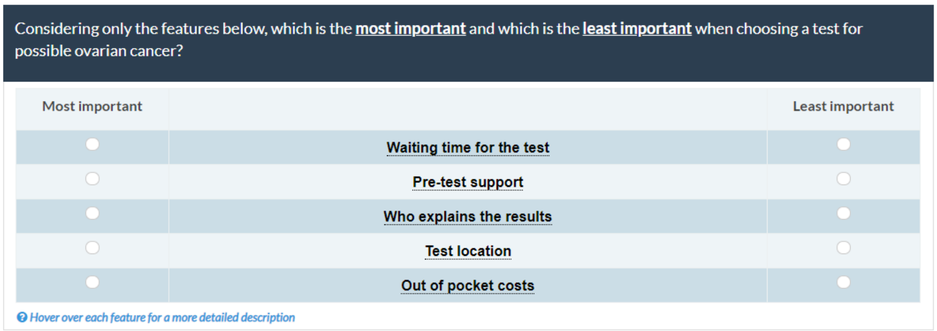

For peer review only

Figure 2: Best-worst scaling results. The distance between attributes is a spatial representation of the difference in relative importance between attributes on the latent importance scale.

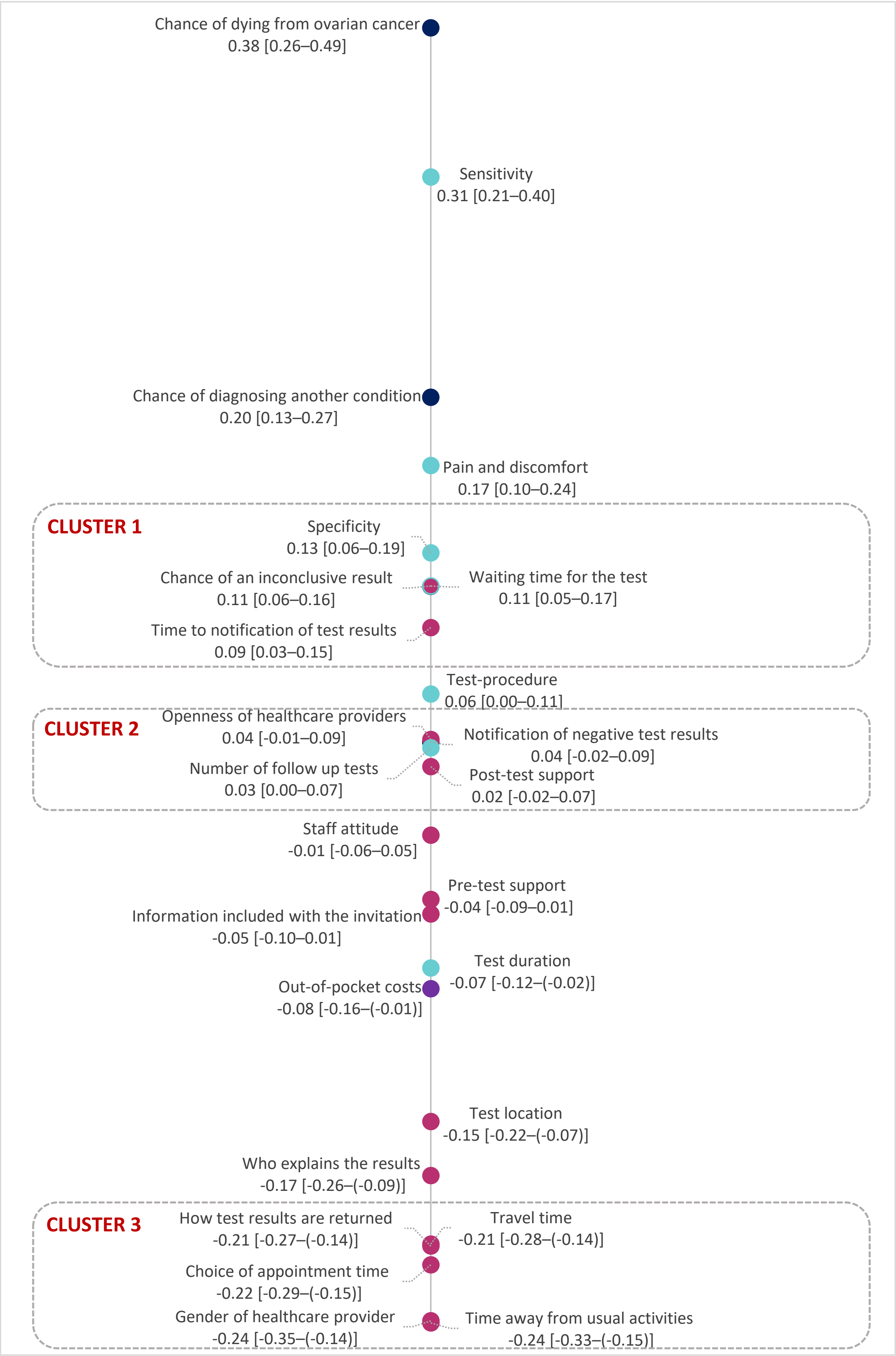

Figure 3: Sub-group analysis results comparing importance scores between those previously tested for ovarian cancer (n=40) and those who have never been tested (n=110). Error bars represent 95% confidence intervals (*=p-value <0.1, **= p-value <0.05, ***= p-value <0.01)

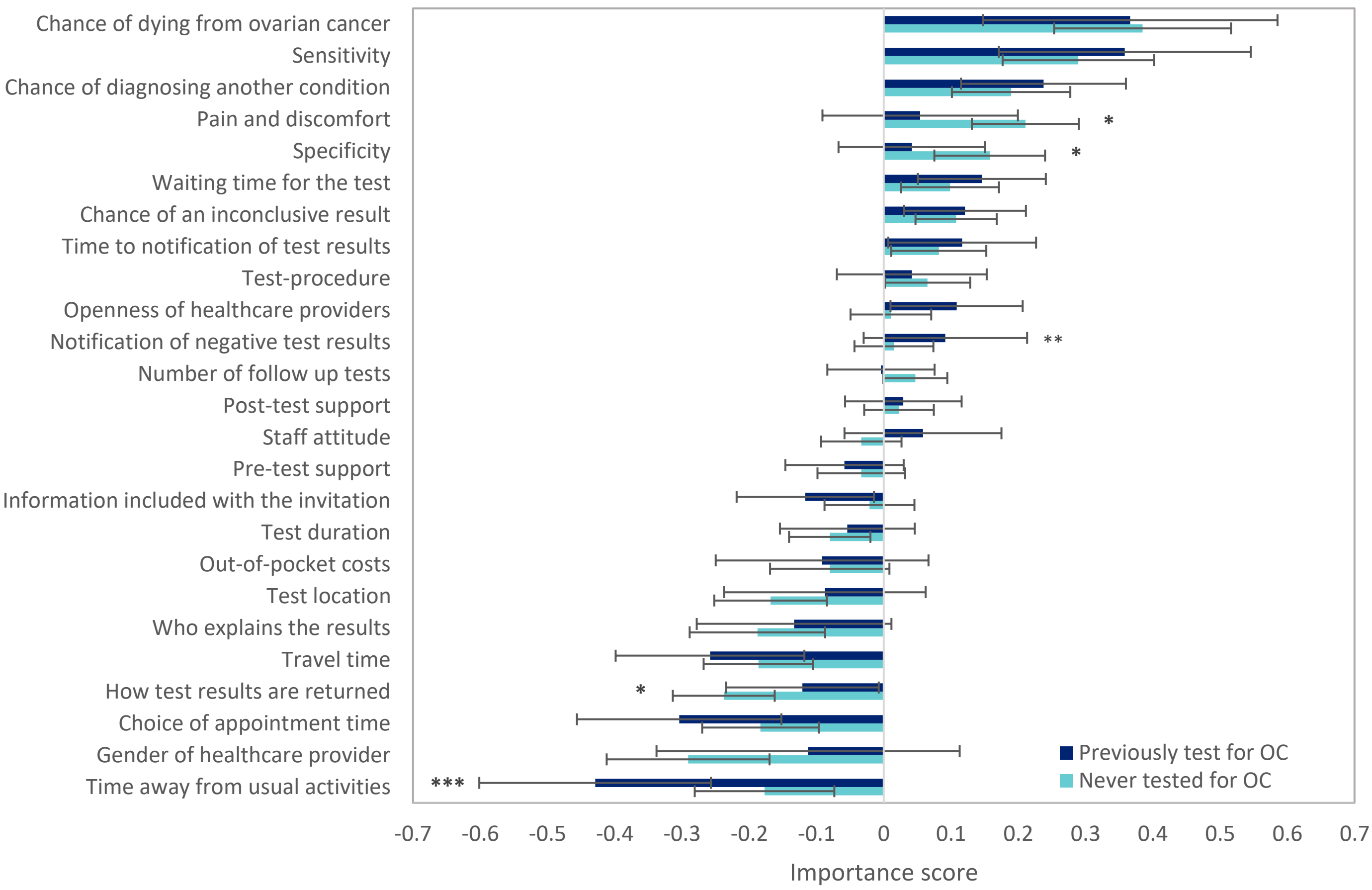

Subgroup differences: *=p-value <0.1, **= p-value <0.05, ***= p-value <0.01

For peer review only

Figure 4: Subgroup analysis results comparing importance scores between those who have previously undergone a transvaginal ultrasound for any medical reason (n=50) and those who have not (n=100).

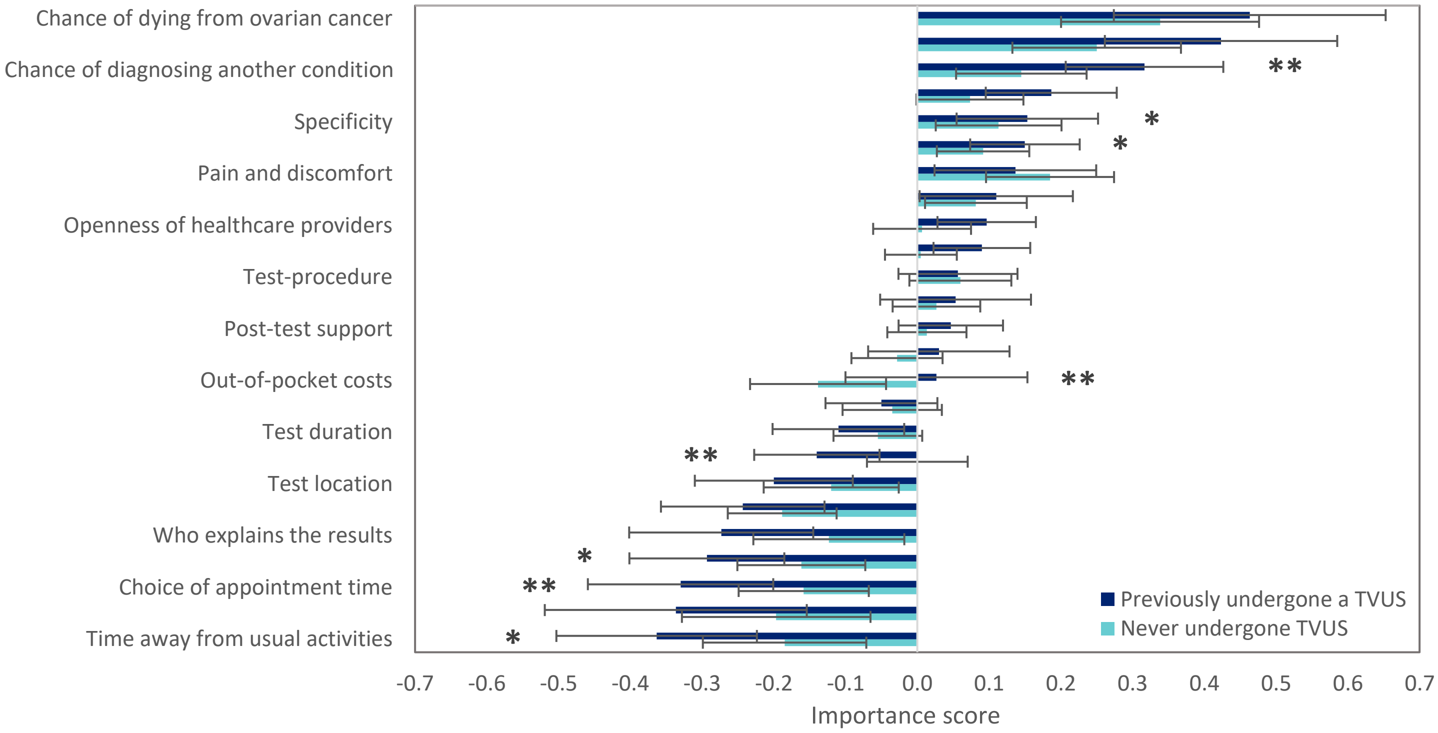

Significance of differences between subgroups: *=p-value<0.1, ** = p-value<0.05, ***=p-value<0.01

view only

Appendix 1: Health-related characteristics of population

Characteristic	n (%)
**Self-reported overall health**	
Very good	18 (12)
Good	61 (41)
Fair	47 (31)
Poor	14 (9)
Very poor	4 (3)
Not reported	6 (4)
**Perceived risk of ovarian cancer**	
Very High	4 (3)
High	12 (8)
Average	83 (55)
Low	33 (22)
Very low	12 (8)
Not reported	6 (4)
**Ovarian cancer-related worry**	
A great deal	4 (3)
A lot	25 (17)
A moderate amount	43 (29)
A little	47 (31)
Not at all	26 (17)
Not reported	5 (3)
**Personal history of cancer**	17 (13)
**Knew someone who was diagnosed with ovarian cancer**	25 (17)
**Previously tested for ovarian cancer**	40 (27)
**Previously undergone a TVUS (any reason)**	50 (33)
**Cervical screening**	
Always attends/attended	75 (50)
Irregularly attends/attended	37 (25)
Never attended/stopped attending	37 (25)
Unknown	1 (1)
**How much confidence and trust do you have in GPs**	
A great deal/a lot	59 (39)
A moderate amount	53 (35)
A little	25 (17)
None at all	2 (1)
Unknown	11 (7)
**How much do you feel able to be involved in medical decisions**	
A great deal	12 (8)
A lot	22 (15)
A moderate amount	58 (39)
A little	34 (23)
Not at all	17 (11)
Unknown	7 (5)
**How much do you wish to be involved in medical decisions**	
A great deal	60 (40)
A lot	67 (45)
A moderate amount	19 (13)
A little	3 (2)
Not at all	1 (1)

For peer review only

Appendix 2: Distribution of B-W scores across individuals. Demonstrating the degree of heterogeneity in preferences across individuals. Each attribute appeared within the survey six times meaning scores could range from 6 (always selected as most important) to -6 (always selected as least important).

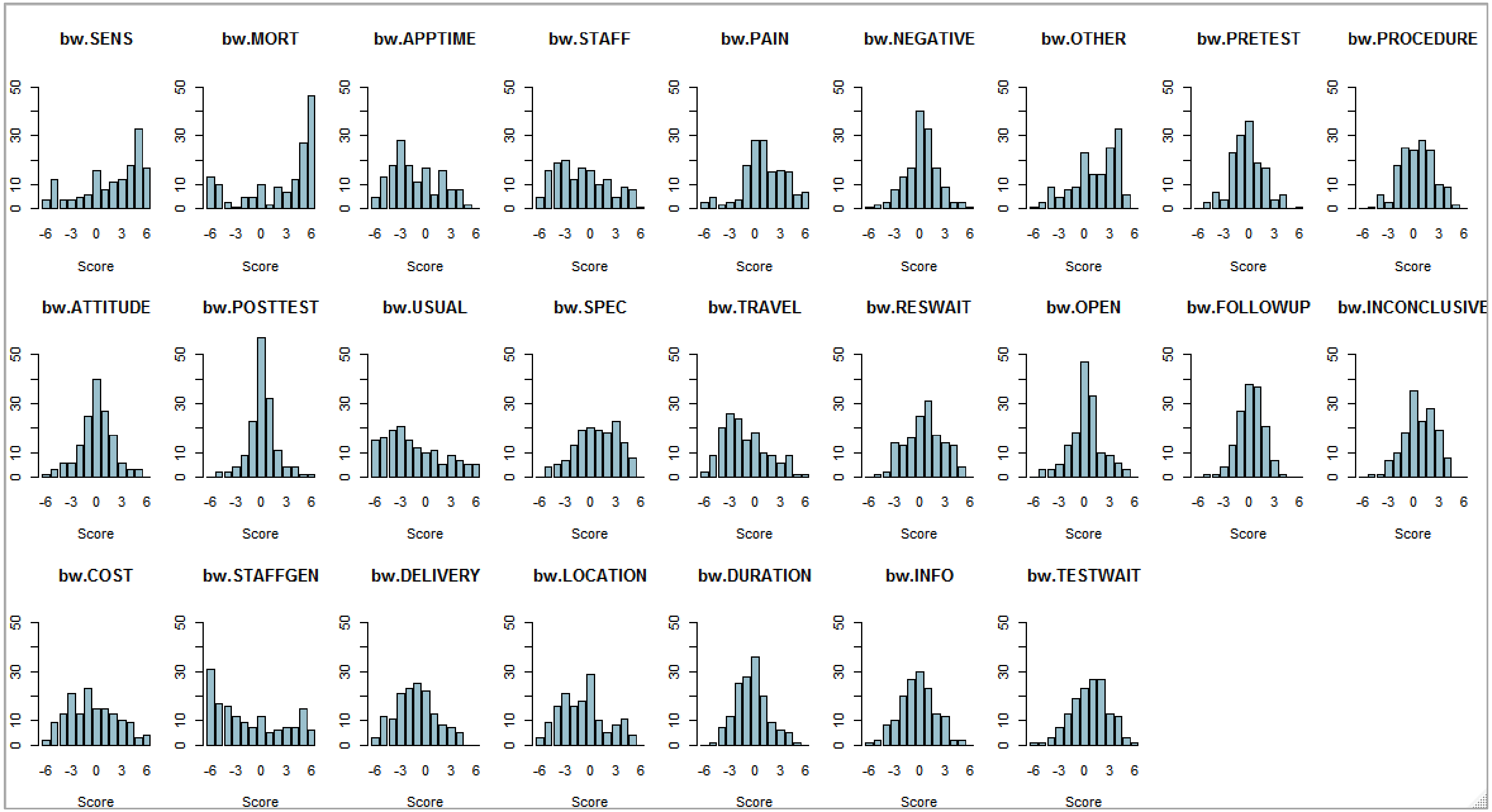

Appendix 3: Full summary of the best-worst scaling counting results

The shows the results of the relative importance of each attribute estimated using the counting approach. The top five attributes at each stage of analysis are highlighted green text and the bottom five are highlighted red text. In absolute terms, women most frequently selected the ability of a test to reduce the "*chance of dying from ovarian cancer*" as the most important factor (504), followed by the "*sensitivity*" of the test (410) (col.2). In contrast, *"time away from usual activities"* and *"gender of the healthcare provider"* were most frequently chosen as least important. Column 4 shows the combined best-worst scores at the aggregate level and column 5 shows the standardised average individual score for each attribute. Ranking highly in either the "most" or "least" important dimension in isolation did not necessarily translate to ranking most or least important overall once the two dimensions were combined, demonstrating high levels of heterogeneity. Most-least scores were bound between -900 and 900 (each attribute occurred 6 times x 150 respondents) and whilst standardised individual scores were bound between -1 and 1.

Attributes	Most	Least	Most-Least	Counting			Ranking
				Standardised score	SD	95% confidence interval	
Sensitivity	410	133	277	0.308	0.60	0.21–0.40	2
Chance of dying from ovarian cancer	504	162	342	0.380	0.70	0.26–0.49	1
Choice of appointment time	122	316	-194	-0.216	0.47	-0.29–(-0.15)	23
Who explains the results	150	306	-156	-0.173	0.52	-0.26–(-0.09)	20
Pain and discomfort	246	94	152	0.169	0.44	0.10–0.24	4
Notification of negative test results	135	103	32	0.036	0.34	-0.02–0.09	11
Chance of diagnosing another condition	289	107	182	0.202	0.45	0.13–0.27	3
Pre-test support	111	147	-36	-0.040	0.33	-0.09–0.01	15
Test-procedure	177	124	53	0.059	0.34	0.00–0.11	9
Staff attitude	120	128	-8	-0.009	0.34	-0.06–0.05	14
Post-test support	106	84	22	0.024	0.28	-0.02–0.07	13
Time away from usual activities	138	358	-220	-0.244	0.56	-0.33–(-0.15)	25
Specificity	239	125	114	0.127	0.42	0.06–0.19	5
Travel time	107	292	-185	-0.206	0.44	-0.28–(-0.14)	21
Time to notification of test results	194	112	82	0.091	0.37	0.03–0.15	8
Openness of healthcare providers	134	101	33	0.037	0.32	-0.01–0.09	10
Number of follow up tests	129	99	30	0.033	0.25	-0.00–0.07	12
Chance of an inconclusive result	174	74	100	0.111	0.31	0.06–0.16	6
Out-of-pocket costs	154	229	-75	-0.083	0.48	-0.16–(-0.01)	18
Gender of healthcare provider	182	401	-219	-0.243	0.67	-0.35–(-0.14)	24
How test results are returned	104	290	-186	-0.207	0.40	-0.27–(-0.14)	22
Test location	132	264	-132	-0.147	0.46	-0.22–(-0.07)	19
Test duration	95	161	-66	-0.073	0.32	-0.12–(-0.02)	17
Information included with the invitation	135	177	-42	-0.047	0.35	-0.10–0.01	16
Waiting time for the test	213	113	100	0.111	0.37	0.05–0.17	6

Key: Five best scoring attributes in each round, Five worst scoring attributes in each round.
For the "least" column best and worst are inversed so worst scoring = rated least important most frequently

For peer review only

BMJ Open

Women's priorities towards ovarian cancer testing: A best-worst scaling study

Journal:	*BMJ Open*
Manuscript ID	bmjopen-2022-061625.R1
Article Type:	Original research
Date Submitted by the Author:	21-Jun-2022
Complete List of Authors:	Hall, Rebekah; University of Exeter, Health Economics Group<br>Medina-Lara, Antonieta; University of Exeter Medical School, Health Economics Group<br>Hamilton, Willie; University of Exeter, Primary Care Diagnostics<br>Spencer, Anne; University of Exeter Medical School, Health Economics
**Primary Subject Heading**:	Oncology
Secondary Subject Heading:	Health economics, Patient-centred medicine
Keywords:	Gynaecological oncology < GYNAECOLOGY, Health economics < HEALTH SERVICES ADMINISTRATION & MANAGEMENT, Gynaecological oncology < ONCOLOGY, Ultrasound < RADIOLOGY & IMAGING

SCHOLARONE™
Manuscripts

I, the Submitting Author has the right to grant and does grant on behalf of all authors of the Work (as defined in the below author licence), an exclusive licence and/or a non-exclusive licence for contributions from authors who are: i) UK Crown employees; ii) where BMJ has agreed a CC-BY licence shall apply, and/or iii) in accordance with the terms applicable for US Federal Government officers or employees acting as part of their official duties; on a worldwide, perpetual, irrevocable, royalty-free basis to BMJ Publishing Group Ltd ("BMJ") its licensees and where the relevant Journal is co-owned by BMJ to the co-owners of the Journal, to publish the Work in this journal and any other BMJ products and to exploit all rights, as set out in our licence.

The Submitting Author accepts and understands that any supply made under these terms is made by BMJ to the Submitting Author unless you are acting as an employee on behalf of your employer or a postgraduate student of an affiliated institution which is paying any applicable article publishing charge ("APC") for Open Access articles. Where the Submitting Author wishes to make the Work available on an Open Access basis (and intends to pay the relevant APC), the terms of reuse of such Open Access shall be governed by a Creative Commons licence – details of these licences and which Creative Commons licence will apply to this Work are set out in our licence referred to above.

Other than as permitted in any relevant BMJ Author's Self Archiving Policies, I confirm this Work has not been accepted for publication elsewhere, is not being considered for publication elsewhere and does not duplicate material already published. I confirm all authors consent to publication of this Work and authorise the granting of this licence.

For peer review only

Title: Women's priorities towards ovarian cancer testing: A best-worst scaling study

Rebekah Hall[a], Antonieta Medina-Lara[a], Willie Hamilton[b], Anne E Spencer[a]

[a] Health Economics Group, College of Medicine and Health, University of Exeter, Exeter, UK

[b] Primary Care Diagnostics, College of Medicine and Health, University of Exeter, Exeter, UK

Corresponding Author:

Rebekah Hall

Address: University of Exeter, St Luke's Campus, Heavitree, Exeter, EX1 2LU, UK

Email: rh591@exeter.ac.uk

Phone: +44 (0)1392 725565

ORCID ID:

Rebekah Hall: 0000-0003-1804-993X

Antonieta Medina-Lara: 0000-0001-7325-8246

Willie Hamilton: 0000-0003-1611-1373

Anne Spencer: 0000-0002-8163-3103

For peer review only

Word counts: Abstract: 262 Main body: 3305

Abstract:

Objective: To investigate the importance of key characteristics relating to diagnostic testing for ovarian cancer and to understand how previous test experience influences priorities.

Design: Case 1 best-worst scaling embedded in an online survey

Setting: Primary care diagnostic testing in England and Wales

For peer review only - http://bmjopen.bmj.com/site/about/guidelines.xhtml

Participants: 150 women with ovaries over 40 years old living in England and Wales.

Methods: We used best-worst scaling, a preference-based survey method, to elicit the relative importance of twenty-five characteristics relating to ovarian cancer testing following a systematic review. Responses were modelled using conditional logit regression. Sub-group analysis investigated variations based on testing history.

Main outcome measures: Relative importance scores

Results: “Chance of dying from ovarian cancer” (0.380, CI[0.26–0.49]) was the most important factor to respondents, closely followed by “test sensitivity” (0.308, CI[0.21–0.40]). In contrast, “time away from usual activities” (-0.244, CI[-0.33–(-0.15)]) and “gender of healthcare provider” (-0.243, CI[-0.35–(-0.14)]) were least important to respondents overall. Women who had previously undergone testing placed higher importance on certain characteristics including “openness of healthcare providers” and “chance of diagnosing another condition” at the expense of reduced emphasis on characteristics such as “pain and discomfort” and “time away from usual activities”.

Conclusions: The results clearly demonstrated items at the extreme, that were most and least important to women considering ovarian cancer testing. Differences in priorities by testing history demonstrate an experience effect, whereby preferences adapt over time based on evidence and experience. Acknowledging these differences helps to identify underlying barriers and facilitators for women with no test experience as well as shortcomings of current service based on women with experience.

Strengths and limitations:

- This study adds to a very limited evidence base of studies assessing priorities towards diagnostic testing for cancer and specifically ovarian cancer.
- Selection of included characteristics is based on a rigorous, published systematic review and utilised patient and public involvement to ensure the relevance to the target population

For peer review only

For peer review only - http://bmjopen.bmj.com/site/about/guidelines.xhtml

- Case 1 best worst scaling is ideal for establishing the relative importance of a large number of characteristics and has been proven to be easier to complete and more effective than alternative methods such as ranking or ratings tasks
- A key limitation of the study relates to the representativeness of the sample. Due to the recruitment method, the sample is not fully representative of the population in key demographics including ethnicity and age distribution
- The lack of discrimination between lower scoring attributes may be reflective of genuine priorities. However, it is also possible that choice task construction and sample size were contributing factors

1. Introduction

Ovarian cancer is the seventh most common cancer in women worldwide, with over 200,000 new cases and approximately 180,000 deaths annually.[1] Five-year survival rates for the disease are highly dependent on a number of factors including the patient's age, country of residence and tumour type.[2] Late-stage diagnosis contributes heavily to the high mortality rates associated with ovarian cancer and is an ongoing challenge globally.[3] For instance, in the UK, almost 60% of cases are diagnosed at stage III or IV where average five-year survival is just 26.9% and 13.4% respectively.[4] Improving diagnostic outcomes is multifaceted problem, however, delays in help-seeking upon symptom onset and access to timely testing have been identified as challenges to earlier diagnosis.[5]

National guidelines for the investigation of suspected ovarian cancer in symptomatic women vary substantially between countries.[6] Existing tests include the CA125 blood test and imaging tests, most commonly a transabdominal/transvaginal ultrasound (TVUS) but also Computed Tomography (CT) and Magnetic Resonance Imaging (MRI).[7] Variations in guidance represents uncertainty around the accuracy of existing test strategies, especially for the early investigation of symptoms. Evidence

For peer review only - http://bmjopen.bmj.com/site/about/guidelines.xhtml

evaluating the performance of diagnostic tests in a primary care setting is very limited.[8] Furthermore, for recommendations to be effective, clinical guidelines must also consider the preferences of those offered testing, particularly in healthcare systems emphasising shared-decision making.[9] Available tests differ substantially, not only in terms of performance but also service delivery and patient experience. Aspects of tests may be prioritised differently; for instance, patients may accept a lower levels of accuracy a less invasive test. Thus, it may be necessary to weight characteristics differently when considering the overall balance of benefits and harms.

To date, very little attention has been paid to the priorities of women facing ovarian cancer testing, particularly in a diagnostic setting. Existing studies usually focus on screening trials[10], single test modalities [11] or single aspects of acceptability, such as pain.[12] To address the current evidence gap, we aimed to elicit preferences for twenty-five key characteristics ('items') of ovarian cancer testing, using best-worst scaling (BWS). This is a stated preference technique that has been demonstrated to provide higher predictive values than ranking or rating methods whilst being less cognitively demanding.[13] Understanding the importance of key characteristics allows aspects of greatest importance to be given increased salience in future decisions about testing, particularly in guideline revision.

Previous studies have demonstrated views may differ based on previous experience of the health event. We therefore examined the relationship between priorities around ovarian cancer testing and test experience.[14 15]

2. Methods

2.1 Best worst scaling

We used Case 1 best-worst scaling (BWS) method to identify women's preferences for characteristics associated with diagnostic testing for ovarian cancer. This type of BWS aims to assess the relative importance of, or preference for, items based on the underlying principles of random utility

For peer review only - http://bmjopen.bmj.com/site/about/guidelines.xhtml

theory.[16] BWS was initially developed in marketing but has been increasingly utilised in healthcare research for explorations of patient and stakeholder preferences.[16-18] In particular, Case 1 BWS has been demonstrated to be an effective alternative to traditionally used ranking or ratings tasks when considering patient priorities.[19]

During BWS tasks, participants respond to a series of choice tasks presenting a subset of items and asked to select the “best” or “most important” and “worst” or “least important” item. Simultaneously examining items selected as “most” and “least” important provides greater information than examining the most important item alone. Analysing responses to choice tasks allows the underlying relative importance of items to be inferred and a ranking of included items to be established[19].

2.2 Identification of relevant items

Results demonstrate the importance of an item, relative to the other included item. It is therefore important to use a rigorous selection process to ensure the most salient characteristics are included in the experiment. We performed a systematic review of the preference-based literature to identify characteristics relating to cancer testing.[20] Potential characteristics were then narrowed down by the authors based on relevance to ovarian cancer testing in symptomatic patients using an iterative Delphi method process where exclusion required full agreement of the research team. In total, twenty-five characteristics were selected for inclusion (Table 1).

For peer review only

1 Table 1: Characteristics included in the BWS study (wording and descriptions are identical to those
2 shown to respondents during the survey)

	Characteristic and definition
1	**Test sensitivity**<br>*Chance that the test will miss cancer in a patient who actually does have the disease (false-negative result)*
2	**Chance of dying from ovarian cancer**<br>*How much having the test decreases the chance of dying from ovarian cancer*
3	**Choice of appointment time**<br>*Whether a person can choose an appointment time or if the appointment time is assigned by the health care provider*
4	**Who explains the results**<br>*Type of health care provider who explains the test results e.g. Nurse, doctor etc.*
5	**Pain and discomfort**<br>*The level of pain and/or discomfort experienced during and after the test*
6	**Notification of negative test results**<br>*Whether you are contacted if your results are normal*
7	**Chance of diagnosing another condition**<br>*If symptoms are not caused by cancer, the chance the test can identify other reason for the symptoms*
8	**Pre-test support**<br>*Level of support received before having the test describing what will happen during the test and what the results might show*
9	**Test procedure**<br>*What having the test will involve. For ovarian cancer this could be a blood test or an transvaginal ultrasound (internal ultrasound of the reproductive organs)*
10	**Staff attitude**<br>*How the health care provider treats you while conducting the test*
11	**Post-test support**<br>*Level of support received after getting the results of the test relating to the meaning of your results and what will happen next*
12	**Time away from usual activities**<br>*The total amount of time spent having the test instead of doing your usual daily activities*
13	**Test specificity**<br>*Chance of unnecessary further invasive testing (false-positive result)*
14	**Travel time**<br>*The total amount of time spent travelling to and from the test*
15	**Time to notification of results**<br>*The length of time it takes to hear the results after having the test*
16	**Openness of health care providers**<br>*How open health care providers are with their thoughts about the cause of your symptoms and the tests they recommend*
17	**Number of follow up tests**<br>*How many additional tests are needed to confirm a diagnosis*
18	**Chance of an inconclusive result**<br>*The chance the results are unclear and the test would need to be repeated after a waiting period*
19	**Out of pocket costs**<br>*How much it will personally cost a person to have the test e.g. Travel costs, childcare costs, time off work etc. The cost of the test is covered by the NHS*

For peer review only - http://bmjopen.bmj.com/site/about/guidelines.xhtml

For peer review only

20	**Gender of healthcare provider**<br>*Gender of the staff member giving you the test*
21	**How test results are returned**<br>*e.g. in person, phone, letter*
22	**Test location**<br>*Where the test takes place*
23	**Test duration**<br>*The length of time spent having the test*
24	**Information included with the invitation**<br>*The level and type of information received about the test*
25	**Waiting time for the test**<br>*How long a person has to wait to have the test after being referred by their GP*

2.3 BWS task construction

A balanced incomplete block design (BIBD) generated using SAS 9.4 was used to construct the BWS choice sets for the 25 items. BIBD designs ensure each item appears equally often, and pairwise comparisons between each of the items occurs equally across the design.[21] A final design (d-efficiency of 83.3%) consisting of 30 choice tasks each with five items was selected based on a trade-off between survey complexity and design efficiency. Each item appeared six times across the choice tasks and coappeared with remaining attributes once throughout the experiment. The position of the item within choice tasks was optimised such that items were listed in 1st-5th position equally and the order of tasks was randomised across participants to control for any order effects. Each participant completed all thirty tasks. An example of a BWS task is shown in Figure 1.

2.4 Questionnaire

The questionnaire was developed and collected using LimeSurvey, an online survey platform. The survey consisted of four stages:

i. Socio-demographic questions e.g. age, education, employment status
ii. Best-worst scaling questions (including a warm-up task)
iii. Task feedback questions e.g. task difficulty

For peer review only

For peer review only - http://bmjopen.bmj.com/site/about/guidelines.xhtml

iv. Background questions relating to health-related characteristics e.g. self-reported health, testing history, current and desired in medical decisions

Question framing and answer categories for stages i and iii-iv were copied or adapted from established national surveys. The survey was shared and discussed with the Cancer Research UK funded CanTest PPI lead, Margaret Johnson. Amendments in survey wording were made as a result of discussions. Early piloting with five women suggested the survey would take 30 minutes to complete. Given the length of the survey and online administration method, three attention checks following the instructional manipulation format were embedded (e.g. "Select 'very important' to indicate you are paying attention").[22] Respondents who failed all three attention checks were removed from the analysis. A full version of the questionnaire is available upon request.

2.5 Participants

A sample size of 150 women was estimated to be required. Participants were recruited via Prolific, an online platform for researchers conducting social science experiments. Results from this platform have been shown to be of comparable or better quality than university research lab registers and have been used widely within hundreds of published studies across disciplines. [23 24] Our study focused on the priorities of women over 40 (no upper limit), the group most likely to be offered testing.[25] Participation was also limited to those living in England and Wales: no other limitations were applied. The study was approved by the University of Exeter UEBS-REC (eUEBS003725). Respondents completed an electronic consent form before completing any questions.

2.6 Statistical analysis

Descriptive statistics relating to sociodemographic and health-related characteristics of the sample were summarised.

Best-worst responses were initially analysed using the counting approach; whereby the number of times an item was picked as 'least important' is subtracted from the number of times it was chosen

For peer review only - http://bmjopen.bmj.com/site/about/guidelines.xhtml

as 'most important'. Each item appeared 6 times across all tasks, meaning best-worst scores could range from -6 to +6 at the individual level. Individual scores were aggregated and standardised to calculate an overall mean score for the sample ranging from -1 to +1[1]. A score close to +1 indicates an item is highly influential, whereas item with scores near to -1 demonstrate much less relevance.

Next, conditional logit regression using dummy-coded items was used to model responses. "Time away from usual activities" was identified as the least important attribute during counting analysis and was omitted from the model and used as the reference item. As a result, all parameters were expected to be positive. To aid interpretation, we rescaled conditional logit coefficients using standardised ratio scores where all scores sum to 100.[26 27] Confidence intervals for relative importance scores were estimated using the delta method. All analyses were performed using Stata 17.[28]

In both the counting and conditional logit analysis, scores relate to the relative importance of attributes (i.e. relative importance scores). In other words, differences between scores are meaningful whereas absolute values are not.

2.6.1 Subgroup analysis

Subgroup analysis was performed to explore the relationship between previous testing and preferences for future testing. Experience was captured by two questions that asked whether women had previously: 1) undergone testing for ovarian cancer or 2) received a transvaginal ultrasound for any reason. Subgroup analysis was based on conditional logit results and differences in relative importance scores between groups were tested using unpaired T-tests. Finally, heteroscedastic logit models were estimated to investigate whether differences between subgroup were attributable to scale differences (i.e. differences in error variance between subgroups) or due to a genuine difference in preferences.

---

[1]Scores were standardised using the following equation: population-level best-worst score/(number of times item appeared x total sample size).

For peer review only - http://bmjopen.bmj.com/site/about/guidelines.xhtml

3 Results

In total 159 women responded to the survey. The average response time for the questionnaire was 29 mins 51 seconds. Four submissions were incomplete, two were removed due to failing all attention checks, and a further three responses were removed due to incorrect completion of the best-worst section of the survey, resulting in 150 responses for the final analysis. Respondents varied substantially in how difficult they found the best-worst portion of the questionnaire with 42% (63/150) reporting it easy/very easy but 38% (57/150) finding it difficult/very difficult. There were no significant differences in BWS responses between those who found the task difficult versus those who did not.

3.1 Demographics

The demographics of the sample are presented in Table 2. The age ranged from 40 to 87 years old with a mean of 51.4 (SD=9.1). Most participants were white (120/150; 80%), married/in a relationship (97/150; 65%) and employed (78/150; 52%).

Most women perceived their risk of cancer as low-average (128/150; 85%) and ovarian cancer-related anxiety was generally low-moderate amongst respondents (116/150; 77%). Overall, fifty women (33%) reported previously undergoing a TVUS for any reason. Forty (27%) women reported being previously tested for ovarian cancer, with CA125 blood test being the most common test.

Crucially, when asked, 127/150 women (89%) stated they wished for a great deal/a lot of involvement in decisions relation to their own care but only 34/150 (23%) currently felt this was achieved, with 17/150 (11%) respondents felt unable to be involved in medical decisions at all. Further details are found in Appendix 1.

For peer review only - http://bmjopen.bmj.com/site/about/guidelines.xhtml

Table 2: Descriptive characteristics: sociodemographic

Characteristic	n (%)
**Age**	
Mean (SD)	51.4 (9)
Range	40-87
**Ethnicity**	
White	120 (80)
Asian	8 (5)
Black	3 (2)
Mixed	3 (2)
Other	9 (6)
Not reported	7 (4)
**Number of children, mean (SD)**	1.3 (1)
**Relationship status**	
Married	75 (50)
In a relationship	22 (15)
Single	19 (13)
Divorced/separated	26 (17)
Widowed	6 (4)
Not reported	2 (1)
**Level of education**	
GCSE	37 (25)
A-Level/ College	25 (17)
Undergraduate	41 (27)
Post-graduate	35 (23)
No qualifications	1 (1)
Other	9 (6)
Not reported	2 (1)
**Employment status**	
Full-time	47 (31)
Part-time	32 (21)
Self-employed	23 (15)
Not employed	11 (7)
Retired	14 (9)
Other	18 (12)
Not reported	5 (3)

3.2 Best-worst results

3.2.1 Counting analysis

BWS results are presented in Appendix 2. Scores were bound between -1 and 1. Scores tending toward the extremes of the scale would imply homogeneity across respondents and consistency between responses across questions on an individual level. Importance scores ranged from -0.224 to 0.380,

For peer review only - http://bmjopen.bmj.com/site/about/guidelines.xhtml

suggesting high levels of heterogeneity in preferences regarding test characteristics across respondents (see Appendix 3).

Overall, "*Chance of dying from ovarian cancer*" (0.380, CI[0.26–0.49]) was most important to women when considering ovarian cancer testing, followed by "*Test sensitivity*" (0.308, CI[0.21–0.40]). Conversely, "*Time away from usual activities*" (-0.244, CI[-0.33–(-0.15)]) and "*Gender of healthcare provider*" (-0.243, CI[-0.35–(-0.14)]) were considered least important to women when facing diagnostic testing and were statistically indistinguishable from each other.

3.2.2 Conditional logit analysis

The results for the conditional logit model are shown in Appendix 2. The order of importance remained consistent across the two analysis methods and the estimates were highly correlated (Appendix 4).

All items had a positive coefficient and most were statistically significant at the 95% level, confirming the relative importance of all attributes compared to "*time away from usual activities*". Non-significant items were those with the lowest importance (e.g. "*gender of healthcare providers*" and "*how test results are returned*"), suggesting a clustering effect towards the bottom of the importance scale.

Figure 2 shows the relative importance scores associated with the conditional logit estimates. The distance between each item is a spatial visualisation of differences in relative importance. Importance scores ranged from a maximum of 9.88 [95% CI: 7.04—12.72] for "Chance of dying of ovarian cancer" to a minimum of 1.93 [95% CI:1.34—2.52] for "Time away from usual activities" and Gender of healthcare providers. Indicating "chance of dying from ovarian cancer" was approximately five times more important to respondents. The most important characteristics to respondents were clear and distinct: however, spatial visualisation demonstrated grouping of attributes towards the centre and bottom of the scale. Groupings were distinct to other items but differences in importance between items within clusters is less distinguishable. In general, results demonstrate a clear prioritisation of

For peer review only - http://bmjopen.bmj.com/site/about/guidelines.xhtml

outcome (dark blue dots) and test specific characteristics (light blue dots) whereas service delivery characteristics (pink dots) were consistently less important to respondents.

3.2.3 Subgroup analysis

Women previously tested for ovarian cancer placed significantly lower importance on "*pain and discomfort*" in comparison to test-naïve individuals. There was also evidence to suggest "*time away from usual activities*" and "*test specificity*" were less prioritised by previously tested women whilst "*openness of healthcare providers*" and "*test specificity*" appeared marginally more important to those with test experience (Figure 3).

For women who had previously undergone a TVUS, "*chance of diagnosing another condition*" and "*chance of dying from ovarian cancer*" were significantly more important compared to those with no test experience (Figure 4). Alternatively, women who had never been tested appeared to place higher value on "*information included with the invitation*" and "*choice of appointment time*", although the overall importance of such attributes remained relatively low.

In both instances, results from heteroscedastic logit models demonstrated no differences in scale between subgroups indicating observed differences were attributable to differences in priorities as opposed to differences in response variance between groups with different prior experience (Appendix 5).

4 Discussion

4.1 Summary of main findings

This is the first study to investigate the priorities of women relating to diagnostic testing for ovarian cancer. The results of this study highlight the importance yet current neglect of incorporating the preferences of patients into medical testing decisions. When asked, almost 90% (134/150) of respondents wanted heavy involvement in medical decisions: however, less than a quarter of women currently felt able to do so.

For peer review only - http://bmjopen.bmj.com/site/about/guidelines.xhtml

The results are particularly useful in identifying items at either extreme of the scale, clearly demonstrating the characteristics that are most and least important to women considering ovarian cancer. Low concurrence between items and high levels of heterogeneity meant discrimination between mid-range items was less clear. Overall, "*chance of dying from ovarian cancer*", "*test sensitivity*" and "*chance of diagnosing another condition*" were the most important characteristics when considering testing for ovarian cancer. Oppositely, "*time away from usual activities*" and "*gender of healthcare provider*" were the least important factors. The priorities of previously tested women were generally similar to women who had never undergone testing, but there were a few key differences between these two groups suggesting experience is an important determinant of priorities.

4.2 Results in the Context of Published Literature

The prioritisation of outcome and test-specific characteristics and the relative lack of importance placed on service delivery characteristics is reflective of findings from the wider literature examining preferences towards cancer screening and diagnosis.[20 29-32] The relative importance of "*openness of healthcare providers"* is echoed by qualitative research exploring the diagnostic experiences of women with ovarian cancer where shortcomings in doctor-patient communication was a recurring theme. In particular, patients raised concerns about doctors' willingness and ability to openly communicate and share information.[33 34] Decreased importance of particular characteristics to tested women may suggest these aspects of testing are already being achieved/acceptable within the current system. For example, the decreased importance of "*pain and discomfort*" for both women who have been tested for ovarian cancer and women who have previously received an ultrasound (NS) is expected given the finding that almost 80% of women who had previously undergone a transvaginal ultrasound experienced little to no pain.[12]

Existing studies examining differences in preferences towards cancer testing between previously tested and untested individuals have typically focused on colorectal cancer screening, but also found

For peer review only - http://bmjopen.bmj.com/site/about/guidelines.xhtml

evidence of statistically different preferences between the two groups.[35 36] Hol, et al. [35] found previously screened individuals displayed stronger and more positive preferences towards different screening modalities and shorter screening intervals than unscreened counterparts. In a similar study, van Dam, et al. [36] found limited differences between people with and without screening experience (mortality risk reduction only), however, preferences across previously screened individuals differed significantly based on the particular screening modality received. These results are evidence of an experience effect, via "status quo bias", where individuals place a higher value on goods or services that are more familiar.[37]

4.3 Strengths and Limitations

Best-worst scaling provides a straightforward method for capturing the priorities of women. The method is preferable to ranking/rating tasks due to its ability to measure the importance of large numbers of items whilst limiting complexity and cognitive burden.[38]

The survey length and high number of items represent a potential limitation. In total, 38% of respondents reported the task as difficult/very difficult. Despite this, drop-out rates were low (3% (4/150) of people began the survey but did not complete) and there were no differences between responses according to reported task difficulty.

Poor discrimination between lower scoring items may be reflective of genuine equality in priorities, however, it is also feasible that the choice task construction was a contributing factor. For example, the ability to detect differences will be affected by the number of pairwise comparisons between attributes. Selection of a BIBD with more than one pairwise comparison between each item may have increased the explanatory power of the study, of course this requires a trade-off with survey length and sample size requirements where blocking is required. Similarly, a larger sample size may have increased the explanatory power and also allowed for more complex statistical investigation of

For peer review only - http://bmjopen.bmj.com/site/about/guidelines.xhtml

preference heterogeneity. To our knowledge, there is currently no accepted guidance on sample size requirements within object case BWS, although theories from the closely related methods, such as discrete choice experiments may be transferable.[39]

Finally, due to the recruitment method, the sample is not fully representative of the population in key demographics including ethnicity and age distribution. Further research is needed to understand whether results are generalisable to a wider population.

4.4 Implications for Practice and Future Research

The findings of this study offer useful insights into potential barriers and facilitators of undergoing testing in a timely manner. Interestingly, characteristics involving an element of risk or uncertainty dominated the top-ranking positions. However, how to best explain complex aspects of test performance such as sensitivity and specificity to patients is a clear challenge.[40]

Differences in importance between women with and without test experience suggest that priorities are continuously adapted based on evidence and experience gained over time. When considering policy decisions, it is therefore important to carefully consider whose views should be prioritised - a longstanding debate within the field of health technology assessment.[41] Arguably, it is important to consider both perspectives; priorities of women with test experience help to identify unmet or inadequate aspects of current service provision, whereas preconceived views of testing-naïve women may reveal underlying barriers and facilitators of testing since the initial decision to undergo testing is based on these pre-existing judgements. In both instances, mismatches in priorities and practice may lead to delays in seeking help or testing for future symptoms.

High levels of heterogeneity between individuals highlight the importance of a personalised approach to patient interactions throughout the diagnostic process. Patients are likely to have different concerns and priorities during this time, however, the importance of patient-input and shared

For peer review only - http://bmjopen.bmj.com/site/about/guidelines.xhtml

decision-making appears to be less prioritised in diagnostic settings compared to decisions regarding cancer screening and subsequent treatment where preferences have been studied more extensively.

5. Conclusion

Preferences towards diagnostic testing have been underexplored to date. Understanding what matters most to patients may reduce anxiety around testing, facilitate earlier help-seeking behaviour and improve patient satisfaction. This study highlights that test sensitivity and mortality impacts are the most important factors to patients facing ovarian cancer testing. However, results varied significantly across individuals demonstrating the need for an individualised approach to consultations regarding diagnostic care. Our results can help inform policies and diagnostic guidelines designed to encourage earlier help-seeking behaviour as well as help to evaluate the patient-friendliness of emerging test strategies for suspected ovarian cancer.

Conflicts of interest: No conflicts of interest

Funding statement: This research arises from the CanTest Collaborative, which is funded by Cancer Research UK (reference number: C8640/A23385), of which Rebekah Hall is a funded PhD student, Willie Hamilton is director and Anne E Spencer is a senior faculty member.

Ethical approval: This study was approved by the University of Exeter UEBS-REC in July 2020. Reference number: eUEBS003725.

Author contributions: All authors contributed to the study conception and design. RH and AM-L conducted data analysis. RH prepared the first draft of the manuscript and AS, AM-L and WH edited and approved the final version.

Acknowledgements: The authors thank Margaret Johnson, CanTest PPI Panel lead, for providing feedback during the development of this study.

For peer review only - http://bmjopen.bmj.com/site/about/guidelines.xhtml

Data sharing statement: Anonymised survey data and analysis code are available from the corresponding over upon reasonable request.

References

1. Reid BM, Permuth JB, Sellers TA. Epidemiology of ovarian cancer: a review. *Cancer biology & medicine* 2017;**14**(1):9.
2. Matz M, Coleman MP, Carreira H, et al. Worldwide comparison of ovarian cancer survival: Histological group and stage at diagnosis (CONCORD-2). *Gynecologic oncology* 2017;**144**(2):396-404.
3. Bhatla N, Jones A. THE WORLD OVARIAN CANCER COALITION ATLAS. 2018.
4. Cancer Research UK. Ovarian Cancer Statistics Secondary Ovarian Cancer Statistics 2019. https://www.cancerresearchuk.org/health-professional/cancer-statistics/statistics-by-cancer-type/ovarian-cancer#heading-Two.
5. Target Ovarian Cancer. Pathfinder 2016. Transforming futures for women with ovarian cancer, 2016.
6. Funston G, Van Melle M, Baun M-LL, et al. Variation in the initial assessment and investigation for ovarian cancer in symptomatic women: a systematic review of international guidelines. *BMC cancer* 2019;**19**(1):1028.
7. NHS UK. Diagnosis: Ovarian Cancer. Secondary Diagnosis: Ovarian Cancer 2020. https://www.nhs.uk/conditions/ovarian-cancer/diagnosis/.
8. Sundar S, Neal RD, Kehoe S. Diagnosis of ovarian cancer. *Bmj* 2015;**351**:h4443.
9. NICE. The guidelines manual: Process and methods [PMG6]. Secondary The guidelines manual: Process and methods [PMG6] 2012. https://www.nice.org.uk/process/pmg6/resources/how-nice-clinical-guidelines-are-developed-an-overview-for-stakeholders-the-public-and-the-nhs-2549708893/chapter/nice-clinical-guidelines.
10. Drescher CW, Nelson J, Peacock S, et al. Compliance of average-and intermediate-risk women to semiannual ovarian cancer screening. *Cancer Epidemiology and Prevention Biomarkers* 2004;**13**(4):600-06.
11. Gentry-Maharaj A, Sharma A, Burnell M, et al. Acceptance of transvaginal sonography by postmenopausal women participating in the United Kingdom Collaborative Trial of Ovarian Cancer Screening. *Ultrasound in obstetrics & gynecology* 2013;**41**(1):73-79.
12. Cowan Bennett C, Richards D. Patient acceptance of endovaginal ultrasound. *Ultrasound in Obstetrics and Gynecology* 2000;**15**(1):52-55.
13. Hollis G, Westbury C. When is best-worst best? A comparison of best-worst scaling, numeric estimation, and rating scales for collection of semantic norms. *Behavior research methods* 2018;**50**(1):115-33.
14. Czajkowski M, Hanley N, LaRiviere J. The effects of experience on preferences: theory and empirics for environmental public goods. *American Journal of Agricultural Economics* 2015;**97**(1):333-51.
15. Neuman T, Neuman E, Neuman S. Explorations of the effect of experience on preferences for a health-care service. *The Journal of Socio-Economics* 2010;**39**(3):407-19.
16. Flynn TN, Louviere JJ, Peters TJ, et al. Best–worst scaling: what it can do for health care research and how to do it. *Journal of health economics* 2007;**26**(1):171-89.
17. Mühlbacher AC, Kaczynski A, Zweifel P, et al. Experimental measurement of preferences in health and healthcare using best-worst scaling: an overview. *Health economics review* 2016;**6**(1):1-14.
18. Cheung KL, Wijnen BF, Hollin IL, et al. Using best–worst scaling to investigate preferences in health care. *Pharmacoeconomics* 2016;**34**(12):1195-209.

For peer review only - http://bmjopen.bmj.com/site/about/guidelines.xhtml

19. Louviere JJ, Flynn TN, Marley AAJ. *Best-worst scaling: Theory, methods and applications*: Cambridge University Press, 2015.
20. Hall R, Medina-Lara A, Hamilton W, et al. Attributes Used for Cancer Screening Discrete Choice Experiments: A Systematic Review. *The Patient-Patient-Centered Outcomes Research* 2021:1-17.
21. Louviere J, Lings I, Islam T, et al. An introduction to the application of (case 1) best–worst scaling in marketing research. *International journal of research in marketing* 2013;**30**(3):292-303.
22. Hauser DJ, Schwarz N. It'sa trap! Instructional manipulation checks prompt systematic thinking on "tricky" tasks. *Sage Open* 2015;**5**(2):2158244015584617.
23. Peer E, Brandimarte L, Samat S, et al. Beyond the Turk: Alternative platforms for crowdsourcing behavioral research. *Journal of Experimental Social Psychology* 2017;**70**:153-63.
24. Palan S, Schitter C. Prolific. ac—A subject pool for online experiments. *Journal of Behavioral and Experimental Finance* 2018;**17**:22-27.
25. NICE. Ovarian cancer: recognition and initial management. Secondary Ovarian cancer: recognition and initial management 2011. https://www.nice.org.uk/guidance/CG122.
26. Richardson DR, Oakes AH, Crossnohere NL, et al. Prioritizing the worries of AML patients: Quantifying patient experience using best–worst scaling. *Psycho-Oncology* 2021;**30**(7):1104-11.
27. Maximum difference scaling: improved measures of importance and preference for segmentation. Sawtooth software conference proceedings; 2003. Sawtooth Software, Inc. Fir St., Sequim, WA.
28. StataCorp L. Stata statistical software: Release 17 (2021). *College Station, TX: StataCorp LP* 2021.
29. Howard K, Salkeld G, Pignone M, et al. Preferences for CT colonography and colonoscopy as diagnostic tests for colorectal cancer: A discrete choice experiment. *Value in Health* 2011;**14**(8):1146-52.
30. De Bekker-Grob EW, Rose JM, Donkers B, et al. Men's preferences for prostate cancer screening: A discrete choice experiment. *British Journal of Cancer* 2013;**108**(3):533-41.
31. Mansfield C, Ekwueme DU, Tangka FKL, et al. Colorectal Cancer Screening: Preferences, Past Behavior, and Future Intentions. *Patient* 2018:1-13.
32. Miles A, Taylor SA, Evans RE, et al. Patient preferences for whole-body MRI or conventional staging pathways in lung and colorectal cancer: a discrete choice experiment. *European radiology* 2019;**29**(7):3889-900.
33. Fitch MI, Deane K, Howell D, et al. Women's experiences with ovarian cancer: reflections on being diagnosed. *Canadian Oncology Nursing Journal/Revue canadienne de soins infirmiers en oncologie* 2002;**12**(3):152-59.
34. Jelicic L, Brooker J, Shand L, et al. Experiences and health care preferences of women with ovarian cancer during the diagnosis phase. *Psycho-oncology* 2019;**28**(2):379-85.
35. Hol L, De Bekker-Grob EW, Van Dam L, et al. Preferences for colorectal cancer screening strategies: A discrete choice experiment. *British Journal of Cancer* 2010;**102**(6):972-80.
36. van Dam L, Hol L, Bekker-Grob EWd, et al. What determines individuals' preferences for colorectal cancer screening programmes? A discrete choice experiment. *European Journal of Cancer* 2010;**46**(1):150-59.
37. Salkeld G, Ryan M, Short L. The Veil of Experience: Do Consumers Prefer What They Know Best? *Health Economics* 2000;**9**(3):267-70.
38. Finn A, Louviere JJ. Determining the appropriate response to evidence of public concern: the case of food safety. *Journal of Public Policy & Marketing* 1992;**11**(2):12-25.
39. de Bekker-Grob EW, Donkers B, Jonker MF, et al. Sample size requirements for discrete-choice experiments in healthcare: a practical guide. *The Patient-Patient-Centered Outcomes Research* 2015;**8**(5):373-84.
40. Naik G, Ahmed H, Edwards AG. Communicating risk to patients and the public. *British Journal of General Practice* 2012;**62**(597):213-16.

For peer review only - http://bmjopen.bmj.com/site/about/guidelines.xhtml

41. Drummond M, Torbica A, Tarricone R. Should health technology assessment be more patient centric? If so, how?: Springer, 2020.

Figure captions

Figure 1: Example of a choice task

Figure 2: Best-worst scaling results. The distance between attributes is a spatial representation of the difference in relative importance between attributes on the latent importance scale.

Figure 3: Sub-group analysis results comparing importance scores between those previously tested for ovarian cancer (n=40) and those who have never been tested (n=110). Error bars represent 95% confidence intervals

Figure 4: Subgroup analysis results comparing importance scores between those who have previously undergone a transvaginal ultrasound for any medical reason (n=50) and those who have not (n=100). Error bars represent 95% confidence intervals

For peer review only - http://bmjopen.bmj.com/site/about/guidelines.xhtml

Figure 1: Example of a choice task

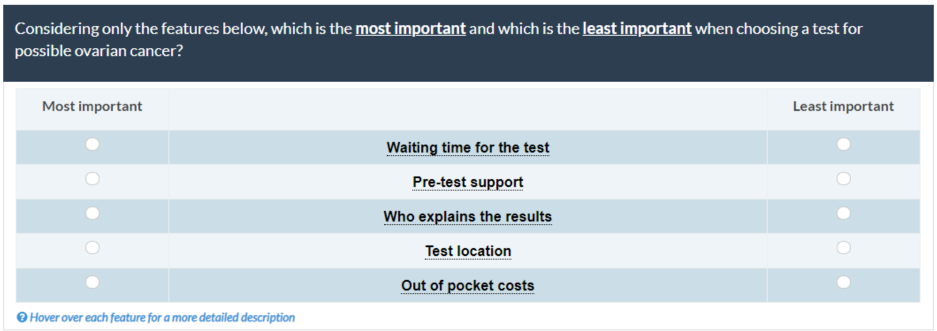

For peer review only

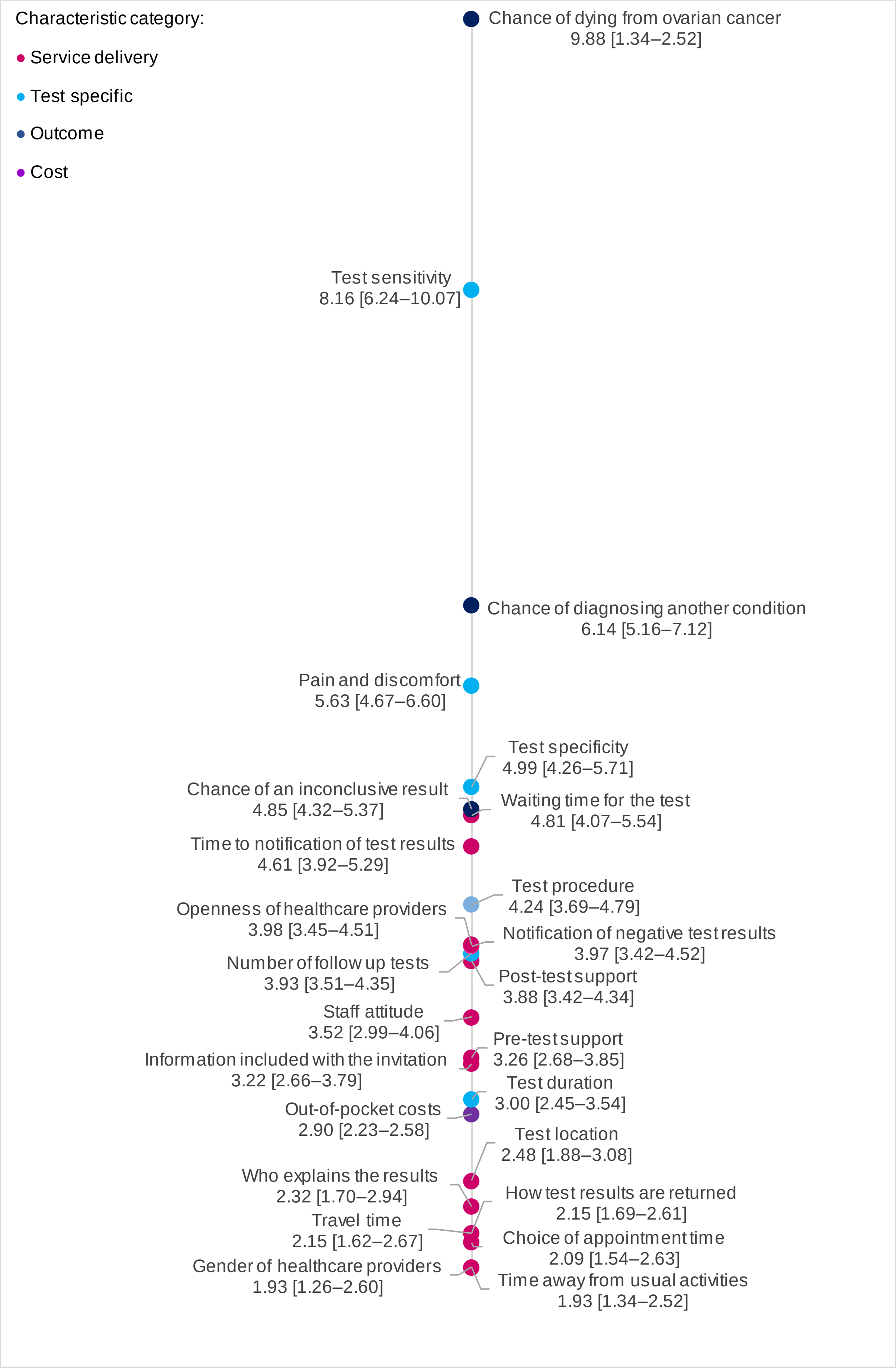
Characteristic category:
Service delivery
Test specific
Outcome
Cost
Chance of dying from ovarian cancer
9.88 [1.34–2.52]
Test sensitivity
8.16 [6.24–10.07]
Chance of diagnosing another condition
6.14 [5.16–7.12]
Pain and discomfort
5.63 [4.67–6.60]
Test specificity
4.99 [4.26–5.71]
Chance of an inconclusive result
4.85 [4.32–5.37]
Waiting time for the test
4.81 [4.07–5.54]
Time to notification of test results
4.61 [3.92–5.29]
Test procedure
4.24 [3.69–4.79]
Openness of healthcare providers
3.98 [3.45–4.51]
Notification of negative test results
3.97 [3.42–4.52]
Number of follow up tests
3.93 [3.51–4.35]
Post-test support
3.88 [3.42–4.34]
Staff attitude
3.52 [2.99–4.06]
Pre-test support
3.26 [2.68–3.85]
Information included with the invitation
3.22 [2.66–3.79]
Test duration
3.00 [2.45–3.54]
Out-of-pocket costs
2.90 [2.23–2.58]
Test location
2.48 [1.88–3.08]
Who explains the results
2.32 [1.70–2.94]
How test results are returned
2.15 [1.69–2.61]
Travel time
2.15 [1.62–2.67]
Choice of appointment time
2.09 [1.54–2.63]
Gender of healthcare providers
1.93 [1.26–2.60]
Time away from usual activities
1.93 [1.34–2.52]

Figure 3: Sub-group analysis results comparing relative importance scores between those previously tested for ovarian cancer (n=40) and those who have never been tested (n=110). Error bars represent 95% confidence intervals.

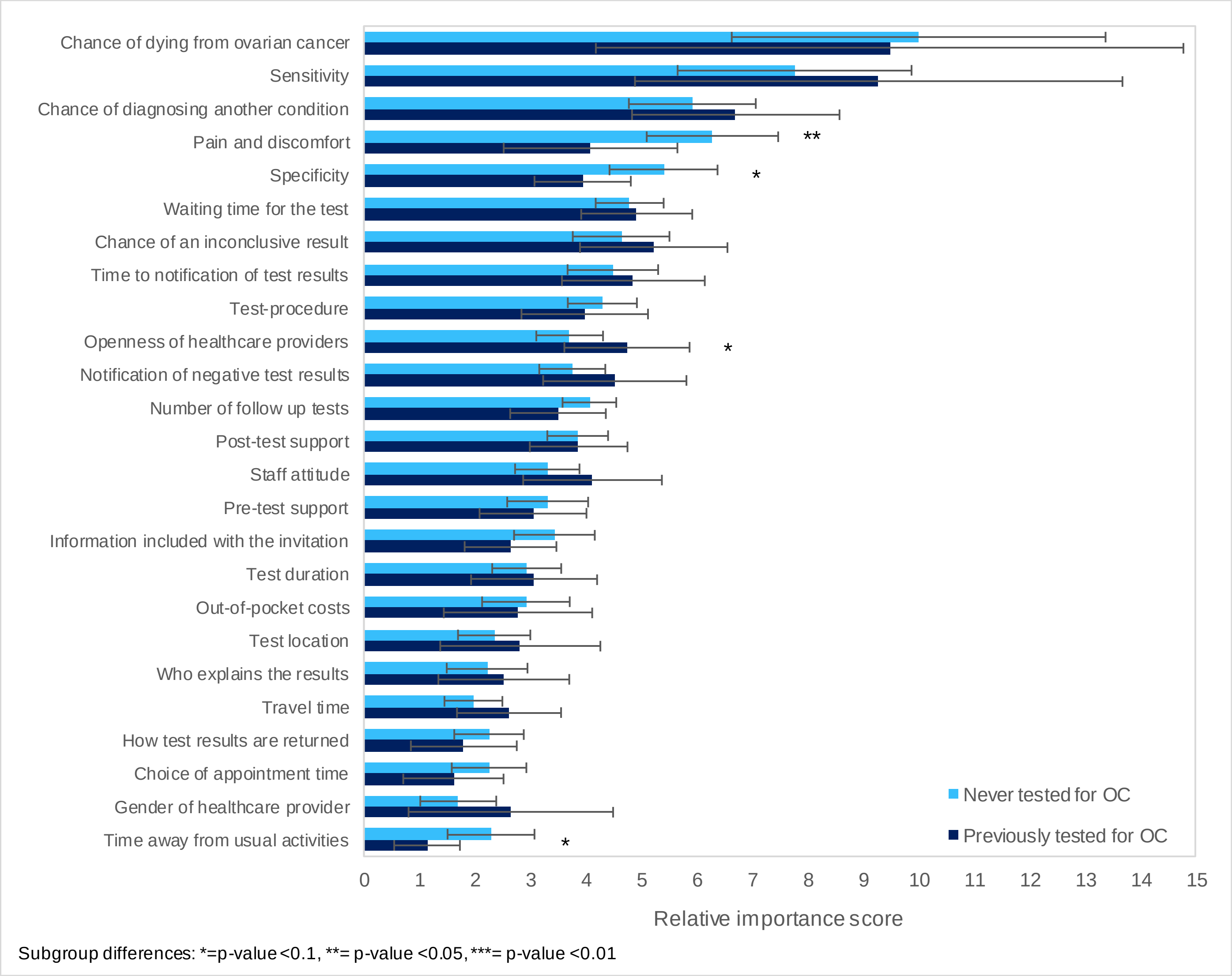

only

Figure 4: Subgroup analysis results comparing relative importance scores between those who have previously undergone a transvaginal ultrasound for any medical reason (n=50) and those who have not (n=100). Error bars represent 95% confidence intervals.

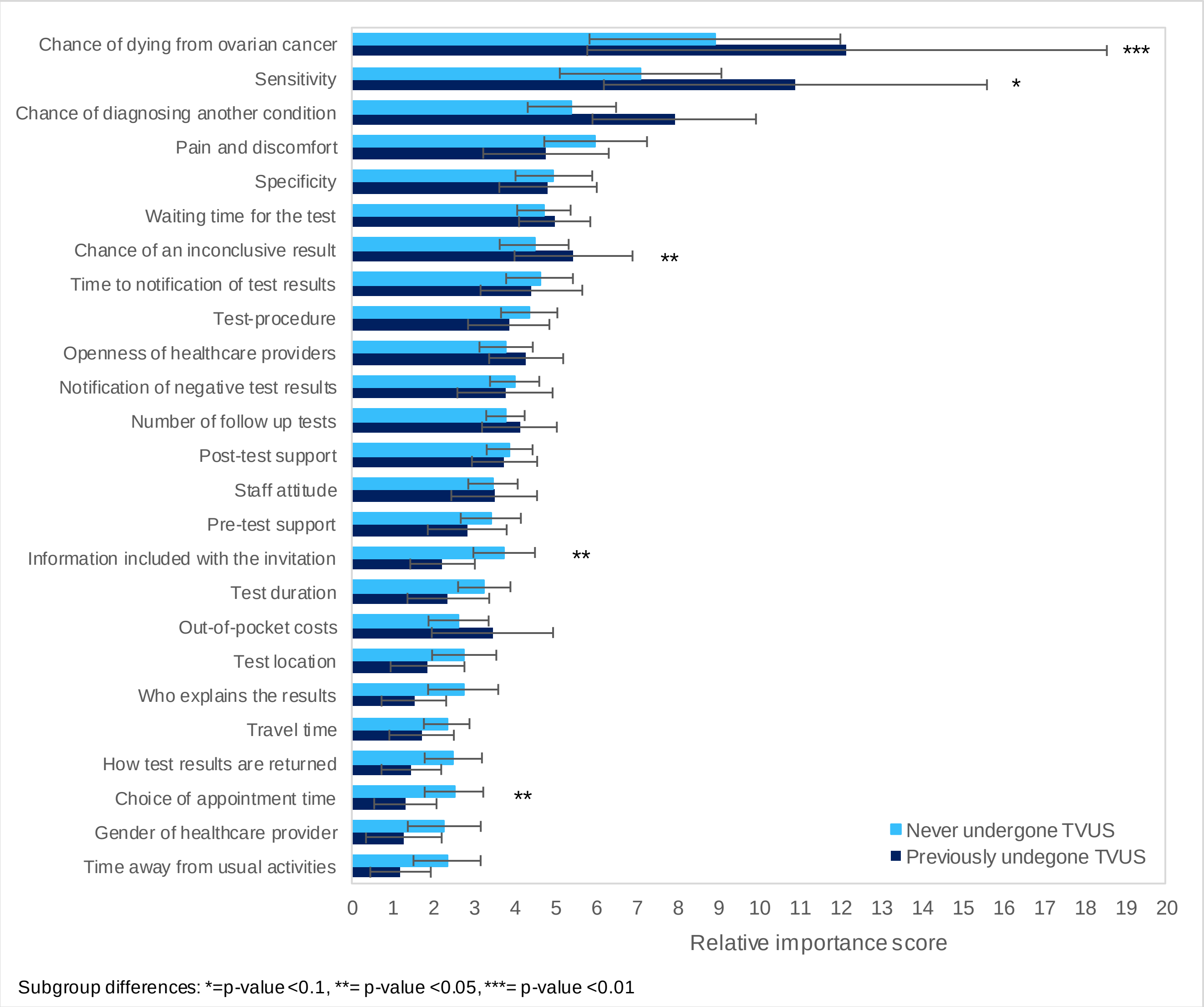

Appendix 1: Health-related characteristics of population

Characteristic	n (%)
**Self-reported overall health**	
Very good	18 (12)
Good	61 (41)
Fair	47 (31)
Poor	14 (9)
Very poor	4 (3)
Not reported	6 (4)
**Perceived risk of ovarian cancer**	
Very High	4 (3)
High	12 (8)
Average	83 (55)
Low	33 (22)
Very low	12 (8)
Not reported	6 (4)
**Ovarian cancer-related worry**	
A great deal	4 (3)
A lot	25 (17)
A moderate amount	43 (29)
A little	47 (31)
Not at all	26 (17)
Not reported	5 (3)
**Personal history of cancer**	17 (13)
**Knew someone who was diagnosed with ovarian cancer**	25 (17)
**Previously tested for ovarian cancer**	40 (27)
**Previously undergone a TVUS (any reason)**	50 (33)
**Cervical screening**	
Always attends/attended	75 (50)
Irregularly attends/attended	37 (25)
Never attended/stopped attending	37 (25)
Unknown	1 (1)
**How much confidence and trust do you have in GPs**	
A great deal/a lot	59 (39)
A moderate amount	53 (35)
A little	25 (17)
None at all	2 (1)
Unknown	11 (7)
**How much do you feel able to be involved in medical decisions**	
A great deal	12 (8)
A lot	22 (15)
A moderate amount	58 (39)
A little	34 (23)
Not at all	17 (11)
Unknown	7 (5)
**How much do you wish to be involved in medical decisions**	
A great deal	60 (40)
A lot	67 (45)
A moderate amount	19 (13)
A little	3 (2)
Not at all	1 (1)

For peer review only

Appendix 2: Full summary of raw best-worst scores, counting analysis and conditional logit results

Items	Most	Least	Most-Least	Counting: Standardised score	Counting: SD	Counting: 95% confidence interval	Conditional logit: Coefficient	Conditional logit: SE	Ranking
Sensitivity	410	133	277	0.308	0.60	0.21 – 0.40	1.44***	0.096	2
Chance of dying from ovarian cancer	504	162	342	0.380	0.70	0.26 – 0.49	1.63***	0.103	1
Choice of appointment time	122	316	-194	-0.216	0.47	-0.29 – (-0.15)	0.08	0.085	23
Who explains the results	150	306	-156	-0.173	0.52	-0.26 – (-0.09)	0.18*	0.087	20
Pain and discomfort	246	94	152	0.169	0.44	0.10 – 0.24	1.07***	0.084	4
Notification of negative test results	135	103	32	0.036	0.34	-0.02 – 0.09	0.72***	0.078	11
Chance of diagnosing another condition	289	107	182	0.202	0.45	0.13 – 0.27	1.16***	0.088	3
Pre-test support	111	147	-36	-0.040	0.33	-0.09 – 0.01	0.53***	0.078	15
Test-procedure	177	124	53	0.059	0.34	0.00 – 0.11	0.79***	0.081	9
Staff attitude	120	128	-8	-0.009	0.34	-0.06 – 0.05	0.60***	0.077	14
Post-test support	106	84	22	0.024	0.28	-0.02 – 0.07	0.70***	0.076	13
Time away from usual activities	138	358	-220	-0.244	0.56	-0.33 – (-0.15)	Ref	Ref	25
Specificity	239	125	114	0.127	0.42	0.06 – 0.19	0.95***	0.085	5
Travel time	107	292	-185	-0.206	0.44	-0.28 – (-0.14)	0.11	0.081	21
Time to notification of test results	194	112	82	0.091	0.37	0.03 – 0.15	0.87***	0.082	8
Openness of healthcare providers	134	101	33	0.037	0.32	-0.01 – 0.09	0.72***	0.78	10
Number of follow up tests	129	99	30	0.033	0.25	-0.00 – 0.07	0.71***	0.078	12
Chance of an inconclusive result	174	74	100	0.111	0.31	0.06 – 0.16	0.92***	0.077	6
Out-of-pocket costs	154	229	-75	-0.083	0.48	-0.16 – (-0.01)	0.41***	0.083	18
Gender of healthcare provider	182	401	-219	-0.243	0.67	-0.35 – (-0.14)	0.001	0.091	24
How test results are returned	104	290	-186	-0.207	0.40	-0.27 – (-0.14)	0.11	0.082	22
Test location	132	264	-132	-0.147	0.46	-0.22 – (-0.07)	0.25***	0.083	19
Test duration	95	161	-66	-0.073	0.32	-0.12 – (-0.02)	0.44***	0.077	17
Information included with the invitation	135	177	-42	-0.047	0.35	-0.10 – 0.01	0.51***	0.081	16
Waiting time for the test	213	113	100	0.111	0.37	0.05 – 0.17	0.91***	0.084	6

Key: ■ Five best scoring attributes in each round, ■ Five worst scoring attributes in each round. For the "least" column best and worst are inversed so worst scoring = rated least important most frequently

For peer review only

Appendix 3: Distribution of B-W scores across individuals. Demonstrating the degree of heterogeneity in preferences across individuals. Each attribute appeared within the survey six times meaning scores could range from 6 (always selected as most important) to -6 (always selected as least important).

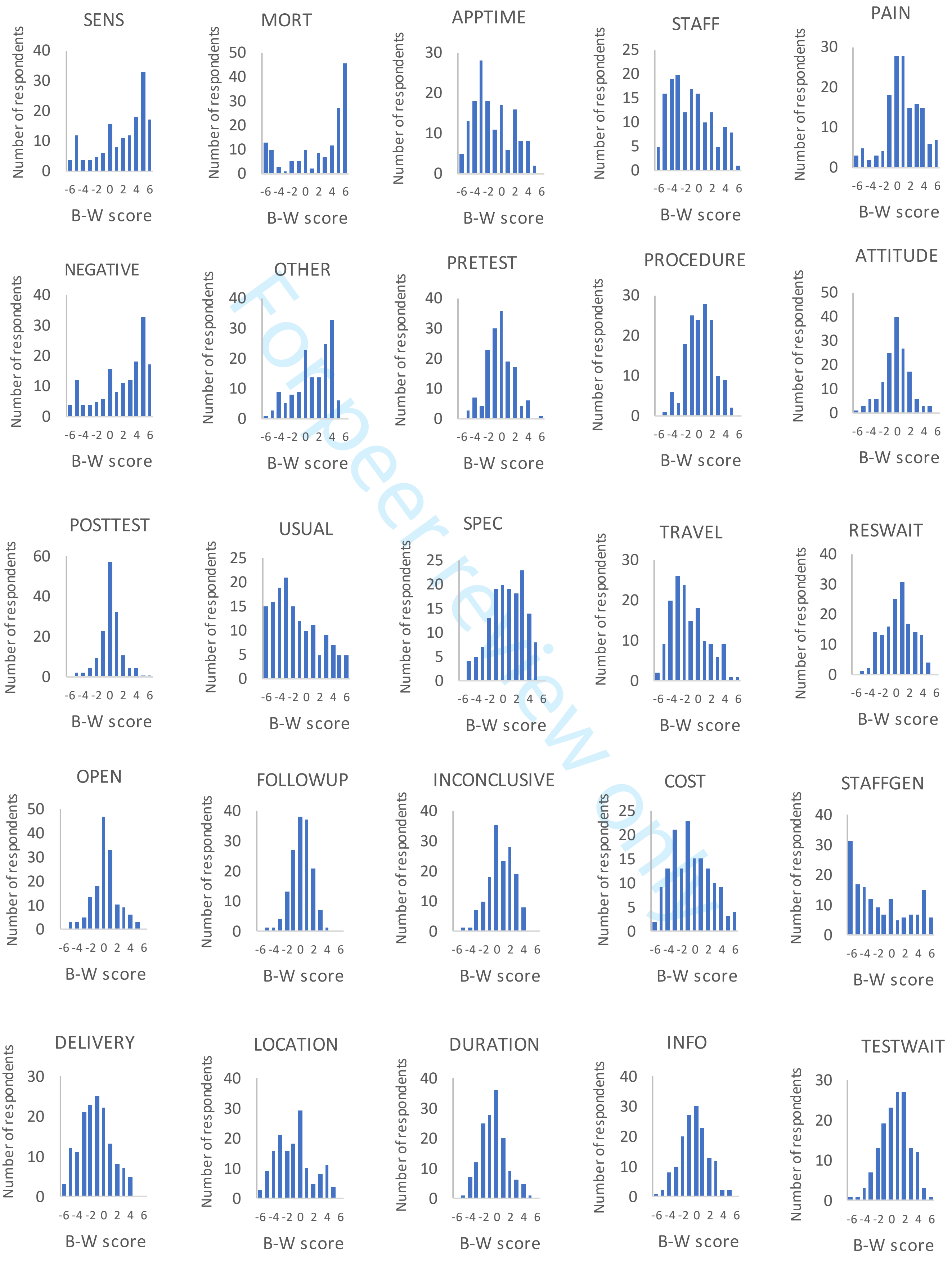

For peer review only

Appendix 4: Comparison of raw counting estimates and conditional logit estimates. Results were highly correlated

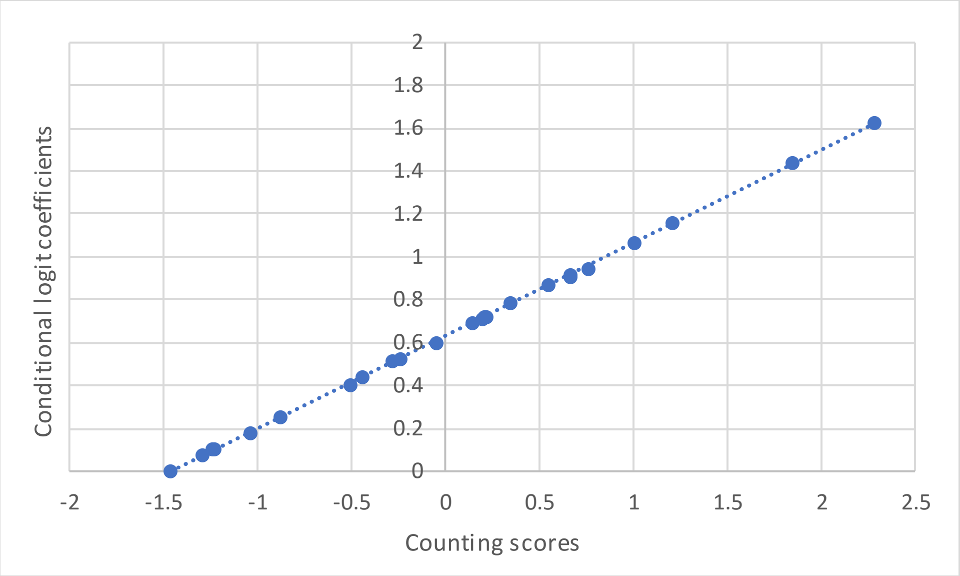

For peer review only

Appendix 5: Heteroscedastic logit estimates used to investigate differences in scale (i.e. error variance) between subgroups. A dummy variable representing previous test experience (either TVUS or OC testing) was included as an explanatory factor of the scale parameter. In both instances the scale term was not significant indicating observed differences in priorities between subgroups were not attributable to differences in error variance.

	OC test experience		TVUS experience	
	**Coefficient**	**SE**	**Coefficient**	**SE**
Items				
Sensitivity	1.63***	0.28	1.26***	0.35
Chance of dying from ovarian cancer	1.86***	0.30	1.47***	0.37
Choice of appointment time	0.13	0.17	0.07	0.14
Who explains the results	0.39*	0.21	0.29*	0.17
Pain and discomfort	1.13***	0.26	0.87***	0.27
Notification of negative test results	0.84***	0.19	0.68***	0.21
Chance of diagnosing another condition	1.30***	0.25	1.08***	0.26
Pre-test support	0.73***	0.18	0.58***	0.18
Test-procedure	1.06***	0.23	0.87***	0.24
Staff attitude	0.64***	0.20	0.56***	0.17
Post-test support	0.82***	0.19	0.68***	0.19
Time away from usual activities	Ref	Ref	Ref	Ref
Specificity	1.15***	0.24	0.22***	0.14
Travel time	0.23	0.19	0.86	0.23
Time to notification of test results	1.05***	0.22	0.64***	0.19
Openness of healthcare providers	0.74***	0.22	0.83***	0.22
Number of follow up tests	1.04***	0.21	0.85***	0.21
Chance of an inconclusive result	1.00***	0.21	0.41***	0.14
Out-of-pocket costs	0.44**	0.17	0.17***	0.18
Gender of healthcare provider	0.27	0.23	0.19	0.14
How test results are returned	0.21	0.20	0.15	0.14
Test location	0.18	0.18	0.26	0.15
Test duration	0.34**	0.16	0.44*	0.18
Information included with the invitation	0.58***	0.20	0.86**	0.22
Waiting time for the test	1.06***	0.21	0.22***	0.14
Scale factor				
Previous OC test (1=yes, 0=no)	-0.07	0.36		
Previous TVUS (1=yes, 0-no)			0.54*	0.32
Model statistics				
Log-likelihood	-3309.6			
Observations	31080		31080	
N	150		150	
Confidence levels: ***99%, ** 95%, *90%				

For peer review only

BMJ Open

Women's priorities towards ovarian cancer testing: A best-worst scaling study

Journal:	*BMJ Open*
Manuscript ID	bmjopen-2022-061625.R2
Article Type:	Original research
Date Submitted by the Author:	31-Jul-2022
Complete List of Authors:	Hall, Rebekah; University of Exeter Medical School, Health Economics Group<br>Medina-Lara, Antonieta; University of Exeter Medical School, Health Economics Group<br>Hamilton, Willie; University of Exeter Medical School, Primary Care Diagnostics<br>Spencer, Anne; University of Exeter Medical School, Health Economics
**Primary Subject Heading**:	Oncology
Secondary Subject Heading:	Health economics, Patient-centred medicine
Keywords:	Gynaecological oncology < GYNAECOLOGY, Health economics < HEALTH SERVICES ADMINISTRATION & MANAGEMENT, Gynaecological oncology < ONCOLOGY, Ultrasound < RADIOLOGY & IMAGING

SCHOLARONE™
Manuscripts

I, the Submitting Author has the right to grant and does grant on behalf of all authors of the Work (as defined in the below author licence), an exclusive licence and/or a non-exclusive licence for contributions from authors who are: i) UK Crown employees; ii) where BMJ has agreed a CC-BY licence shall apply, and/or iii) in accordance with the terms applicable for US Federal Government officers or employees acting as part of their official duties; on a worldwide, perpetual, irrevocable, royalty-free basis to BMJ Publishing Group Ltd ("BMJ") its licensees and where the relevant Journal is co-owned by BMJ to the co-owners of the Journal, to publish the Work in this journal and any other BMJ products and to exploit all rights, as set out in our licence.

The Submitting Author accepts and understands that any supply made under these terms is made by BMJ to the Submitting Author unless you are acting as an employee on behalf of your employer or a postgraduate student of an affiliated institution which is paying any applicable article publishing charge ("APC") for Open Access articles. Where the Submitting Author wishes to make the Work available on an Open Access basis (and intends to pay the relevant APC), the terms of reuse of such Open Access shall be governed by a Creative Commons licence – details of these licences and which Creative Commons licence will apply to this Work are set out in our licence referred to above.

Other than as permitted in any relevant BMJ Author's Self Archiving Policies, I confirm this Work has not been accepted for publication elsewhere, is not being considered for publication elsewhere and does not duplicate material already published. I confirm all authors consent to publication of this Work and authorise the granting of this licence.

For peer review only

Title: Women's priorities towards ovarian cancer testing: A best-worst scaling study

Rebekah Hall[a], Antonieta Medina-Lara[a], Willie Hamilton[b], Anne E Spencer[a]

[a] Health Economics Group, College of Medicine and Health, University of Exeter, Exeter, UK

[b] Primary Care Diagnostics, College of Medicine and Health, University of Exeter, Exeter, UK

Corresponding Author:

Rebekah Hall

Address: University of Exeter, St Luke's Campus, Heavitree, Exeter, EX1 2LU, UK

Email: rh591@exeter.ac.uk

Phone: +44 (0)1392 725565

ORCID ID:

Rebekah Hall: 0000-0003-1804-993X

Antonieta Medina-Lara: 0000-0001-7325-8246

Willie Hamilton: 0000-0003-1611-1373

Anne Spencer: 0000-0002-8163-3103

For peer review only

Word counts: Abstract: 262 Main body: 3305

Abstract:

Objective: To investigate the importance of key characteristics relating to diagnostic testing for ovarian cancer and to understand how previous test experience influences priorities.

Design: Case 1 best-worst scaling embedded in an online survey

Setting: Primary care diagnostic testing in England and Wales

For peer review only - http://bmjopen.bmj.com/site/about/guidelines.xhtml

Participants: 150 women with ovaries over 40 years old living in England and Wales.

Methods: We used best-worst scaling, a preference-based survey method, to elicit the relative importance of twenty-five characteristics relating to ovarian cancer testing following a systematic review. Responses were modelled using conditional logit regression. Sub-group analysis investigated variations based on testing history.

Main outcome measures: Relative importance scores

Results: “Chance of dying from ovarian cancer” (0.380, CI[0.26–0.49]) was the most important factor to respondents, closely followed by “test sensitivity” (0.308, CI[0.21–0.40]). In contrast, “time away from usual activities” (-0.244, CI[-0.33–(-0.15)]) and “gender of healthcare provider” (-0.243, CI[-0.35–(-0.14)]) were least important to respondents overall. Women who had previously undergone testing placed higher importance on certain characteristics including “openness of healthcare providers” and “chance of diagnosing another condition” at the expense of reduced emphasis on characteristics such as “pain and discomfort” and “time away from usual activities”.

Conclusions: The results clearly demonstrated items at the extreme, that were most and least important to women considering ovarian cancer testing. Differences in priorities by testing history demonstrate an experience effect, whereby preferences adapt over time based on evidence and experience. Acknowledging these differences helps to identify underlying barriers and facilitators for women with no test experience as well as shortcomings of current service based on women with experience.

Strengths and limitations:

- This study adds to a very limited evidence base of studies assessing priorities towards diagnostic testing for cancer and specifically ovarian cancer.
- Selection of included characteristics is based on a rigorous, published systematic review and utilised patient and public involvement to ensure the relevance to the target population

For peer review only

For peer review only - http://bmjopen.bmj.com/site/about/guidelines.xhtml

- Case 1 best worst scaling is ideal for establishing the relative importance of a large number of characteristics and has been proven to be easier to complete and more effective than alternative methods such as ranking or ratings tasks
- A key limitation of the study relates to the representativeness of the sample. Due to the recruitment method, the sample is not fully representative of the population in key demographics including ethnicity and age distribution
- The lack of discrimination between lower scoring attributes may be reflective of genuine priorities. However, it is also possible that choice task construction and sample size were contributing factors

1. Introduction

Ovarian cancer is the seventh most common cancer in women worldwide, with over 200,000 new cases and approximately 180,000 deaths annually.[1] Five-year survival rates for the disease are highly dependent on a number of factors including the patient's age, country of residence and tumour type.[2] Late-stage diagnosis contributes heavily to the high mortality rates associated with ovarian cancer and is an ongoing challenge globally.[3] For instance, in the UK, almost 60% of cases are diagnosed at stage III or IV where average five-year survival is just 26.9% and 13.4% respectively.[4] Improving diagnostic outcomes is multifaceted problem, however, delays in help-seeking upon symptom onset and access to timely testing have been identified as challenges to earlier diagnosis.[5]

National guidelines for the investigation of suspected ovarian cancer in symptomatic women vary substantially between countries.[6] Existing tests include the CA125 blood test and imaging tests, most commonly a transabdominal/transvaginal ultrasound (TVUS) but also Computed Tomography (CT) and Magnetic Resonance Imaging (MRI).[7] Variations in guidance represents uncertainty around the accuracy of existing test strategies, especially for the early investigation of symptoms. Evidence

For peer review only - http://bmjopen.bmj.com/site/about/guidelines.xhtml

evaluating the performance of diagnostic tests in a primary care setting is very limited.[8] Furthermore, for recommendations to be effective, clinical guidelines must also consider the preferences of those offered testing, particularly in healthcare systems emphasising shared-decision making.[9] Available tests differ substantially, not only in terms of performance but also service delivery and patient experience. Aspects of tests may be prioritised differently; for instance, patients may accept a lower levels of accuracy a less invasive test. Thus, it may be necessary to weight characteristics differently when considering the overall balance of benefits and harms.

To date, very little attention has been paid to the priorities of women facing ovarian cancer testing, particularly in a diagnostic setting. Existing studies usually focus on screening trials[10], single test modalities [11] or single aspects of acceptability, such as pain.[12] To address the current evidence gap, we aimed to elicit preferences for twenty-five key characteristics (‘items’) of ovarian cancer testing, using best-worst scaling (BWS). This is a stated preference technique that has been demonstrated to provide higher predictive values than ranking or rating methods whilst being less cognitively demanding.[13] Understanding the importance of key characteristics allows aspects of greatest importance to be given increased salience in future decisions about testing, particularly in guideline revision.

Previous studies have demonstrated views may differ based on previous experience of the health event. We therefore examined the relationship between priorities around ovarian cancer testing and test experience.[14 15]

2. Methods

2.1 Patient and public involvement in the research

The survey was shared and discussed with the Cancer Research UK funded CanTest PPI lead, Margaret Johnson. Amendments in survey wording were made as a result of discussions.

2.2 Best worst scaling

For peer review only - http://bmjopen.bmj.com/site/about/guidelines.xhtml

We used Case 1 best-worst scaling (BWS) method to identify women's preferences for characteristics associated with diagnostic testing for ovarian cancer. This type of BWS aims to assess the relative importance of, or preference for, items based on the underlying principles of random utility theory.[16] BWS was initially developed in marketing but has been increasingly utilised in healthcare research for explorations of patient and stakeholder preferences.[16-18] In particular, Case 1 BWS has been demonstrated to be an effective alternative to traditionally used ranking or ratings tasks when considering patient priorities.[19]

During BWS tasks, participants respond to a series of choice tasks presenting a subset of items and asked to select the "best" or "most important" and "worst" or "least important" item. Simultaneously examining items selected as "most" and "least" important provides greater information than examining the most important item alone. Analysing responses to choice tasks allows the underlying relative importance of items to be inferred and a ranking of included items to be established[19].

2.3 Identification of relevant items

Results demonstrate the importance of an item, relative to the other included item. It is therefore important to use a rigorous selection process to ensure the most salient characteristics are included in the experiment. We performed a systematic review of the preference-based literature to identify characteristics relating to cancer testing.[20] Potential characteristics were then narrowed down by the authors based on relevance to ovarian cancer testing in symptomatic patients using an iterative Delphi method process where exclusion required full agreement of the research team. In total, twenty-five characteristics were selected for inclusion (Table 1).

For peer review only - http://bmjopen.bmj.com/site/about/guidelines.xhtml

1 Table 1: Characteristics included in the BWS study (wording and descriptions are identical to those
2 shown to respondents during the survey)

	Characteristic and definition
1	**Test sensitivity**<br>*Chance that the test will miss cancer in a patient who actually does have the disease (false-negative result)*
2	**Chance of dying from ovarian cancer**<br>*How much having the test decreases the chance of dying from ovarian cancer*
3	**Choice of appointment time**<br>*Whether a person can choose an appointment time or if the appointment time is assigned by the health care provider*
4	**Who explains the results**<br>*Type of health care provider who explains the test results e.g. Nurse, doctor etc.*
5	**Pain and discomfort**<br>*The level of pain and/or discomfort experienced during and after the test*
6	**Notification of negative test results**<br>*Whether you are contacted if your results are normal*
7	**Chance of diagnosing another condition**<br>*If symptoms are not caused by cancer, the chance the test can identify other reason for the symptoms*
8	**Pre-test support**<br>*Level of support received before having the test describing what will happen during the test and what the results might show*
9	**Test procedure**<br>*What having the test will involve. For ovarian cancer this could be a blood test or an transvaginal ultrasound (internal ultrasound of the reproductive organs)*
10	**Staff attitude**<br>*How the health care provider treats you while conducting the test*
11	**Post-test support**<br>*Level of support received after getting the results of the test relating to the meaning of your results and what will happen next*
12	**Time away from usual activities**<br>*The total amount of time spent having the test instead of doing your usual daily activities*
13	**Test specificity**<br>*Chance of unnecessary further invasive testing (false-positive result)*
14	**Travel time**<br>*The total amount of time spent travelling to and from the test*
15	**Time to notification of results**<br>*The length of time it takes to hear the results after having the test*
16	**Openness of health care providers**<br>*How open health care providers are with their thoughts about the cause of your symptoms and the tests they recommend*
17	**Number of follow up tests**<br>*How many additional tests are needed to confirm a diagnosis*
18	**Chance of an inconclusive result**<br>*The chance the results are unclear and the test would need to be repeated after a waiting period*
19	**Out of pocket costs**<br>*How much it will personally cost a person to have the test e.g. Travel costs, childcare costs, time off work etc. The cost of the test is covered by the NHS*

For peer review only

20	**Gender of healthcare provider**<br>*Gender of the staff member giving you the test*
21	**How test results are returned**<br>*e.g. in person, phone, letter*
22	**Test location**<br>*Where the test takes place*
23	**Test duration**<br>*The length of time spent having the test*
24	**Information included with the invitation**<br>*The level and type of information received about the test*
25	**Waiting time for the test**<br>*How long a person has to wait to have the test after being referred by their GP*

2.4 BWS task construction

A balanced incomplete block design (BIBD) generated using SAS 9.4 was used to construct the BWS choice sets for the 25 items. BIBD designs ensure each item appears equally often, and pairwise comparisons between each of the items occurs equally across the design.[21] A final design (d-efficiency of 83.3%) consisting of 30 choice tasks each with five items was selected based on a trade-off between survey complexity and design efficiency. Each item appeared six times across the choice tasks and coappeared with remaining attributes once throughout the experiment. The position of the item within choice tasks was optimised such that items were listed in 1st-5th position equally and the order of tasks was randomised across participants to control for any order effects. Each participant completed all thirty tasks. An example of a BWS task is shown in Figure 1.

2.5 Questionnaire

The questionnaire was developed and collected using LimeSurvey, an online survey platform. The survey consisted of four stages:

i. Socio-demographic questions e.g. age, education, employment status
ii. Best-worst scaling questions (including a warm-up task)
iii. Task feedback questions e.g. task difficulty

For peer review only

For peer review only - http://bmjopen.bmj.com/site/about/guidelines.xhtml

iv. Background questions relating to health-related characteristics e.g. self-reported health, testing history, current and desired in medical decisions

Question framing and answer categories for stages i and iii-iv were copied or adapted from established national surveys. Early piloting with five women suggested the survey would take 30 minutes to complete. Given the length of the survey and online administration method, three attention checks following the instructional manipulation format were embedded (e.g. "Select 'very important' to indicate you are paying attention").[22] Respondents who failed all three attention checks were removed from the analysis. A full version of the questionnaire is available upon request.

2.6 Participants

A sample size of 150 women was estimated to be required. Participants were recruited via Prolific, an online platform for researchers conducting social science experiments. Results from this platform have been shown to be of comparable or better quality than university research lab registers and have been used widely within hundreds of published studies across disciplines. [23 24] Our study focused on the priorities of women over 40 (no upper limit), the group most likely to be offered testing.[25] Participation was also limited to those living in England and Wales: no other limitations were applied. The study was approved by the University of Exeter UEBS-REC (eUEBS003725). Respondents completed an electronic consent form before completing any questions.

2.7 Statistical analysis

Descriptive statistics relating to sociodemographic and health-related characteristics of the sample were summarised.

Best-worst responses were initially analysed using the counting approach; whereby the number of times an item was picked as 'least important' is subtracted from the number of times it was chosen as 'most important'. Each item appeared 6 times across all tasks, meaning best-worst scores could range from -6 to +6 at the individual level. Individual scores were aggregated and standardised to

For peer review only - http://bmjopen.bmj.com/site/about/guidelines.xhtml

calculate an overall mean score for the sample ranging from -1 to +1[1]. A score close to +1 indicates an item is highly influential, whereas item with scores near to -1 demonstrate much less relevance.

Next, conditional logit regression using dummy-coded items was used to model responses. “Time away from usual activities” was identified as the least important attribute during counting analysis and was omitted from the model and used as the reference item. As a result, all parameters were expected to be positive. To aid interpretation, we rescaled conditional logit coefficients using standardised ratio scores where all scores sum to 100.[26 27] Confidence intervals for relative importance scores were estimated using the delta method. All analyses were performed using Stata 17.[28]

In both the counting and conditional logit analysis, scores relate to the relative importance of attributes (i.e. relative importance scores). In other words, differences between scores are meaningful whereas absolute values are not.

2.7.1 Subgroup analysis

Subgroup analysis was performed to explore the relationship between previous testing and preferences for future testing. Experience was captured by two questions that asked whether women had previously: 1) undergone testing for ovarian cancer or 2) received a transvaginal ultrasound for any reason. Subgroup analysis was based on conditional logit results and differences in relative importance scores between groups were tested using unpaired T-tests. Finally, heteroscedastic logit models were estimated to investigate whether differences between subgroup were attributable to scale differences (i.e. differences in error variance between subgroups) or due to a genuine difference in preferences.

3 Results

[1]Scores were standardised using the following equation: population-level best-worst score/(number of times item appeared x total sample size).

For peer review only - http://bmjopen.bmj.com/site/about/guidelines.xhtml

In total 159 women responded to the survey. The average response time for the questionnaire was 29 mins 51 seconds. Four submissions were incomplete, two were removed due to failing all attention checks, and a further three responses were removed due to incorrect completion of the best-worst section of the survey, resulting in 150 responses for the final analysis. Respondents varied substantially in how difficult they found the best-worst portion of the questionnaire with 42% (63/150) reporting it easy/very easy but 38% (57/150) finding it difficult/very difficult. There were no significant differences in BWS responses between those who found the task difficult versus those who did not.

3.1 Demographics

The demographics of the sample are presented in Table 2. The age ranged from 40 to 87 years old with a mean of 51.4 (SD=9.1). Most participants were white (120/150; 80%), married/in a relationship (97/150; 65%) and employed (78/150; 52%).

Most women perceived their risk of cancer as low-average (128/150; 85%) and ovarian cancer-related anxiety was generally low-moderate amongst respondents (116/150; 77%). Overall, fifty women (33%) reported previously undergoing a TVUS for any reason. Forty (27%) women reported being previously tested for ovarian cancer, with CA125 blood test being the most common test.

Crucially, when asked, 127/150 women (89%) stated they wished for a great deal/a lot of involvement in decisions relation to their own care but only 34/150 (23%) currently felt this was achieved, with 17/150 (11%) respondents felt unable to be involved in medical decisions at all. Further details are found in Appendix 1.

Table 2: Descriptive characteristics: sociodemographic

Characteristic	n (%)

For peer review only - http://bmjopen.bmj.com/site/about/guidelines.xhtml

**Age**	
Mean (SD)	51.4 (9)
Range	40-87
**Ethnicity**	
White	120 (80)
Asian	8 (5)
Black	3 (2)
Mixed	3 (2)
Other	9 (6)
Not reported	7 (4)
**Number of children, mean (SD)**	1.3 (1)
**Relationship status**	
Married	75 (50)
In a relationship	22 (15)
Single	19 (13)
Divorced/separated	26 (17)
Widowed	6 (4)
Not reported	2 (1)
**Level of education**	
GCSE	37 (25)
A-Level/ College	25 (17)
Undergraduate	41 (27)
Post-graduate	35 (23)
No qualifications	1 (1)
Other	9 (6)
Not reported	2 (1)
**Employment status**	
Full-time	47 (31)
Part-time	32 (21)
Self-employed	23 (15)
Not employed	11 (7)
Retired	14 (9)
Other	18 (12)
Not reported	5 (3)

3.2 Best-worst results

3.2.1 Counting analysis

BWS results are presented in Appendix 2. Scores were bound between -1 and 1. Scores tending toward the extremes of the scale would imply homogeneity across respondents and consistency between responses across questions on an individual level. Importance scores ranged from -0.224 to 0.380,

For peer review only - http://bmjopen.bmj.com/site/about/guidelines.xhtml

suggesting high levels of heterogeneity in preferences regarding test characteristics across respondents (see Appendix 3).

Overall, "*Chance of dying from ovarian cancer*" (0.380, CI[0.26–0.49]) was most important to women when considering ovarian cancer testing, followed by "*Test sensitivity*" (0.308, CI[0.21–0.40]). Conversely, "*Time away from usual activities*" (-0.244, CI[-0.33–(-0.15)]) and "*Gender of healthcare provider*" (-0.243, CI[-0.35–(-0.14)]) were considered least important to women when facing diagnostic testing and were statistically indistinguishable from each other.

3.2.2 Conditional logit analysis

The results for the conditional logit model are shown in Appendix 2. The order of importance remained consistent across the two analysis methods and the estimates were highly correlated (Appendix 4).

All items had a positive coefficient and most were statistically significant at the 95% level, confirming the relative importance of all attributes compared to "*time away from usual activities*". Non-significant items were those with the lowest importance (e.g. "*gender of healthcare providers*" and "*how test results are returned*"), suggesting a clustering effect towards the bottom of the importance scale.

Figure 2 shows the relative importance scores associated with the conditional logit estimates. The distance between each item is a spatial visualisation of differences in relative importance. Importance scores ranged from a maximum of 9.88 [95% CI: 7.04—12.72] for "Chance of dying of ovarian cancer" to a minimum of 1.93 [95% CI:1.34—2.52] for "Time away from usual activities" and Gender of healthcare providers. Indicating "chance of dying from ovarian cancer" was approximately five times more important to respondents. The most important characteristics to respondents were clear and distinct: however, spatial visualisation demonstrated grouping of attributes towards the centre and bottom of the scale. Groupings were distinct to other items but differences in importance between items within clusters is less distinguishable. In general, results demonstrate a clear prioritisation of

For peer review only - http://bmjopen.bmj.com/site/about/guidelines.xhtml

outcome (dark blue dots) and test specific characteristics (light blue dots) whereas service delivery characteristics (pink dots) were consistently less important to respondents.

3.2.3 Subgroup analysis

Women previously tested for ovarian cancer placed significantly lower importance on "*pain and discomfort*" in comparison to test-naïve individuals. There was also evidence to suggest "*time away from usual activities*" and "*test specificity*" were less prioritised by previously tested women whilst "*openness of healthcare providers*" and "*test specificity*" appeared marginally more important to those with test experience (Figure 3).

For women who had previously undergone a TVUS, "*chance of diagnosing another condition*" and "*chance of dying from ovarian cancer*" were significantly more important compared to those with no test experience (Figure 4). Alternatively, women who had never been tested appeared to place higher value on "*information included with the invitation*" and "*choice of appointment time*", although the overall importance of such attributes remained relatively low.

In both instances, results from heteroscedastic logit models demonstrated no differences in scale between subgroups indicating observed differences were attributable to differences in priorities as opposed to differences in response variance between groups with different prior experience (Appendix 5).

4 Discussion

4.1 Summary of main findings

This is the first study to investigate the priorities of women relating to diagnostic testing for ovarian cancer. The results of this study highlight the importance yet current neglect of incorporating the preferences of patients into medical testing decisions. When asked, almost 90% (134/150) of respondents wanted heavy involvement in medical decisions: however, less than a quarter of women currently felt able to do so.

For peer review only - http://bmjopen.bmj.com/site/about/guidelines.xhtml

The results are particularly useful in identifying items at either extreme of the scale, clearly demonstrating the characteristics that are most and least important to women considering ovarian cancer. Low concurrence between items and high levels of heterogeneity meant discrimination between mid-range items was less clear. Overall, "*chance of dying from ovarian cancer*", "*test sensitivity*" and "*chance of diagnosing another condition*" were the most important characteristics when considering testing for ovarian cancer. Oppositely, "*time away from usual activities*" and "*gender of healthcare provider*" were the least important factors. The priorities of previously tested women were generally similar to women who had never undergone testing, but there were a few key differences between these two groups suggesting experience is an important determinant of priorities.

4.2 Results in the Context of Published Literature

The prioritisation of outcome and test-specific characteristics and the relative lack of importance placed on service delivery characteristics is reflective of findings from the wider literature examining preferences towards cancer screening and diagnosis.[20 29-32] The relative importance of "*openness of healthcare providers"* is echoed by qualitative research exploring the diagnostic experiences of women with ovarian cancer where shortcomings in doctor-patient communication was a recurring theme. In particular, patients raised concerns about doctors' willingness and ability to openly communicate and share information.[33 34] Decreased importance of particular characteristics to tested women may suggest these aspects of testing are already being achieved/acceptable within the current system. For example, the decreased importance of "*pain and discomfort*" for both women who have been tested for ovarian cancer and women who have previously received an ultrasound (NS) is expected given the finding that almost 80% of women who had previously undergone a transvaginal ultrasound experienced little to no pain.[12]

Existing studies examining differences in preferences towards cancer testing between previously tested and untested individuals have typically focused on colorectal cancer screening, but also found

For peer review only - http://bmjopen.bmj.com/site/about/guidelines.xhtml

evidence of statistically different preferences between the two groups.[35 36] Hol, et al. [35] found previously screened individuals displayed stronger and more positive preferences towards different screening modalities and shorter screening intervals than unscreened counterparts. In a similar study, van Dam, et al. [36] found limited differences between people with and without screening experience (mortality risk reduction only), however, preferences across previously screened individuals differed significantly based on the particular screening modality received. These results are evidence of an experience effect, via "status quo bias", where individuals place a higher value on goods or services that are more familiar.[37]

4.3 Strengths and Limitations

Best-worst scaling provides a straightforward method for capturing the priorities of women. The method is preferable to ranking/rating tasks due to its ability to measure the importance of large numbers of items whilst limiting complexity and cognitive burden.[38]

The survey length and high number of items represent a potential limitation. In total, 38% of respondents reported the task as difficult/very difficult. Despite this, drop-out rates were low (3% (4/150) of people began the survey but did not complete) and there were no differences between responses according to reported task difficulty.

Poor discrimination between lower scoring items may be reflective of genuine equality in priorities, however, it is also feasible that the choice task construction was a contributing factor. For example, the ability to detect differences will be affected by the number of pairwise comparisons between attributes. Selection of a BIBD with more than one pairwise comparison between each item may have increased the explanatory power of the study, of course this requires a trade-off with survey length and sample size requirements where blocking is required. Similarly, a larger sample size may have increased the explanatory power and also allowed for more complex statistical investigation of

For peer review only - http://bmjopen.bmj.com/site/about/guidelines.xhtml

preference heterogeneity. To our knowledge, there is currently no accepted guidance on sample size requirements within object case BWS, although theories from the closely related methods, such as discrete choice experiments may be transferable.[39]

Finally, due to the recruitment method, the sample is not fully representative of the population in key demographics including ethnicity and age distribution. Further research is needed to understand whether results are generalisable to a wider population.

4.4 Implications for Practice and Future Research

The findings of this study offer useful insights into potential barriers and facilitators of undergoing testing in a timely manner. Interestingly, characteristics involving an element of risk or uncertainty dominated the top-ranking positions. However, how to best explain complex aspects of test performance such as sensitivity and specificity to patients is a clear challenge.[40]

Differences in importance between women with and without test experience suggest that priorities are continuously adapted based on evidence and experience gained over time. When considering policy decisions, it is therefore important to carefully consider whose views should be prioritised - a longstanding debate within the field of health technology assessment.[41] Arguably, it is important to consider both perspectives; priorities of women with test experience help to identify unmet or inadequate aspects of current service provision, whereas preconceived views of testing-naïve women may reveal underlying barriers and facilitators of testing since the initial decision to undergo testing is based on these pre-existing judgements. In both instances, mismatches in priorities and practice may lead to delays in seeking help or testing for future symptoms.

High levels of heterogeneity between individuals highlight the importance of a personalised approach to patient interactions throughout the diagnostic process. Patients are likely to have different concerns and priorities during this time, however, the importance of patient-input and shared

For peer review only - http://bmjopen.bmj.com/site/about/guidelines.xhtml

decision-making appears to be less prioritised in diagnostic settings compared to decisions regarding cancer screening and subsequent treatment where preferences have been studied more extensively.

5. Conclusion

Preferences towards diagnostic testing have been underexplored to date. Understanding what matters most to patients may reduce anxiety around testing, facilitate earlier help-seeking behaviour and improve patient satisfaction. This study highlights that test sensitivity and mortality impacts are the most important factors to patients facing ovarian cancer testing. However, results varied significantly across individuals demonstrating the need for an individualised approach to consultations regarding diagnostic care. Our results can help inform policies and diagnostic guidelines designed to encourage earlier help-seeking behaviour as well as help to evaluate the patient-friendliness of emerging test strategies for suspected ovarian cancer.

Conflicts of interest: No conflicts of interest

Funding statement: This research arises from the CanTest Collaborative, which is funded by Cancer Research UK (reference number: C8640/A23385), of which Rebekah Hall is a funded PhD student, Willie Hamilton is director and Anne E Spencer is a senior faculty member.

Ethical approval: This study was approved by the University of Exeter UEBS-REC in July 2020. Reference number: eUEBS003725.

Author contributions: All authors contributed to the study conception and design. RH and AM-L conducted data analysis. RH prepared the first draft of the manuscript and AS, AM-L and WH edited and approved the final version.

Acknowledgements: The authors thank Margaret Johnson, CanTest PPI Panel lead, for providing feedback during the development of this study.

For peer review only - http://bmjopen.bmj.com/site/about/guidelines.xhtml

Data sharing statement: Anonymised survey data and analysis code are available from the corresponding over upon reasonable request.

References

1. Reid BM, Permuth JB, Sellers TA. Epidemiology of ovarian cancer: a review. *Cancer biology & medicine* 2017;**14**(1):9.
2. Matz M, Coleman MP, Carreira H, et al. Worldwide comparison of ovarian cancer survival: Histological group and stage at diagnosis (CONCORD-2). *Gynecologic oncology* 2017;**144**(2):396-404.
3. Bhatla N, Jones A. THE WORLD OVARIAN CANCER COALITION ATLAS. 2018.
4. Cancer Research UK. Ovarian Cancer Statistics Secondary Ovarian Cancer Statistics 2019. https://www.cancerresearchuk.org/health-professional/cancer-statistics/statistics-by-cancer-type/ovarian-cancer#heading-Two.
5. Target Ovarian Cancer. Pathfinder 2016. Transforming futures for women with ovarian cancer, 2016.
6. Funston G, Van Melle M, Baun M-LL, et al. Variation in the initial assessment and investigation for ovarian cancer in symptomatic women: a systematic review of international guidelines. *BMC cancer* 2019;**19**(1):1028.
7. NHS UK. Diagnosis: Ovarian Cancer. Secondary Diagnosis: Ovarian Cancer 2020. https://www.nhs.uk/conditions/ovarian-cancer/diagnosis/.
8. Sundar S, Neal RD, Kehoe S. Diagnosis of ovarian cancer. *Bmj* 2015;**351**:h4443.
9. NICE. The guidelines manual: Process and methods [PMG6]. Secondary The guidelines manual: Process and methods [PMG6] 2012. https://www.nice.org.uk/process/pmg6/resources/how-nice-clinical-guidelines-are-developed-an-overview-for-stakeholders-the-public-and-the-nhs-2549708893/chapter/nice-clinical-guidelines.
10. Drescher CW, Nelson J, Peacock S, et al. Compliance of average-and intermediate-risk women to semiannual ovarian cancer screening. *Cancer Epidemiology and Prevention Biomarkers* 2004;**13**(4):600-06.
11. Gentry-Maharaj A, Sharma A, Burnell M, et al. Acceptance of transvaginal sonography by postmenopausal women participating in the United Kingdom Collaborative Trial of Ovarian Cancer Screening. *Ultrasound in obstetrics & gynecology* 2013;**41**(1):73-79.
12. Cowan Bennett C, Richards D. Patient acceptance of endovaginal ultrasound. *Ultrasound in Obstetrics and Gynecology* 2000;**15**(1):52-55.
13. Hollis G, Westbury C. When is best-worst best? A comparison of best-worst scaling, numeric estimation, and rating scales for collection of semantic norms. *Behavior research methods* 2018;**50**(1):115-33.
14. Czajkowski M, Hanley N, LaRiviere J. The effects of experience on preferences: theory and empirics for environmental public goods. *American Journal of Agricultural Economics* 2015;**97**(1):333-51.
15. Neuman T, Neuman E, Neuman S. Explorations of the effect of experience on preferences for a health-care service. *The Journal of Socio-Economics* 2010;**39**(3):407-19.
16. Flynn TN, Louviere JJ, Peters TJ, et al. Best–worst scaling: what it can do for health care research and how to do it. *Journal of health economics* 2007;**26**(1):171-89.
17. Mühlbacher AC, Kaczynski A, Zweifel P, et al. Experimental measurement of preferences in health and healthcare using best-worst scaling: an overview. *Health economics review* 2016;**6**(1):1-14.
18. Cheung KL, Wijnen BF, Hollin IL, et al. Using best–worst scaling to investigate preferences in health care. *Pharmacoeconomics* 2016;**34**(12):1195-209.

For peer review only - http://bmjopen.bmj.com/site/about/guidelines.xhtml

19. Louviere JJ, Flynn TN, Marley AAJ. *Best-worst scaling: Theory, methods and applications*: Cambridge University Press, 2015.

20. Hall R, Medina-Lara A, Hamilton W, et al. Attributes Used for Cancer Screening Discrete Choice Experiments: A Systematic Review. *The Patient-Patient-Centered Outcomes Research* 2021:1-17.

21. Louviere J, Lings I, Islam T, et al. An introduction to the application of (case 1) best–worst scaling in marketing research. *International journal of research in marketing* 2013;**30**(3):292-303.

22. Hauser DJ, Schwarz N. It'sa trap! Instructional manipulation checks prompt systematic thinking on "tricky" tasks. *Sage Open* 2015;**5**(2):2158244015584617.

23. Peer E, Brandimarte L, Samat S, et al. Beyond the Turk: Alternative platforms for crowdsourcing behavioral research. *Journal of Experimental Social Psychology* 2017;**70**:153-63.

24. Palan S, Schitter C. Prolific. ac—A subject pool for online experiments. *Journal of Behavioral and Experimental Finance* 2018;**17**:22-27.

25. NICE. Ovarian cancer: recognition and initial management. Secondary Ovarian cancer: recognition and initial management 2011. https://www.nice.org.uk/guidance/CG122.

26. Richardson DR, Oakes AH, Crossnohere NL, et al. Prioritizing the worries of AML patients: Quantifying patient experience using best–worst scaling. *Psycho-Oncology* 2021;**30**(7):1104-11.

27. Maximum difference scaling: improved measures of importance and preference for segmentation. Sawtooth software conference proceedings; 2003. Sawtooth Software, Inc. Fir St., Sequim, WA.

28. StataCorp L. Stata statistical software: Release 17 (2021). *College Station, TX: StataCorp LP* 2021.

29. Howard K, Salkeld G, Pignone M, et al. Preferences for CT colonography and colonoscopy as diagnostic tests for colorectal cancer: A discrete choice experiment. *Value in Health* 2011;**14**(8):1146-52.

30. De Bekker-Grob EW, Rose JM, Donkers B, et al. Men's preferences for prostate cancer screening: A discrete choice experiment. *British Journal of Cancer* 2013;**108**(3):533-41.

31. Mansfield C, Ekwueme DU, Tangka FKL, et al. Colorectal Cancer Screening: Preferences, Past Behavior, and Future Intentions. *Patient* 2018:1-13.

32. Miles A, Taylor SA, Evans RE, et al. Patient preferences for whole-body MRI or conventional staging pathways in lung and colorectal cancer: a discrete choice experiment. *European radiology* 2019;**29**(7):3889-900.

33. Fitch MI, Deane K, Howell D, et al. Women's experiences with ovarian cancer: reflections on being diagnosed. *Canadian Oncology Nursing Journal/Revue canadienne de soins infirmiers en oncologie* 2002;**12**(3):152-59.

34. Jelicic L, Brooker J, Shand L, et al. Experiences and health care preferences of women with ovarian cancer during the diagnosis phase. *Psycho-oncology* 2019;**28**(2):379-85.

35. Hol L, De Bekker-Grob EW, Van Dam L, et al. Preferences for colorectal cancer screening strategies: A discrete choice experiment. *British Journal of Cancer* 2010;**102**(6):972-80.

36. van Dam L, Hol L, Bekker-Grob EWd, et al. What determines individuals' preferences for colorectal cancer screening programmes? A discrete choice experiment. *European Journal of Cancer* 2010;**46**(1):150-59.

37. Salkeld G, Ryan M, Short L. The Veil of Experience: Do Consumers Prefer What They Know Best? *Health Economics* 2000;**9**(3):267-70.

38. Finn A, Louviere JJ. Determining the appropriate response to evidence of public concern: the case of food safety. *Journal of Public Policy & Marketing* 1992;**11**(2):12-25.

39. de Bekker-Grob EW, Donkers B, Jonker MF, et al. Sample size requirements for discrete-choice experiments in healthcare: a practical guide. *The Patient-Patient-Centered Outcomes Research* 2015;**8**(5):373-84.

40. Naik G, Ahmed H, Edwards AG. Communicating risk to patients and the public. *British Journal of General Practice* 2012;**62**(597):213-16.

For peer review only - http://bmjopen.bmj.com/site/about/guidelines.xhtml

41. Drummond M, Torbica A, Tarricone R. Should health technology assessment be more patient centric? If so, how?: Springer, 2020.

Figure captions

Figure 1: Example of a choice task

Figure 2: Best-worst scaling results. The distance between attributes is a spatial representation of the difference in relative importance between attributes on the latent importance scale.

Figure 3: Sub-group analysis results comparing importance scores between those previously tested for ovarian cancer (n=40) and those who have never been tested (n=110). Error bars represent 95% confidence intervals

Figure 4: Subgroup analysis results comparing importance scores between those who have previously undergone a transvaginal ultrasound for any medical reason (n=50) and those who have not (n=100). Error bars represent 95% confidence intervals

For peer review only

For peer review only - http://bmjopen.bmj.com/site/about/guidelines.xhtml

Figure 1: Example of a choice task

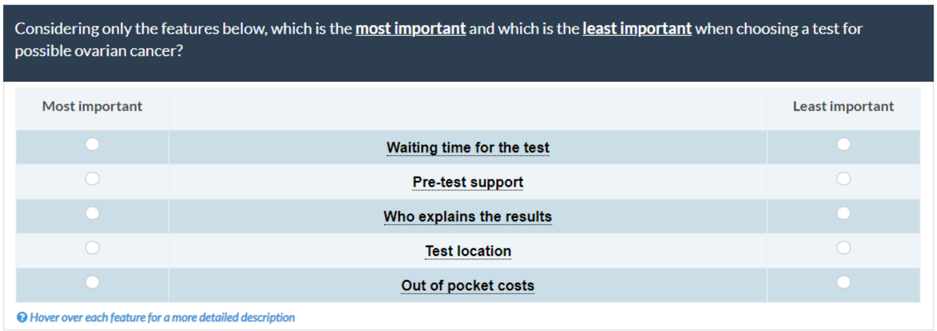
Considering only the features below, which is the **most important** and which is the **least important** when choosing a test for possible ovarian cancer?

Most important		Least important
○	Waiting time for the test	○
○	Pre-test support	○
○	Who explains the results	○
○	Test location	○
○	Out of pocket costs	○

Hover over each feature for a more detailed description

For peer review only

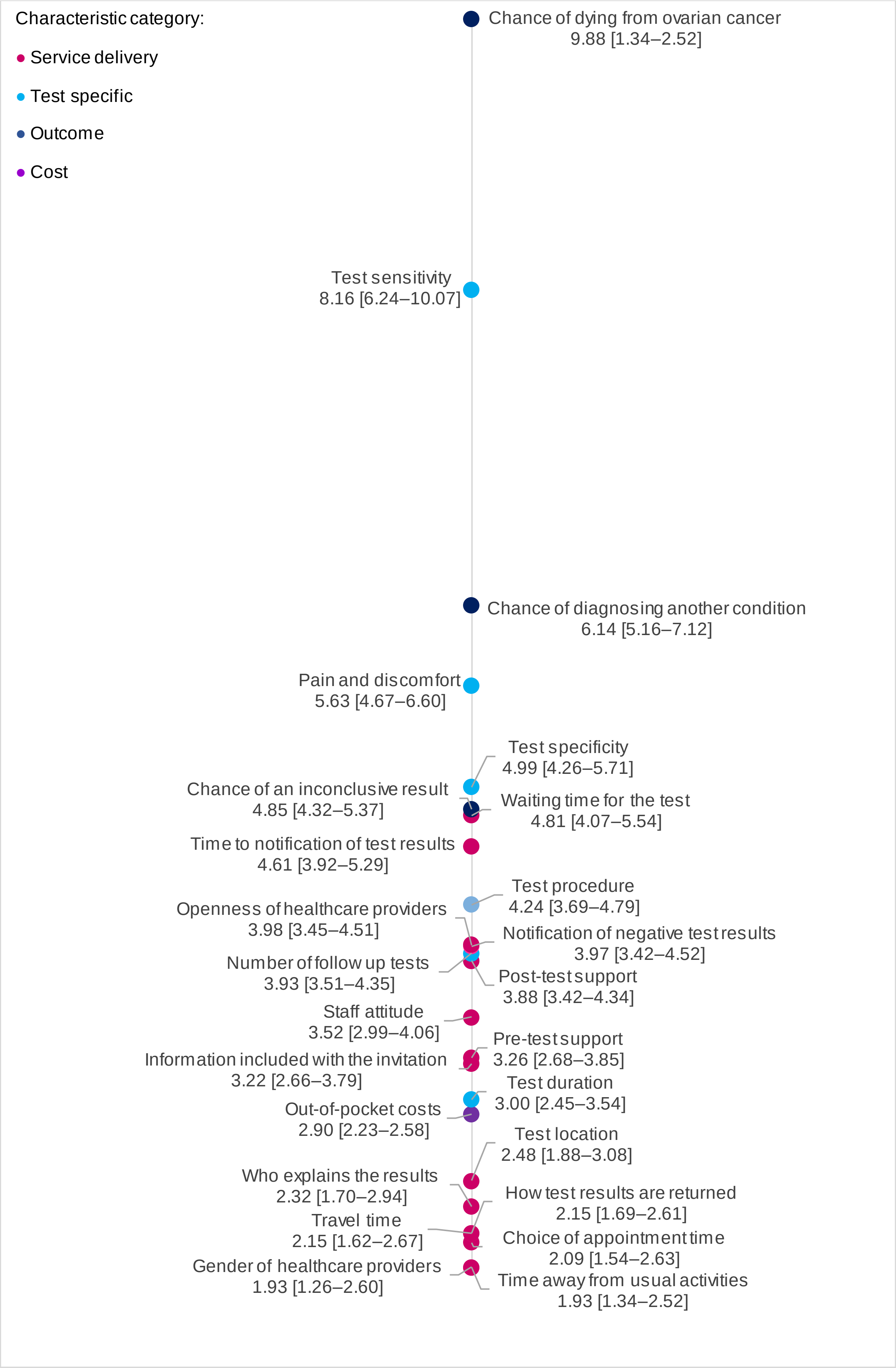
Characteristic category:
Service delivery
Test specific
Outcome
Cost
Chance of dying from ovarian cancer
9.88 [1.34–2.52]
Test sensitivity
8.16 [6.24–10.07]
Chance of diagnosing another condition
6.14 [5.16–7.12]
Pain and discomfort
5.63 [4.67–6.60]
Test specificity
4.99 [4.26–5.71]
Chance of an inconclusive result
4.85 [4.32–5.37]
Waiting time for the test
4.81 [4.07–5.54]
Time to notification of test results
4.61 [3.92–5.29]
Test procedure
4.24 [3.69–4.79]
Openness of healthcare providers
3.98 [3.45–4.51]
Notification of negative test results
3.97 [3.42–4.52]
Number of follow up tests
3.93 [3.51–4.35]
Post-test support
3.88 [3.42–4.34]
Staff attitude
3.52 [2.99–4.06]
Pre-test support
3.26 [2.68–3.85]
Information included with the invitation
3.22 [2.66–3.79]
Test duration
3.00 [2.45–3.54]
Out-of-pocket costs
2.90 [2.23–2.58]
Test location
2.48 [1.88–3.08]
Who explains the results
2.32 [1.70–2.94]
How test results are returned
2.15 [1.69–2.61]
Travel time
2.15 [1.62–2.67]
Choice of appointment time
2.09 [1.54–2.63]
Gender of healthcare providers
1.93 [1.26–2.60]
Time away from usual activities
1.93 [1.34–2.52]

Figure 3: Sub-group analysis results comparing relative importance scores between those previously tested for ovarian cancer (n=40) and those who have never been tested (n=110). Error bars represent 95% confidence intervals.

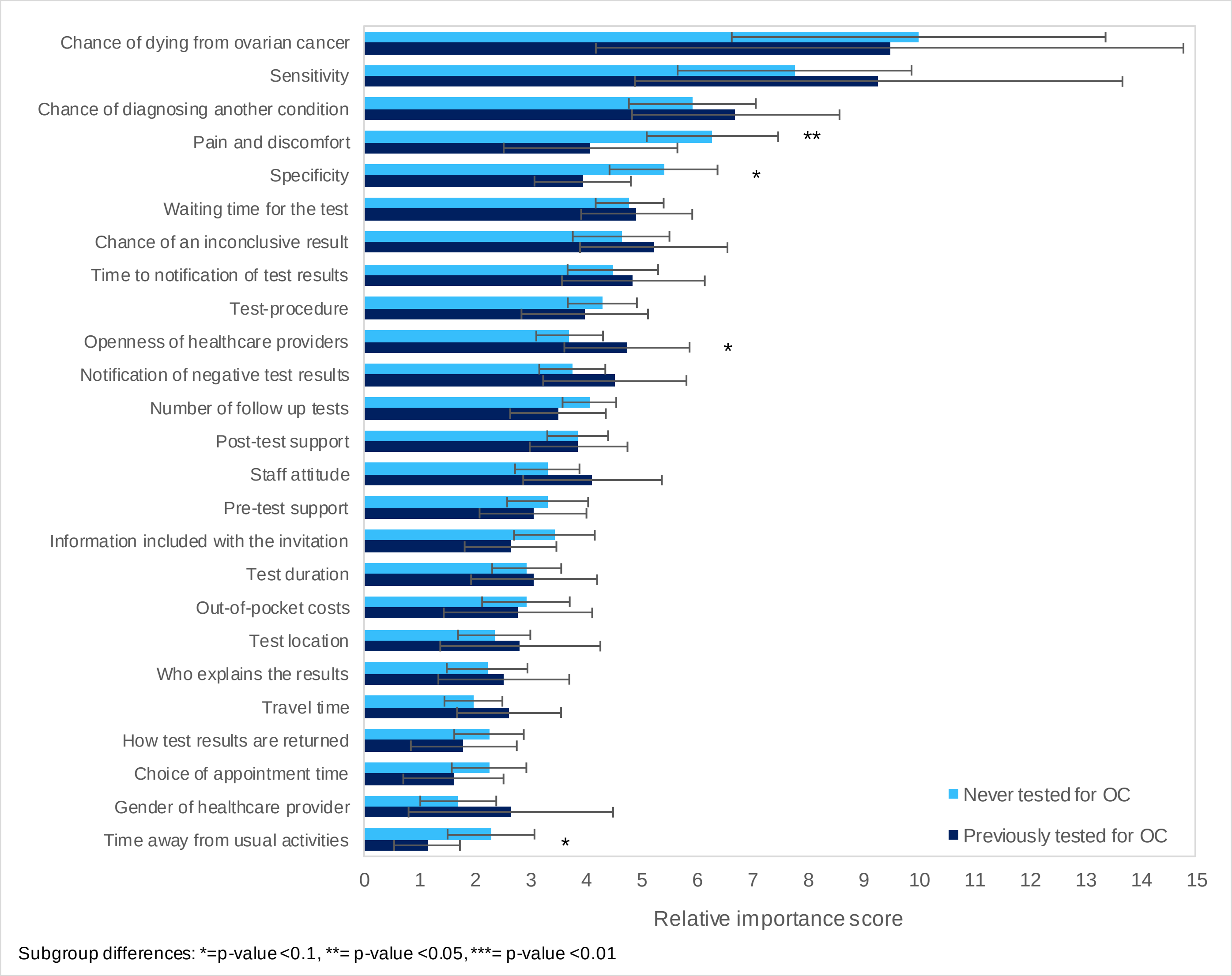

Figure 4: Subgroup analysis results comparing relative importance scores between those who have previously undergone a transvaginal ultrasound for any medical reason (n=50) and those who have not (n=100). Error bars represent 95% confidence intervals.

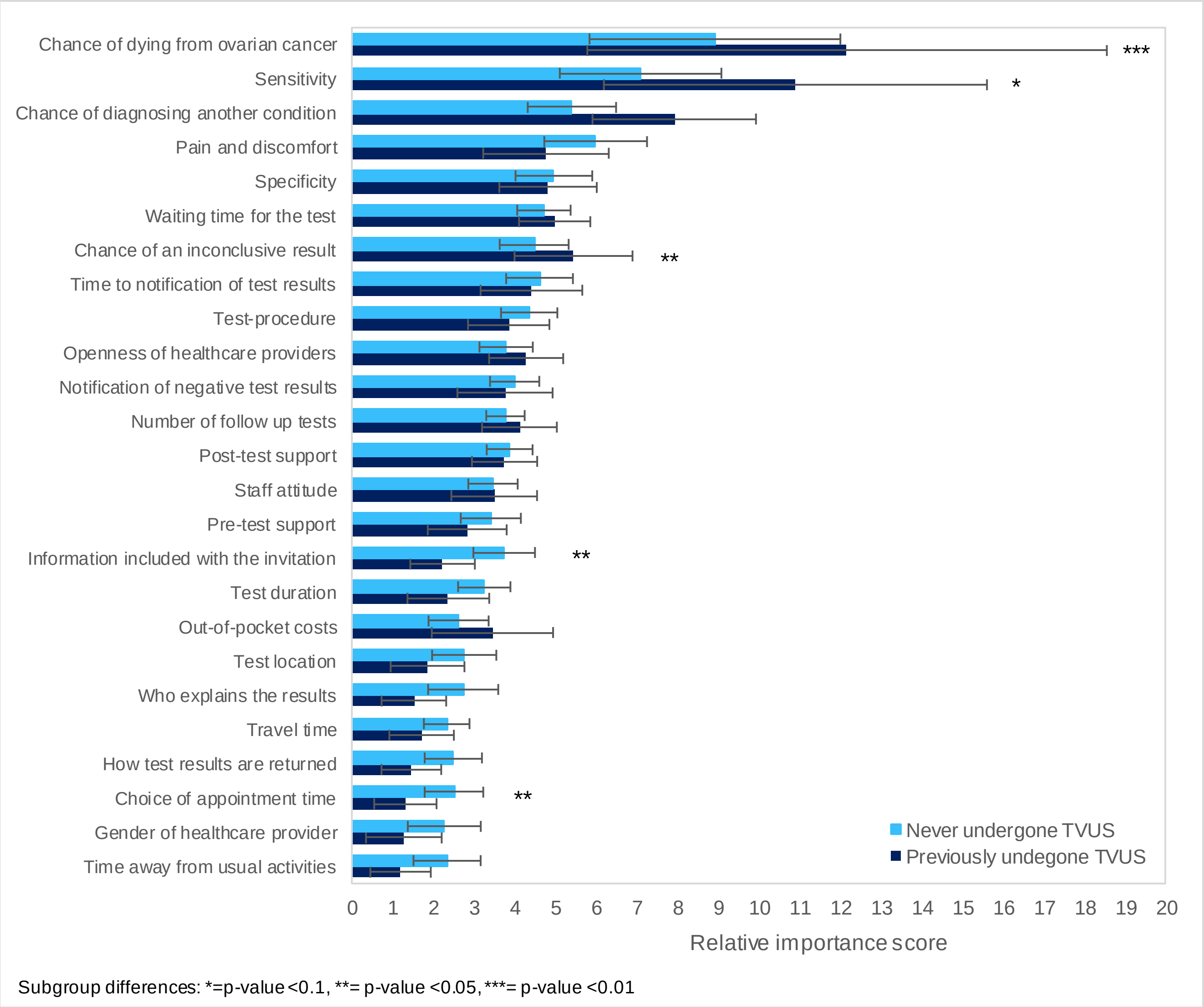

Appendix 1: Health-related characteristics of population

Characteristic	n (%)
**Self-reported overall health**	
Very good	18 (12)
Good	61 (41)
Fair	47 (31)
Poor	14 (9)
Very poor	4 (3)
Not reported	6 (4)
**Perceived risk of ovarian cancer**	
Very High	4 (3)
High	12 (8)
Average	83 (55)
Low	33 (22)
Very low	12 (8)
Not reported	6 (4)
**Ovarian cancer-related worry**	
A great deal	4 (3)
A lot	25 (17)
A moderate amount	43 (29)
A little	47 (31)
Not at all	26 (17)
Not reported	5 (3)
**Personal history of cancer**	17 (13)
**Knew someone who was diagnosed with ovarian cancer**	25 (17)
**Previously tested for ovarian cancer**	40 (27)
**Previously undergone a TVUS (any reason)**	50 (33)
**Cervical screening**	
Always attends/attended	75 (50)
Irregularly attends/attended	37 (25)
Never attended/stopped attending	37 (25)
Unknown	1 (1)
**How much confidence and trust do you have in GPs**	
A great deal/a lot	59 (39)
A moderate amount	53 (35)
A little	25 (17)
None at all	2 (1)
Unknown	11 (7)
**How much do you feel able to be involved in medical decisions**	
A great deal	12 (8)
A lot	22 (15)
A moderate amount	58 (39)
A little	34 (23)
Not at all	17 (11)
Unknown	7 (5)
**How much do you wish to be involved in medical decisions**	
A great deal	60 (40)
A lot	67 (45)
A moderate amount	19 (13)
A little	3 (2)
Not at all	1 (1)

For peer review only

Appendix 2: Full summary of raw best-worst scores, counting analysis and conditional logit results

Items	Most	Least	Most-Least	Counting: Standardised score	Counting: SD	Counting: 95% confidence interval	Conditional logit: Coefficient	Conditional logit: SE	Ranking
Sensitivity	410	133	277	0.308	0.60	0.21 – 0.40	1.44***	0.096	2
Chance of dying from ovarian cancer	504	162	342	0.380	0.70	0.26 – 0.49	1.63***	0.103	1
Choice of appointment time	122	316	-194	-0.216	0.47	-0.29 – (-0.15)	0.08	0.085	23
Who explains the results	150	306	-156	-0.173	0.52	-0.26 – (-0.09)	0.18*	0.087	20
Pain and discomfort	246	94	152	0.169	0.44	0.10 – 0.24	1.07***	0.084	4
Notification of negative test results	135	103	32	0.036	0.34	-0.02 – 0.09	0.72***	0.078	11
Chance of diagnosing another condition	289	107	182	0.202	0.45	0.13 – 0.27	1.16***	0.088	3
Pre-test support	111	147	-36	-0.040	0.33	-0.09 – 0.01	0.53***	0.078	15
Test-procedure	177	124	53	0.059	0.34	0.00 – 0.11	0.79***	0.081	9
Staff attitude	120	128	-8	-0.009	0.34	-0.06 – 0.05	0.60***	0.077	14
Post-test support	106	84	22	0.024	0.28	-0.02 – 0.07	0.70***	0.076	13
Time away from usual activities	138	358	-220	-0.244	0.56	-0.33 – (-0.15)	Ref	Ref	25
Specificity	239	125	114	0.127	0.42	0.06 – 0.19	0.95***	0.085	5
Travel time	107	292	-185	-0.206	0.44	-0.28 – (-0.14)	0.11	0.081	21
Time to notification of test results	194	112	82	0.091	0.37	0.03 – 0.15	0.87***	0.082	8
Openness of healthcare providers	134	101	33	0.037	0.32	-0.01 – 0.09	0.72***	0.78	10
Number of follow up tests	129	99	30	0.033	0.25	-0.00 – 0.07	0.71***	0.078	12
Chance of an inconclusive result	174	74	100	0.111	0.31	0.06 – 0.16	0.92***	0.077	6
Out-of-pocket costs	154	229	-75	-0.083	0.48	-0.16 – (-0.01)	0.41***	0.083	18
Gender of healthcare provider	182	401	-219	-0.243	0.67	-0.35 – (-0.14)	0.001	0.091	24
How test results are returned	104	290	-186	-0.207	0.40	-0.27 – (-0.14)	0.11	0.082	22
Test location	132	264	-132	-0.147	0.46	-0.22 – (-0.07)	0.25***	0.083	19
Test duration	95	161	-66	-0.073	0.32	-0.12 – (-0.02)	0.44***	0.077	17
Information included with the invitation	135	177	-42	-0.047	0.35	-0.10 – 0.01	0.51***	0.081	16
Waiting time for the test	213	113	100	0.111	0.37	0.05 – 0.17	0.91***	0.084	6

Key: Five best scoring attributes in each round, Five worst scoring attributes in each round. For the "least" column best and worst are inversed so worst scoring = rated least important most frequently

For peer review only

Appendix 3: Distribution of B-W scores across individuals. Demonstrating the degree of heterogeneity in preferences across individuals. Each attribute appeared within the survey six times meaning scores could range from 6 (always selected as most important) to -6 (always selected as least important).

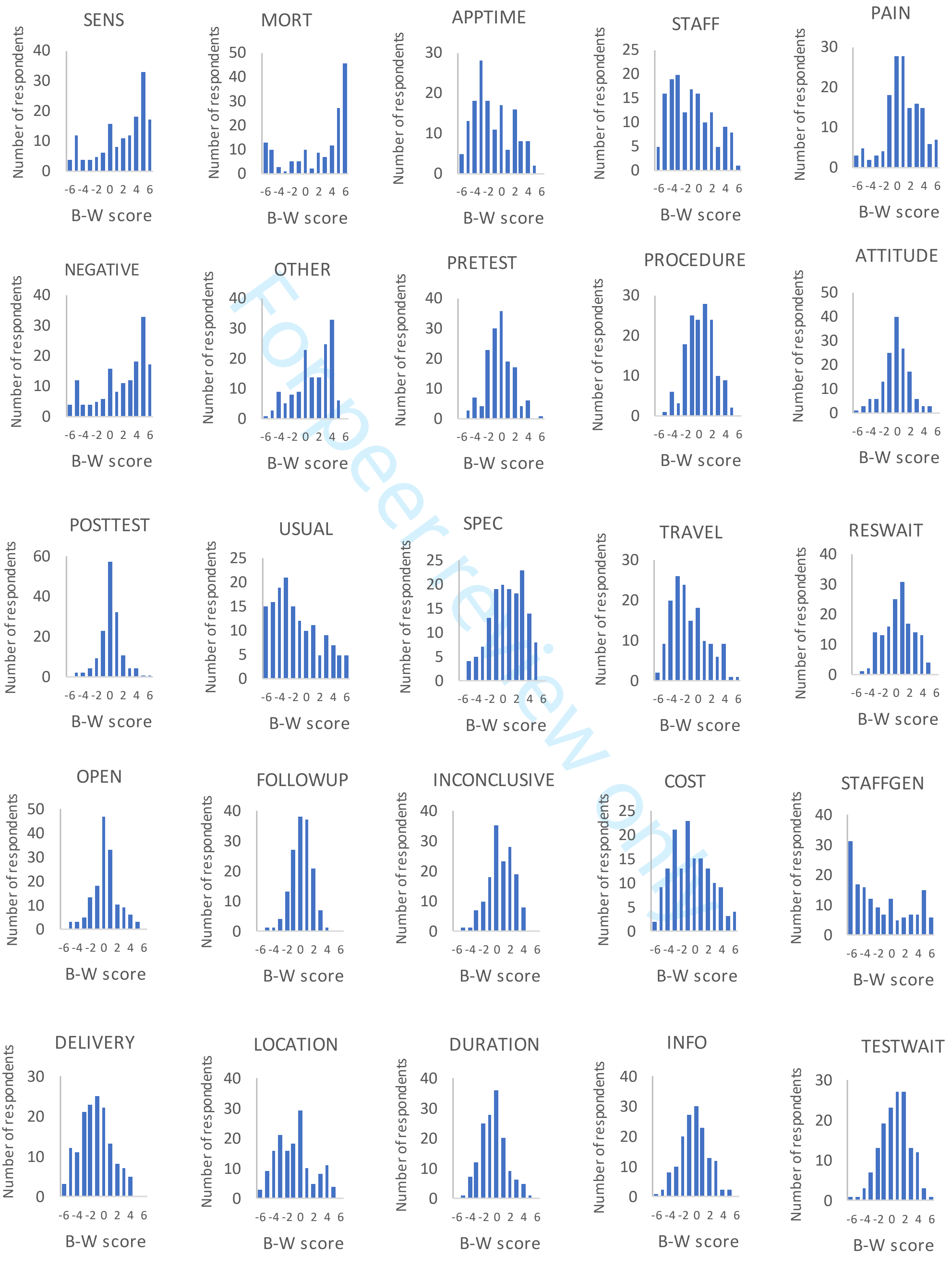

For peer review only

Appendix 4: Comparison of raw counting estimates and conditional logit estimates. Results were highly correlated

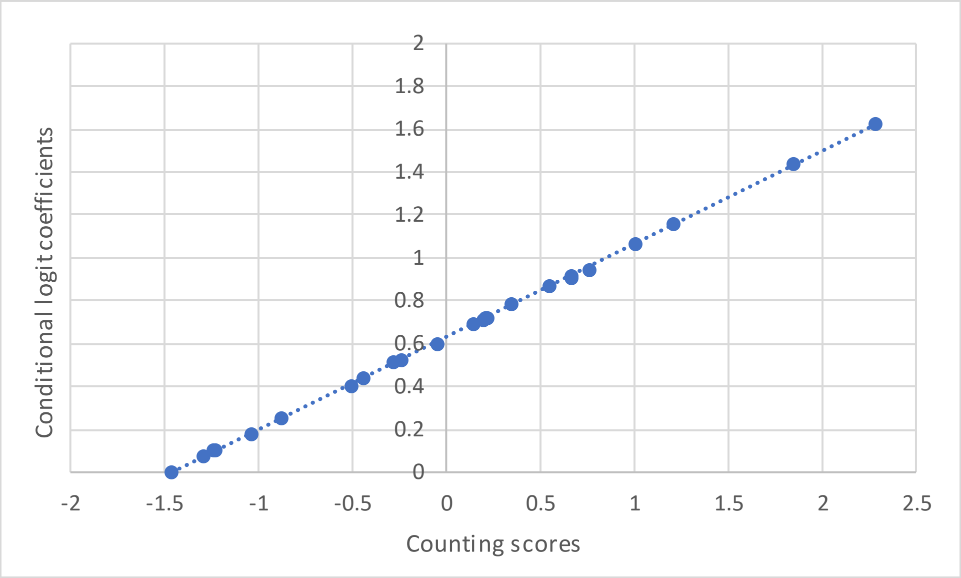

For peer review only

Appendix 5: Heteroscedastic logit estimates used to investigate differences in scale (i.e. error variance) between subgroups. A dummy variable representing previous test experience (either TVUS or OC testing) was included as an explanatory factor of the scale parameter. In both instances the scale term was not significant indicating observed differences in priorities between subgroups were not attributable to differences in error variance.

	OC test experience		TVUS experience	
	**Coefficient**	**SE**	**Coefficient**	**SE**
Items				
Sensitivity	1.63***	0.28	1.26***	0.35
Chance of dying from ovarian cancer	1.86***	0.30	1.47***	0.37
Choice of appointment time	0.13	0.17	0.07	0.14
Who explains the results	0.39*	0.21	0.29*	0.17
Pain and discomfort	1.13***	0.26	0.87***	0.27
Notification of negative test results	0.84***	0.19	0.68***	0.21
Chance of diagnosing another condition	1.30***	0.25	1.08***	0.26
Pre-test support	0.73***	0.18	0.58***	0.18
Test-procedure	1.06***	0.23	0.87***	0.24
Staff attitude	0.64***	0.20	0.56***	0.17
Post-test support	0.82***	0.19	0.68***	0.19
Time away from usual activities	Ref	Ref	Ref	Ref
Specificity	1.15***	0.24	0.22***	0.14
Travel time	0.23	0.19	0.86	0.23
Time to notification of test results	1.05***	0.22	0.64***	0.19
Openness of healthcare providers	0.74***	0.22	0.83***	0.22
Number of follow up tests	1.04***	0.21	0.85***	0.21
Chance of an inconclusive result	1.00***	0.21	0.41***	0.14
Out-of-pocket costs	0.44**	0.17	0.17***	0.18
Gender of healthcare provider	0.27	0.23	0.19	0.14
How test results are returned	0.21	0.20	0.15	0.14
Test location	0.18	0.18	0.26	0.15
Test duration	0.34**	0.16	0.44*	0.18
Information included with the invitation	0.58***	0.20	0.86**	0.22
Waiting time for the test	1.06***	0.21	0.22***	0.14
Scale factor				
Previous OC test (1=yes, 0=no)	-0.07	0.36		
Previous TVUS (1=yes, 0-no)			0.54*	0.32
Model statistics				
Log-likelihood	-3309.6			
Observations	31080		31080	
N	150		150	
Confidence levels: ***99%, ** 95%, *90%				

For peer review only
